# Extracellular vesicles from Kaposi Sarcoma-associated herpesvirus lymphoma induce long-term endothelial cell reprogramming

**DOI:** 10.1371/journal.ppat.1007536

**Published:** 2019-02-04

**Authors:** Ryan P. McNamara, Pauline E. Chugh, Aubrey Bailey, Lindsey M. Costantini, Zhe Ma, Rachele Bigi, Avery Cheves, Anthony B. Eason, Justin T. Landis, Kurtis M. Host, Jie Xiong, Jack D. Griffith, Blossom Damania, Dirk P. Dittmer

**Affiliations:** 1 Department of Microbiology and Immunology, Lineberger Comprehensive Cancer Center, University of North Carolina at Chapel Hill, Chapel Hill, North Carolina; 2 Q2 Solutions–EA Genomics, Morrisville, North Carolina; 3 Department of Biological and Biomedical Sciences, North Carolina Central University, Durham, North Carolina; Northwestern University, UNITED STATES

## Abstract

Extracellular signaling is a mechanism that higher eukaryotes have evolved to facilitate organismal homeostasis. Recent years have seen an emerging interest in the role of secreted microvesicles, termed extracellular vesicles (EV) or exosomes in this signaling network. EV contents can be modified by the cell in response to stimuli, allowing them to relay information to neighboring cells, influencing their physiology. Here we show that the tumor virus Kaposi’s Sarcoma-associated herpesvirus (KSHV) hijacks this signaling pathway to induce cell proliferation, migration, and transcriptome reprogramming in cells not infected with the virus. KSHV-EV activates the canonical MEK/ERK pathway, while not alerting innate immune regulators, allowing the virus to exert these changes without cellular pathogen recognition. Collectively, we propose that KSHV establishes a niche favorable for viral spread and cell transformation through cell-derived vesicles, all while avoiding detection.

## Author summary

The role of extracellular vesicles (EV) has received considerable attention in recent years. The contents of these cell-derived vesicles have been shown to be modulated upon challenge by a virus or neoplastic transformation, and can influence the behavior of recipient cells. Here we demonstrate that purified EV from an AIDS-associated, virus-driven lymphoma induces unique cellular signaling, motility, and gene expression reprogramming in recipient endothelial cells. This was accomplished without activation of innate immune activation, even after prolonged exposure to these EV. Collectively our results point toward a model in which tumor-derived EV condition neighboring cell physiology, while avoiding detection by immune regulators.

## Introduction

Extracellular communication is pivotal to maintain organismal homeostasis and a disease state. One of the mechanisms by which cells communicate to their surroundings is through extracellular vesicles (EV). Cells package a number of biological molecules into EV such as proteins, nucleic acids, lipids, and metabolites. EV are released into the microenvironment as well as into the circulation via the blood and lymphatic vessels. All vessels are lined with endothelial cells (EC), which are permanently exposed to EV, and uptake the EV-loaded cargo. This may induce changes in differentiation, metabolism, migration, and gene expression (reviewed in [1], for further examples see [2]). Ample experimental evidence has connected EV to cancer metastasis, immune signaling and the response to invading pathogens, though the molecular details of EV biology are far from established and tend to differ dramatically among experimental systems [1]. As in many aspects of modern biology it is difficult to come up with experimental approaches that are both tractable and physiologically relevant. Kaposi Sarcoma (KS) and Kaposi-sarcoma-associated herpesvirus (KSHV) lymphomas, specifically primary effusion lymphoma (PEL), represent one such system to study EV biology.

KS is one of the most angiogenic cancers in humans, and was one of the first identifiable markers for AIDS (reviewed in [3]). KSHV is the etiological agent of KS and PEL, which induce a unique tumor microenvironment that remodels tumor and lymphatic vasculature [4–6]. Of note, the transdifferentiation induced by the virus is as dramatic in uninfected neighboring cells as in virus-infected cells. We had shown that KSHV-infected cells release EV containing all viral micro RNAs (miRNA), but not the virus itself, and that the viral miRNAs are present at high concentrations in KSHV-lymphoma derived EV (KSHV-EV) in culture and in patients [7]. This establishes the KSHV-EV:EC interaction as physiologically relevant, experimentally robust, and as we show here, highly tractable.

EV membranes are enriched for phosphatidylserine (PS), which is recognized by Annexin-V and plays a role in EV adsorption. Tetraspanins, such as CD9, CD81, and CD63, are found on the majority of EV and have been used to affinity-purify EV [8–12]. Alix and Flotillins-1 and 2 are additional molecules that define EV (see http://www.exocarta.org/). Multiple EV purification methods have been devised [13]. These purification schemes yield comparable preparation of EV though the resultant fractions can be quite heterogeneous and need to be carefully characterized in each experiment. Exosomes are a subtype of EV that is defined by their intracellular biogenesis. Exosomes originate from the inward budding of the late endosome into the multivesicular body and traffic from there to the plasma membrane where they are released. When studies use primary patient material and/or cell culture supernatant, the origin of the EV cannot unequivocally be attributed to the multivesicular body; and the term EV rather than exosome is used. To date, no EV-specific receptors have been defined; evidence for tissue-specific uptake is limited to specific scenarios such as neuronal or immunological synapses [14]. Unlike viruses, EV are believed to be able to enter all cell types. Viruses that modulate EV maturation and content include Human and Simian immunodeficiency virus (HIV and SIV), vaccinia virus, hepatitis C virus, and herpesviruses [1, 7, 12]. This led Gould *et al*. to propose the trojan horse hypothesis [15], whereby viruses use EV to modulate the cell physiology of neighboring cells in order to further infection.

In this study, we sought to characterize how EV taken from the HIV-associated PEL, either cell culture or primary patient fluid, influence EC behavior. We discovered that affinity purified EV mediated cell migration, proliferation, and secretion of human interleukin-6 (IL-6) through the extracellular signal related kinases (ERK1/2) pathway. This was accomplished without tripping of innate sensors such as interferon regulatory factor 3 (IRF3), stimulator of interferon genes (STING) [16–21], or nuclear factor kappa B (NF-κB). It allows KSHV to modulate its immediate environment without alerting innate immune signaling pathways. Chronic exposure to PEL-derived EV, mimicking the pleural environment, resulted in a reprogramming of the recipient cells’ mRNA profile, without activating innate immune signaling cascades and/or interferon stimulatory genes. Collectively, these results provide a new paradigm for EV function in modifying the phenotype of recipient cells and highlight a previously unknown method of virus-induced cellular reprogramming without alerting viral sensors.

## Results

### EV from lymphoma cell lines, normal plasma, and primary tumor effusion are indistinguishable with regard to biophysical properties, fusion potential and marker protein content

**Fig 1:**
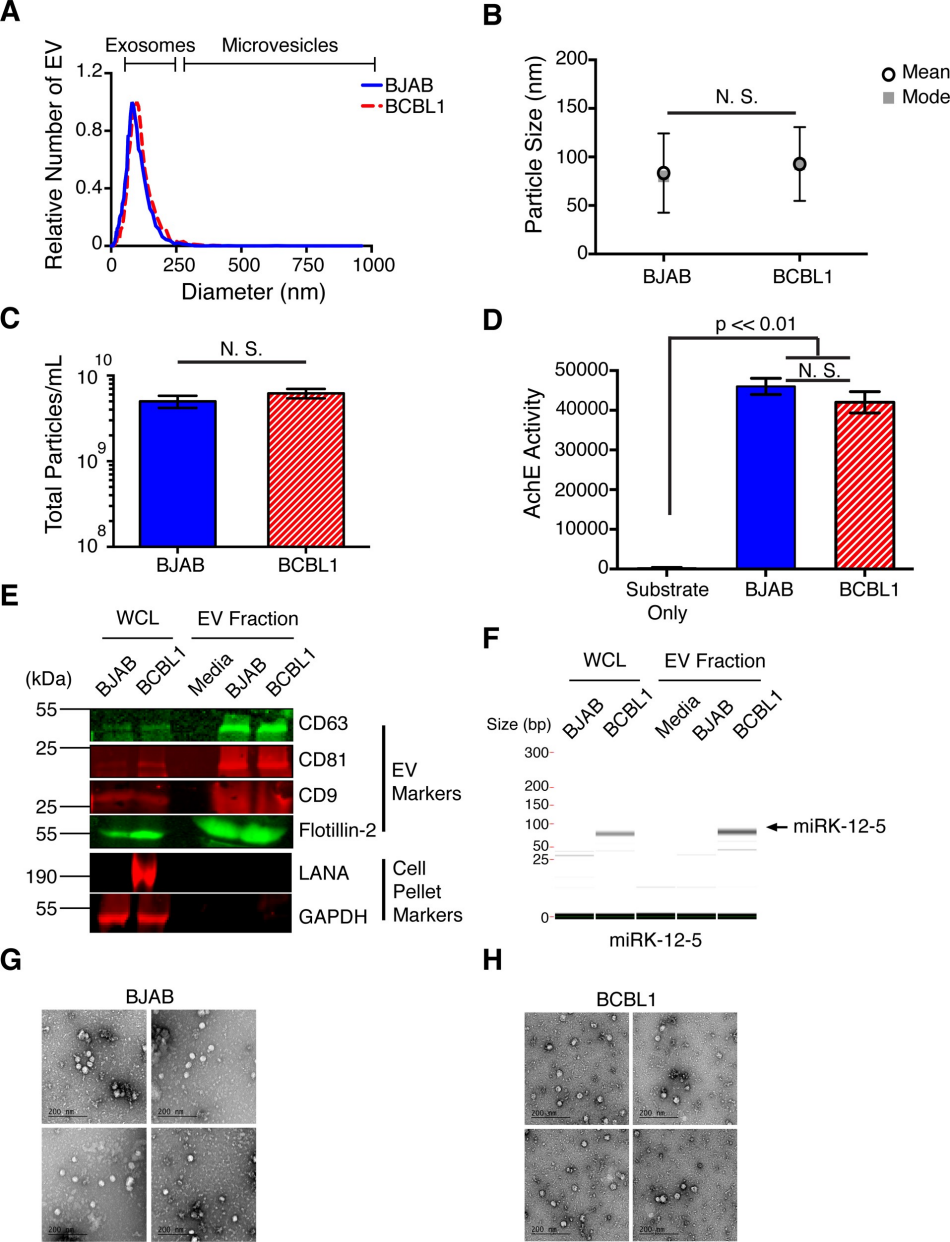
Analysis of total EV from cells. (A) Size distribution analysis of total EV isolated from the supernatant of BJAB (solid blue) or BCBL1 primary effusion lymphoma PEL (dashed red) cell. Size ranges of exosomes and microvesicles are shown (see also **S1 Fig**). (B) Mean (open circle) and mode (gray square) sizes of the total EV isolated from BJAB and BCBL1 PEL cell supernatant. (C) Total EV particles per mL of supernatant from BJAB (solid blue) or BCBL1 PEL (dashed red) cells. (D) Relative acetylcholine esterase (AchE) activity of the EV harvested. Substrate only is shown for reference against BJAB (solid blue) and BCBL1 PEL (dashed red) EV. (E) Western blot analysis of the total EV preparation. Whole cell lysates (WCL) and EV fractions were assayed for the presence of EV and cellular markers. The protein LANA was used as a cell isotype control for BCBL1. (F) Quantitative reverse transcriptase polymerase chain reaction (qRT-PCR) of the KSHV encoded miRK-12-5 is shown as run on the Caliper LabChip GX. The arrow points to the expected size of the amplified miRNA with the adapters (See also **S2–S5 Figs**). (G) Column purified EV from BJAB cell supernatant were viewed under electron microscopy via negative staining. (H) Column purified EV from BCBL1 PEL cell supernatant were viewed under electron microscopy via negative staining.

EV research is still a developing field. Functional studies are dependent on consistent and pure EV preparations. Hence, a carefully controlled purification strategy was established and validated. EV from a KSHV-negative B-cell lymphoma (BJAB) and a KSHV-positive primary effusion lymphoma (PEL) cell line (BCBL1) were compared to each other and to EV from primary KSHV-tumor effusions and normal plasma. To allow for large scale EV purification, an initial concentration step using polyethylene glycol (PEG) was used [13]. No discernable size or concentration differences were observed between EV isolated from BJAB and BCBL1 cell supernatant. The mean and mode sizes were <100 nm (**Fig 1A and 1B**), the particle concentrations were similar (**Fig 1C**), and acetylcholine esterase (AchE) activity was comparable across all preparations (**Fig 1D**). The biophysical properties of EV from PEL were similar to those of the Epstein-Barr virus (EBV)-negative B cell lymphoma BJAB, and the EBV-positive B cell lymphoma cell line Namalwa (**S1 Fig**).

Following established standards [22] the EV markers CD63, CD81, CD9, and Flotillin-2 were evaluated by Western blot (**Fig 1E**). All proteins were present at comparable levels in EV and none were detected in EV-depleted media. The KSHV latency-associated nuclear antigen (LANA) was present in BCBL1 lysate, but not BJAB lysate and not in EV. GAPDH was present in whole cell lysates, but not EV. MS/MS confirmed the presence of additional, prototypical EV markers, but no viral proteins. There was no difference in EV marker protein content as defined by exocarta.org among EV from BJAB vs BCBL1 (**S2 Fig**). KSHV-encoded miRNAs are incorporated at high concentrations into EV and provide a high sensitivity marker to trace BCBL1 derived EV [7]. The miRK-12-5 was present in BCBL1 cells and BCBL1-derived EV, but not BJAB cells or BJAB-derived EV (**Fig 1F**). Negative staining electron microscopy (EM) of EV showed small, rounded vesicles of ~40–70 nm diameter (**Fig 1G**). After PEG enrichment, EV were further purified by either ultracentrifugation or column purification. Both procedures yielded comparable results (**S3 Fig** and **S4 Fig**). In sum, this experimental approach resulted in clean and concentrated EV preparations.

To link the cell line studies to primary patient material, EV from healthy donor (HD) plasma and primary PEL fluid were isolated. The EV exhibited the same characteristic size distribution across different donors (**S5 Fig**). Their concentrations were at times higher than from culture supernatant. AchE activities, likewise, were comparable. These experiments demonstrate that EV isolated by this method from virus-negative and virus-positive cell supernatants were biophysically and biochemically similar to each other and to normal plasma or primary PEL fluid.

**Fig 2:**
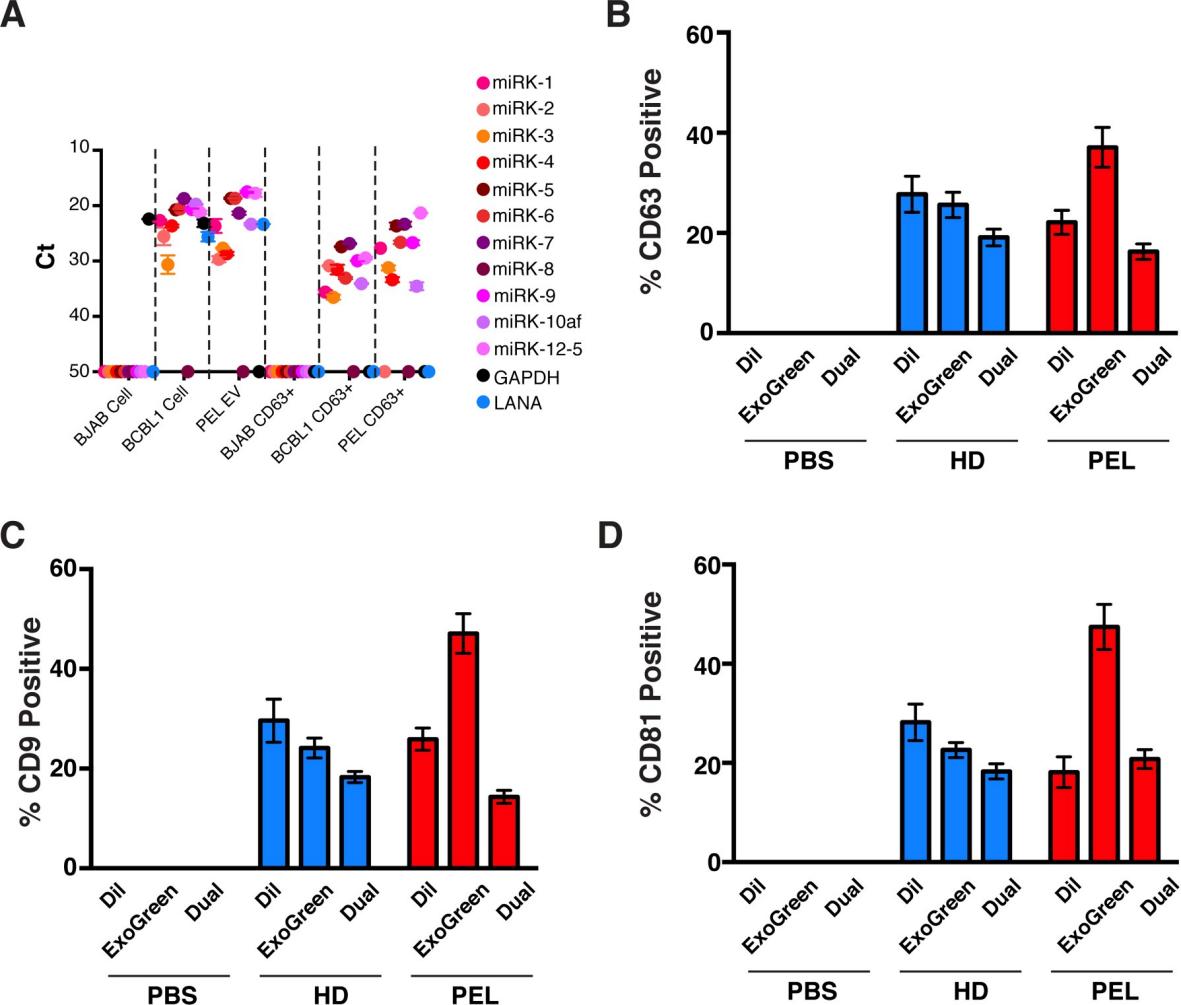
Affinity captured EV from BCBL1 cells contain KSHV-miRNA and tetraspanins. (A) qRT-PCR using stem-loop primers against 11 KSHV-encoded miRNAs was performed on cell pellet and CD63+ affinity purified EV fractions. We also used tested for intracellular *GAPDH* and KSHV *LANA mRNA* (cell isotype control) as controls. This was done in BJAB (non KSHV-infected) and BCBL1 (KSHV-infected PEL) cell lines (See also **S6 Fig**), as well as using primary PEL fluid EV and CD63+ affinity purified EV as control. (B) Percent positive fluorescent anti-CD63 beads were plotted for the PBS control, healthy donor (HD), and PEL samples (See also **S7 Fig**). (C) Percent positive fluorescent anti-CD9 beads were plotted for the PBS control, HD, and PEL samples (See also **S8 Fig**). (D) Percent positive fluorescent anti-CD81 beads were plotted for the PBS control, HD, and PEL samples (See also **S9 Fig**).

To exclude the presence of virion particles in the EV preparation, EV were subsequently affinity-purified using antibody conjugated beads. Recapitulating our prior results [7], this step retained EV-associated proteins (**S6A Fig**) and miRNAs (**S6B Fig**) while removing KSHV particles, as measured by DNA content (**S6C Fig**). NTA analysis of the affinity-purified EV from BJAB, BCBL1 PEL, HD, and primary PEL yielded consistent characteristics across (**S6D–S6F Fig**), and EM detected the presence of cup-like vesicles typically of EV, but no evidence of virion particles (**S6G and S6H Fig**). Through this method, we recovered all but one of the KSHV-encoded miRNAs in the CD63+ fraction of EV from BCBL1 cells. Neither the viral transcript LANA nor the cellular encoded GAPDH mRNA were present, demonstrating enrichment for the viral miRNAs, with primary PEL CD63+ EV serving as our positive control (**Fig 2A**). To show that the samples contained similar fractions of CD63+ EV, the EV were incubated with Dil, a membrane-intercalating dye, ExoGreen, an internal esterase-substrate, or both, bound to antibody-coated beads and subjected to flow cytometry (**S7 Fig**). Results were consistent between CD63 (**Fig 2B**), CD9 (**Fig 2C** and **S8 Fig**), and CD81 beads (**Fig 2D** and **S9 Fig**). This demonstrated that PEL derived EV carry at least three tetraspanin markers (CD63, CD81, CD9), which are suitable for positive affinity purification and that our three-step approach (PEG > column > affinity-bead) yielded a highly purified, virus and DNA-free, EV fraction.

**Fig 3:**
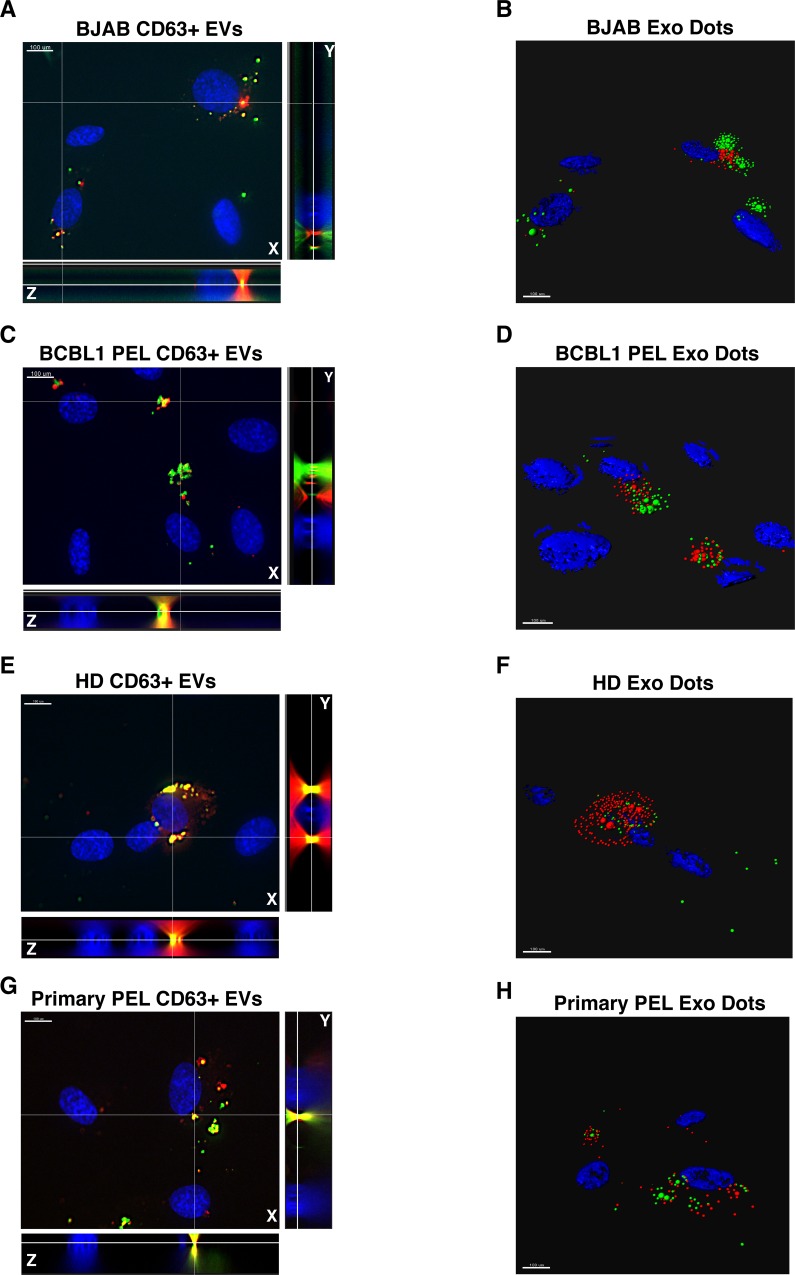
Labeled EV are capable of Being Uptaken by hTERT-HUVECs. (A) CD63-captured and dual-labeled (ExoGreen + Dil) EV from BJAB cells were added to hTERT-HUVEC for 12 hours and cells were assayed for uptake by fluorescence microcopy. Resulting 3-D images were taken and X, Y, and Z planes are shown. The white lines indicate the Y (right) and Z axis (bottom). Scale bar = 100 μm. (B) 3-D dot representation of image in (A). Regions of ExoGreen and Dil signal from (A) were converted in Imaris to weighted dots to show the delivered EV contents in the endothelial recipient cells. Weights of fluorescence were set using program-recommended thresholds, which changed from image to image. (C) Same as (A), but for EV taken from BCLB1 PEL cells added to hTERT-HUVECs. (D) Same as (B), but for EV taken from BCBL1 PEL cells added to hTERT-HUVECs. (E) CD63-captured EV from the sera of a healthy donor were added to hTERT-HUVECs for 12 hours and imaged as in (A). (F) Same as (B), but for EV taken from a healthy donor and added to hTERT-HUVECs. (G) Same as (E), but for EV taken from primary PEL fluid and added to hTERT-HUVECs. (H) Same as (B), but for EV taken from primary PEL fluid and added to hTERT-HUVECs (see also **S10** and **S11 Figs**).

**Fig 4:**
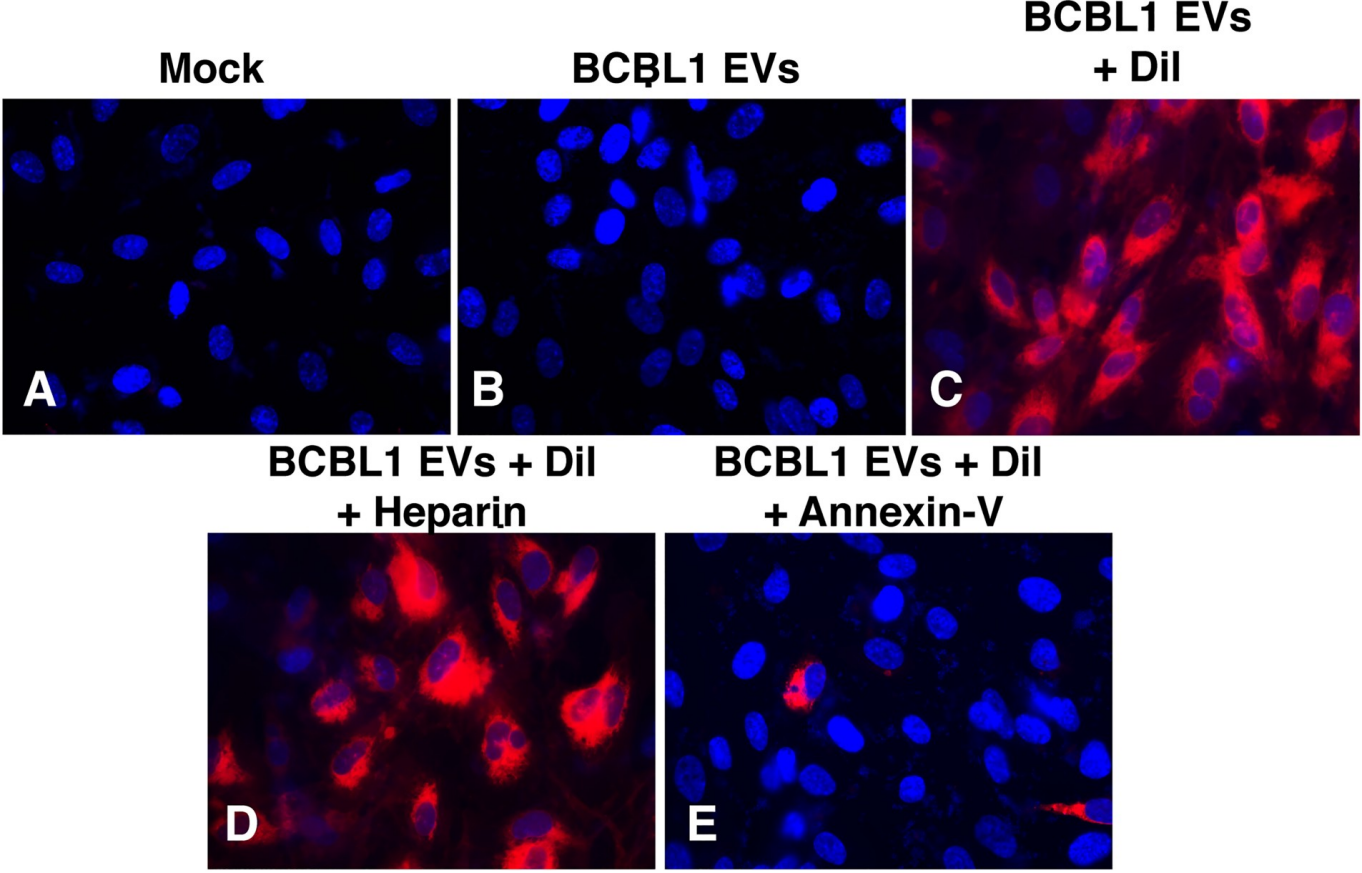
Cells treated with Annexin-V inhibit EV uptake. (A) hTERT-HUVECs were treated with mock labeled EV. (B) hTERT-HUVECs were treated with mock labeled BCBL1 EV and then imaged 24 hours later. (C) hTERT-HUVECs were treated with Dil-labeled BCBL1 EV and then imaged 24 hours later. (D) hTERT-HUVECs were pre-treated with heparin and then treated with Dil-labeled BCBL1 EV and then imaged 24 hours later. (E) hTERT-HUVECs were pre-treated with Annexin-V and then treated with Dil-labeled BCBL1 EV and then imaged 24 hours later.

The principal target of transformation by KSHV are endothelial cells (EC, reviewed in [3]). EC are also exposed to the highest concentration (~ 10^11^/mL) of EV via lymphatic and blood vessels and thus can be considered a bona-fide target of systemically circulating EV [23]. For these reasons, hTERT-immortalized human umbilical vein endothelial cells (hTERT-HUVEC) were used to investigate EV uptake. To establish that the purified EV were endocytic, 10^10^ CD63+ EV were labeled with Dil (1,1’-dioctadecyl-3,3,3’3’-tetramethylindocarbocyanine perchlorate, red) and ExoGreen (green) and added to 10^5^ hTERT-HUVECs (MOI = 10^5^) and after 12 hours, fixed and analyzed by fluorescence microscopy. Uptake was equivalent for EV from all sources: BJAB, BCBL1, HD, and primary effusion fluid (**Fig 3**). The red and green labels co-localized in the cytoplasm. Not every incidence of red and green EV labels co-localized after cells were exposed for 12 hours. This was likely due to differences in the recycling nature of the biomolecules stained (lipids vs. proteins). As the time course was extended (**S10 Fig** and **S11 Fig**) the lipid dye Dil redistributed throughout the cell, in contrast to the ExoGreen protein dye which remained in punctate structures. EV uptake was blocked by Annexin-V, but not heparin sulfate at a concentration, which reliably blocks KSHV entry [24] (**Fig 4**). This establishes that, after extensive purification, the EV retained endocytic activity, which was dependent on phosphatidylserine (PS) but not heparin. Henceforth, this material is referred to as KSHV-EV.

### KSHV-EV enhance endothelial cell proliferation and migration

**Fig 5:**
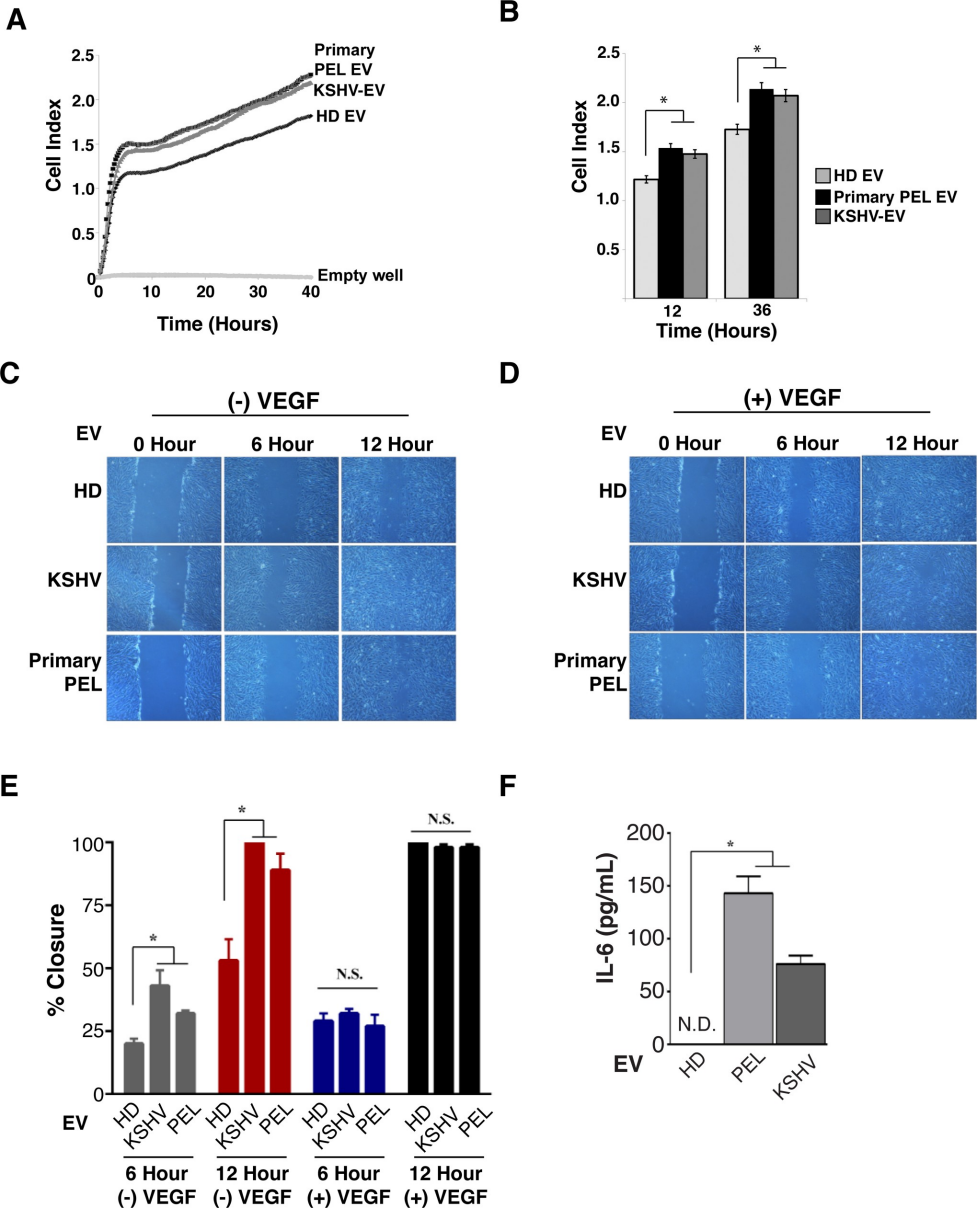
PEL EV Accelerate cell migration and wound healing and secretion of the PEL-Associated cytokine IL-6. (A) KSHV-EV or primary PEL EV accelerate cell migration across a transwell. hTERT-HUVEC cells were plated in a specialized xCelligence CIM-plate above a chamber containing EV from HD EV, KSHV, or primary PEL. (B) 12 and 36 hour time points were chosen to show differences in cell migration (cell index) (See also **S12 Fig**). (C) KSHV-EV or primary PEL EV accelerates wound healing. hTERT-HUVECs were grown to confluency and an artificial wound was created using a scratch. 2 hours prior to the scratch, cells were incubated with HD EV, KSHV-EV, or primary PEL EV. Images were taken immediately after the scratch (0 hour), 6 hour, and 12 hours in the absence of VEGF. (D) Same as (C), except with the addition of VEGF as a control (E) Quantification of wound closure at 6 and 12 hours with the various treatments. Wound boundaries from (C) and (D) were imaged using ImageJ for quantification (F) KSHV-EV or primary PEL EV induce IL-6 secretion. hTERT-HUVECs were incubated with EV from HD, KSHV, or primary PEL for 24 hours and cell supernatants were harvested and levels of IL-6 were determined by ELISA (See also **S13 Fig**).

To test the hypothesis that KSHV-EV act as chemoattractants and paracrine growth factors for EC, cell migration was monitored continuously using the xCelligence system. The xCelligence system allowed us to evaluate a number of physiological phenotypes that are associated with EV reprogramming. First, KSHV-EV served as a chemoattractant for EC migration, whereas EV from human donor blood (HD) did not. This chemoattractant property was phenocopied by EV isolated from primary PEL fluid (Primary PEL EV) (**Fig 5A and 5B**). Second, we asked if synergy existed between KSHV-EV and KS-relevant cytokines (VEGF, IL-6, PDGF-β, or SDF1-α). The hTERT-HUVECs were exposed to EV and plated into the top chamber and monitored for migration into the bottom chamber, which contained the cytokine in serum-free media. KSHV-EV significantly enhanced cell migration in response VEGF and IL-6, but not PDGF-β or SDF1-α (**S12 Fig**). Third, a scratch assay was performed. The hTERT-HUVECs were grown in the presence of EV, the confluent monolayer disrupted, and closure monitored over time. KSHV-EV and primary PEL EV enhanced migration, EV from HD did not (**Fig 5C**). The positive control, VEGF, alone or added to EV from HD yielded the same degree of scratch closures as induced by KSHV-EV (**Fig 5D and 5E**). Fourth, to test the hypothesis that EV induced cytokines, which could amplify or mediate the migration phenotype, supernatants were analyzed for IL-6, IL-10, which are implicated in KS biology, as well as the immune response cytokines IL-18, IL-1β, and interferon alpha (IFN-α). Only IL-6 was significantly induced in response to KSHV-EV and primary PEL EV (**Fig 5F** and **S13 Fig**). These experiments establish that KSHV-EV, but not EV circulating in healthy patients contributes to the pathophysiology of KS by modulating EC function and inducing human IL-6.

### KSHV-EV do not activate innate immune signaling pathways

**Fig 6:**
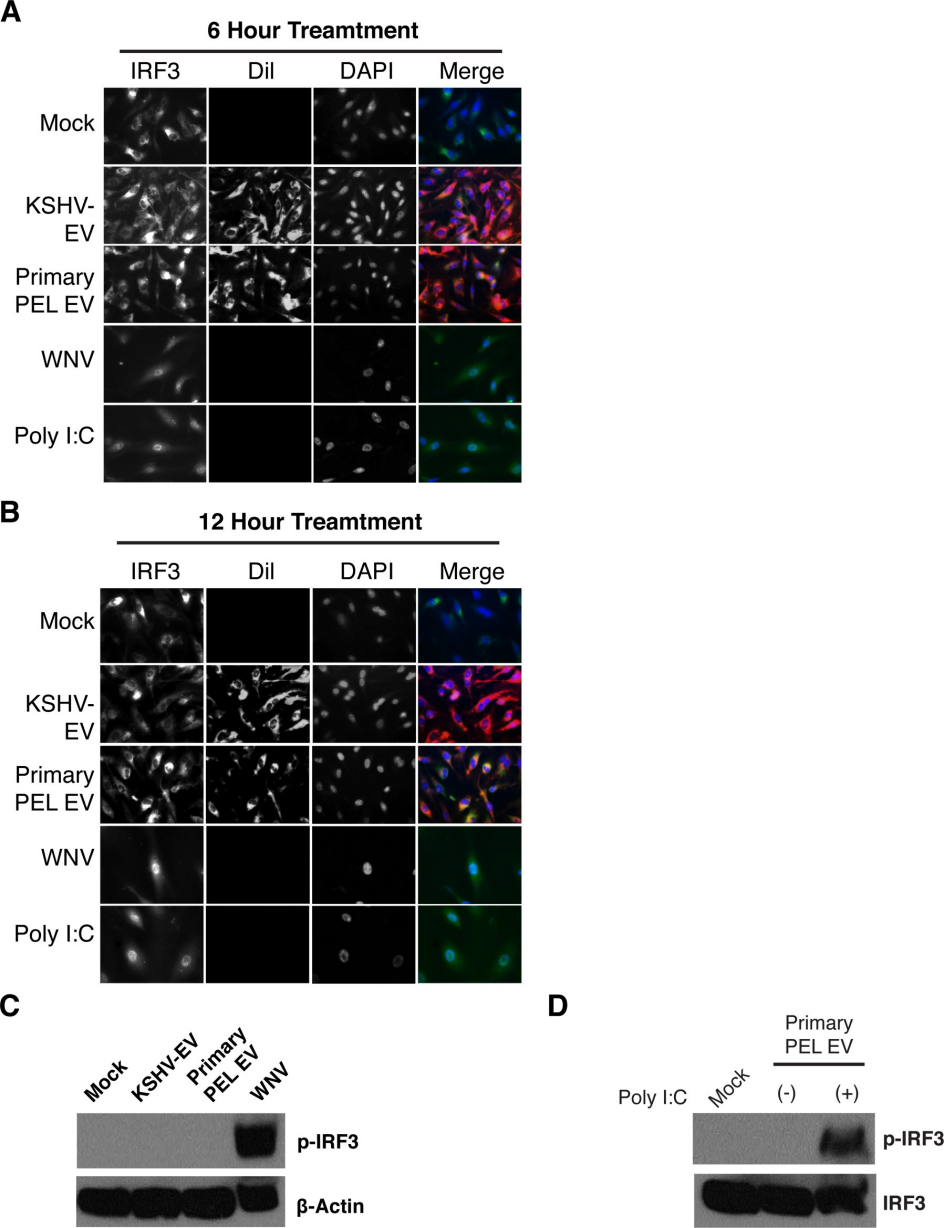
PEL EV do not trigger innate immune regulators. (A) PEL EV do not induce translocation of IRF3. hTERT-HUVECs were treated with KSHV-EV or primary PEL EV and translocation of the transcription factor IRF3 was monitored through immunofluorescence. Infection with West Nile virus (WNV) or treatment with the double stranded RNA mimic Poly I:C were used as controls. (B) Same as (A), but for an extended time course (12 hours vs 6 hours). (C) PEL EV do not induce phosphorylation (activation) of IRF3. hTERT-HUVECs were treated with KSHV-EV or primary PEL EV and phospho-IRF3 was assayed for. As a control, cells were separately treated with WNV (See also **S14** and **S15 Figs**).(D) IRF-3 can be phosphorylated through Poly I:C treatment after treatment with Primary PEL-EV.

To test whether KSHV-EV elicited an innate immune response in EC, a number of known innate immune signaling pathways were examined. On the one hand, such a phenotype would be expected as PEL and KS represent an inflammatory microenvironment (reviewed in [3]); on the other hand, pro- as well as anti-inflammatory phenotypes have been reported for infected-cell derived EV from different viruses (reviewed [1]). The innate immune response involves membrane-bound receptors, such as toll-like receptors (TLRs), as well as cytoplasmic RIG-like receptors, such as RIG-I and MDR-5. Both pathways ultimately converge onto interferon regulatory factor 3 (IRF3) and nuclear factor kappa B (NF-κB). IRF3 and NF-κB are normally sequestered in the cytoplasm. Upon stimulation, they translocate to the nucleus. KSHV-EV, or primary PEL EV did not induce nuclear translocation of IRF3 (**Fig 6A and 6B**) and did not induce IRF3 phosphorylation (**Fig 6C**). This was in contrast to infection with West Nile Virus or stimulation with Polyinosinic:polycytidylic acid (PolyI:C). KSHV-EV did not inhibit the phosphorylation of IRF3 in response to PolyI:C (**Fig 6D**), suggesting the KSHV EV did not actively inhibit signaling. KSHV-EV, or primary PEL EV did not induce nuclear translocation of NF-κB either (**S14 Fig**). Likewise, targeted transcriptional profiling of ninety NF-κB-regulated genes showed no response to KSHV-EV (**S15 Fig**). The IRF3 and NF-κB transcription factors represent the endpoint of a multitude of RNA sensing pathways. As neither was activated, it is unlikely that any of the upstream receptors (TLR, RLR) were activated to the degree that authentic viral infection would.

**Fig 7:**
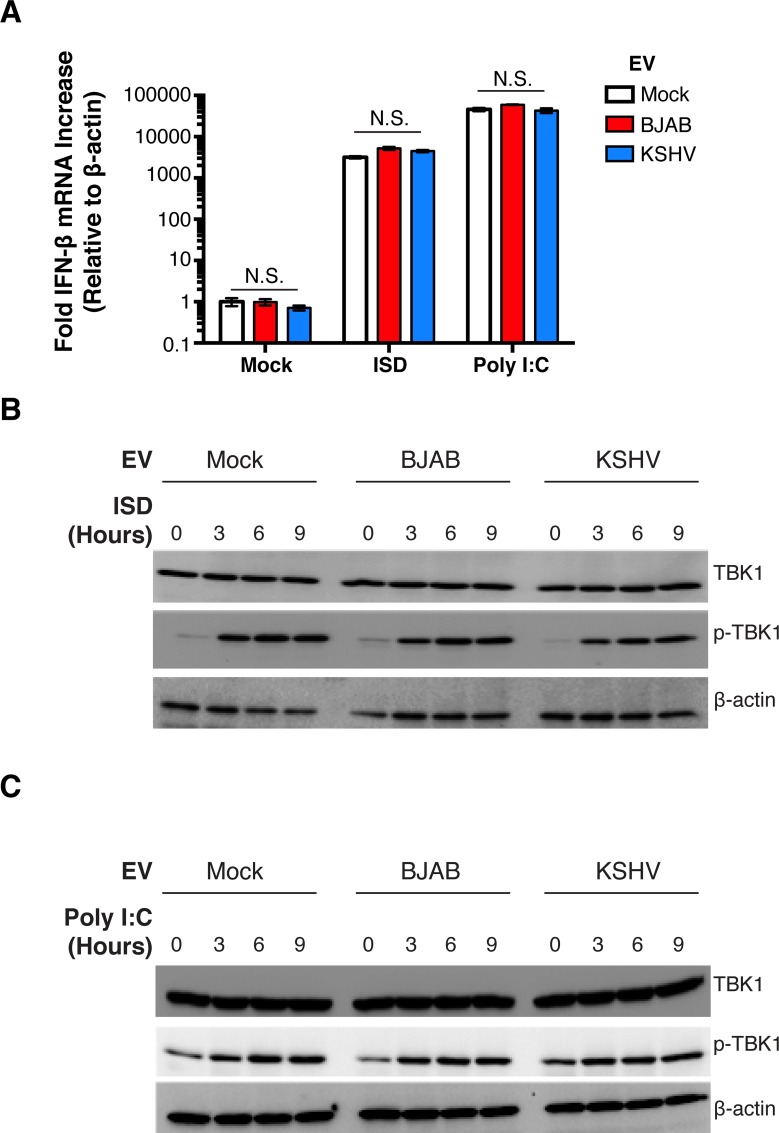
PEL EV do not Activate nor Antagonize *IFN-β* Expression or STING activation in response to stimulation. (A) hTERT-HUVECs were treated with BJAB (control) EV or KSHV-EV (or mock treated), and subsequently with mock, interferon stimulatory DNA fragment (ISD), or Poly I:C. Relative *IFN-β* mRNA levels were quantified and standardized to *β-Actin* [25]. (B) PEL EV do not Inhibit Phosphorylation of TBK1 in Response to ISD Stimulation. hTERT-HUVECs were treated with BJAB (control) EV or KSHV-EV (or mock treated), and subsequently with mock or ISD. Cells were lysed at various time points post stimulation and contents were run out for western blot analysis for total TBK1, phosphorylated TBK1 (p-TBK1), and β-Actin as a loading control. (C) PEL EV does not Inhibit Phosphorylation of TBK1 in Response to Poly I:C Stimulation. hTERT-HUVECs were treated with BJAB (control) EV or KSHV-EV (or mock treated), and subsequently with mock or Poly I:C. Cells were lysed at various time points post stimulation and contents were run out for western blot analysis for total TBK1, phosphorylated TBK1 (p-TBK1), and β-Actin as a loading control.

KSHV is a DNA virus, and its reactivation from latency is curbed by cGAS/STING [25]. To test for the induction of cGAS-STING signaling by KSHV-EV, we monitored the induction of interferon-beta (IFN-β). KSHV-EV did not change IFN-β transcript levels, and KSHV-EV did not modulate the cGAS/STING response to Interferon Stimulatory DNA (ISD) or Poly I:C (**Fig 7A**). As a second, independent measure of cGAS/STING activation we measured TANK binding kinase (TBK). TANK is phosphorylated upon recognition of cytosolic nucleic acids in a MAVS dependent manner, and physically interacts with STING. KSHV-EV did not affect phospho-TBK levels alone or in conjunction with the positive inducers (**Fig 7B and 7C**). In sum, neither TLR, RIG-I, nor cGAS/STING pathways were activated by KSHV-EV; at least under these conditions KSHV-EV did not inhibit the activation of these pathways by physiological triggers either.

### KSHV-EC activate ERK1/2

**Fig 8:**
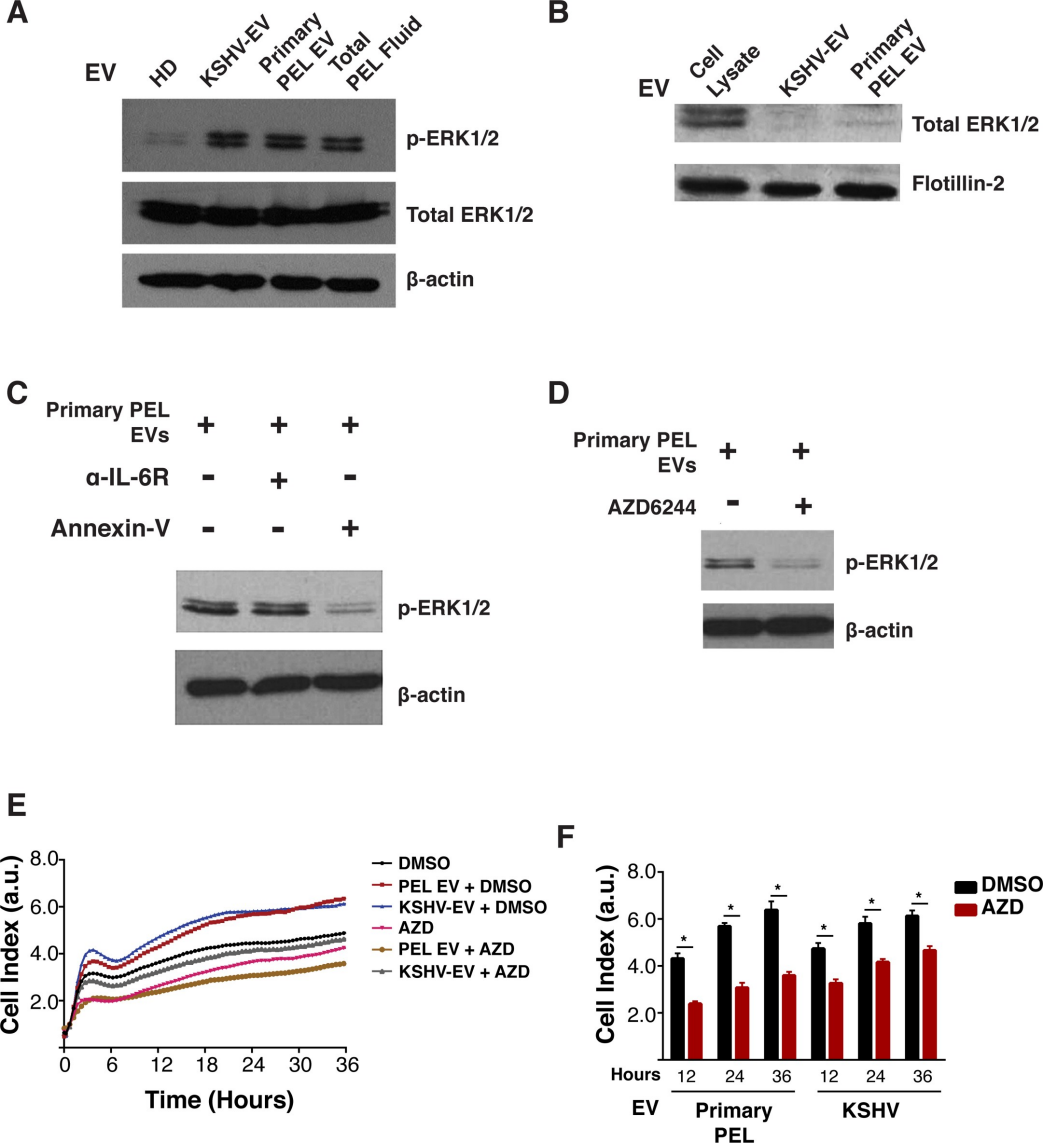
PEL EV signal through the canonical MEK/ERK1/2 pathway. (A) PEL EV activate phosphorylation of the signal transducer ERK1/2. hTERT-HUVECs were treated with HD EV, KSHV-EV, or primary PEL EV and levels of phospho-ERK1/2 (p-ERK1/2) were assayed. As a control, cells were treated with unfiltered primary PEL fluid. (B) PEL EV do not deliver ERK1/2 to recipient cells. KSHV-EV or primary PEL EV were lysed and assayed for the presence of ERK1/2. Cell lysate was used a control. (C) EV, and not secreted IL-6, induce phosphorylation of ERK1/2. hTERT-HUVECs were treated with primary PEL EV as in (A) in the presence of the blocking agents to the IL-6 receptor or Annexin-V. The IL-6 blocking agent serves as a control to monitor if EV stimulated release of IL-6 was activating ERK1/2; Annexin-V is an EV blocking agent.(D) ERK1/2 is activated by EV through the canonical MEK/ERK pathway. hTERT-HUVECs were treated with DMSO (-) or the MEK inhibitor AZD6244 for 4 hours and subsequently with primary PEL EV as in (A). (E) Inhibition of the MEK/ERK signaling pathway inhibits EV-dependent cell migration. hTERT-HUVECs seeded in a specialized xCelligence CIM plate in the presence of DMSO or AZD6244. 6 hours later, bottom well media was replaced with media containing HD EV, KSHV-EV, or primary PEL EV. (F) Quantitation of cell migration in (E) at 12, 24, and 36 hours post exposure to HD EV, KSHV-EV, or primary PEL EV in the presence of DMSO (black) or AZD6244 (red) (see also **S16 Fig**).

To identify molecular pathways that could explain KSHV-EV induced endothelial cell migration, we explored ERK1/2 signaling. ERK1/2 has been implicated in IL-6 signaling as well as EC migration [26, 27]. Treatment of hTERT-HUVEC with KSHV-EV and primary PEL EV, but not HD EV induced ERK1/2 phosphorylation (p-ERK1/2) (**Fig 8A**). The EV themselves did not contain p-ERK1/2 (**Fig 8B**). To exclude the possibility that IL-6 induced ERK1/2 phosphorylation as part of a secondary feedback loop, the experiment was repeated in the presence of antagonistic anti-IL-6 receptor antibodies. Despite blocking IL-6 signaling, ERK1/2 became phosphorylated upon KSHV-EV exposure (**Fig 8C**). Pre-incubation with Annexin-V, which blocks EV adsorption, significantly reduced p-ERK1/2 levels (**Fig 8C**). This result is consistent with the notion that ERK activation was a direct result of EV exposure.

ERK1/2 is phosphorylated by MEK, which can be targeted pharmacologically. To test the hypothesis that MEK kinase activity was required for p-ERK1/2 in response to KSHV-EV and primary PEL EV, we used AZD6244. Pre-treatment of cells with AZD6244 blocked ERK1/2 activation by primary PEL EV relative to DMSO control (**Fig 8D**). AZD6244 also reduced primary PEL EV- and KSHV-EV-dependent cell migration (**Fig 8E and 8F).** To ensure that AZD6244 did not exert off-target effects, we repeated the cell migration assay with a different MEK inhibitor, PD184352. Treatment with PD184352 antagonized the enhanced cell migration phenotype of hTERT-HUVECs treated with KSHV-EV (**S16 Fig**). These experiments demonstrate that KSHV-EV activate the Ras/Raf/MEK/ERK pathway, which leads to EC activation, proliferation, and migration.

### KSHV-EV stably reprograms endothelial cells

**Fig 9:**
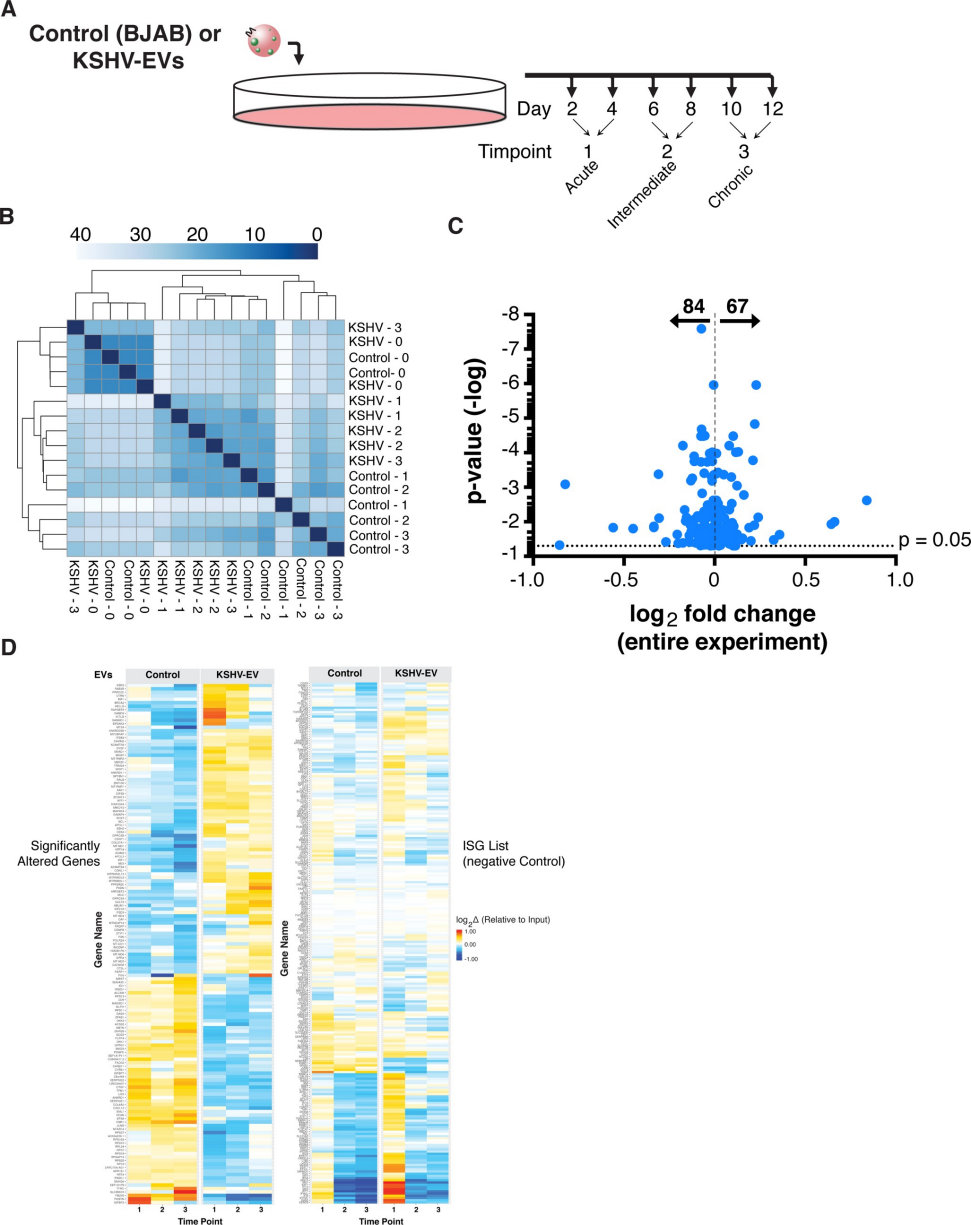
EV from BCBL1 PEL rewire recipient cell Gene expression. (A) Model for the treatment of hTERT-HUVECs with EV. Physiologically relevant concentrations of EV were continuously supplied to the endothelial cells (hTERT-HUVECs), which are targets of transformation by KSHV. RNA profile of the recipient cells was assayed by RNAseq at the indicated time points. Cells were treated with BJAB (control) EV or KSHV-EV. (B) Heatmap of the distances in a principal component analysis (PCA) of RNAseq reads from cells treated with PEL EV. hTERT-HUVECs were treated with Control EV or KSHV-EV over the course of 12 days (See also **S17 Fig**). KSHV-EV treated cells results had a different transcription profile compared to control EV-treated cell based on PERMANOVA test for significance of clustering (C) Fold change and p-value map of differentially expressed genes for the hTERT-HUVECs treated with KSHV-EV compared to control EV. P-values were not adjusted for multiple comparisons. (D) Time-dependent heatmap of RNAseq of genes. Significantly altered genes (left) and interferon stimulatory genes (ISGs) (right) were clustered onto heatmaps to show the time gene expression changes during acute, intermediate, and chronic exposure of EV. ISGs were shown as a negative control as they were largely unperturbed throughout treatment of both EV groups (see also **S18 Fig**).

In PEL and KS patients, KSHV-EV are continually released into the microenvironment and systemically into the blood and lymphatic circulation. Next to hemangioma, KS is the most angiogenic cancer in humans (reviewed in [3]). PEL grow as effusions, bathing the cavity walls in KSHV-EV. Thus, a physiological relevant experimental design would expose EC repeatedly to KSHV-EV. Such a design measures long-term cellular reprogramming (**Fig 9A**). Here, hTERT-HUVECs were exposed to KSHV-EV or BJAB-derived EV over a period of 12 days. Every 24 hours the media was replenished with fresh KSHV-EV or control-tumor EV, and cellular programming was analyzed by RNAseq. The time course was divided into an acute phase (day 2 and day 4), an intermediate phase (day 6 and day 8), and a chronic phase (day 10 and 12). KSHV-EV induced synchronized, progressive, and directional transcription profile changes compared to control (**Fig 9B,** see **S17 Fig** for a principal component analysis of hierarchical clustering). To identify continuous transcript changes over time, a likelihood ratio test was used. This identified 67 transcripts that were significantly upregulated and 84 that were significantly downregulated genes over the course of treatment (**Fig 9C,** and **S2 Table**). Heatmap representation of these altered genes show distinct contrasts between the treatment groups (**Fig 9D–**left), which were maintained over the 12 days of KSHV-EV exposure.

To test the hypothesis that KSHV-EV mediate any or all of the transcriptionally changes hitherto ascribed to KSHV miRNA expression within the infected cell, we analyzed transcriptional changes in genes that were previously identified in KSHV-infected EC [6, 28, 29]. Changes in these particular sets of mRNAs signal BEC to LEC transcription upon direct infection of KSHV. These mRNAs remained largely unchanged (**S18 Fig**). The notable exception was MAF1 mRNA, which was previously shown to be a direct target of multiple KSHV miRNAs with eleven predicted target sites in the 3’UTR [6], which was robustly down-regulated. As a control we analyzed a predefined set of interferon stimulatory genes (ISGs) [30] (**Fig 9D**, right). These showed induction during the acute phase for a few genes; however, these did not differ between control and KSHV-EV. This induction was not maintained over time, consistent with the idea that KSHV-EV reprogram EC towards a proliferative, activated phenotype, which is different from phenotypic changes induced by inflammation.

**Fig 10:**
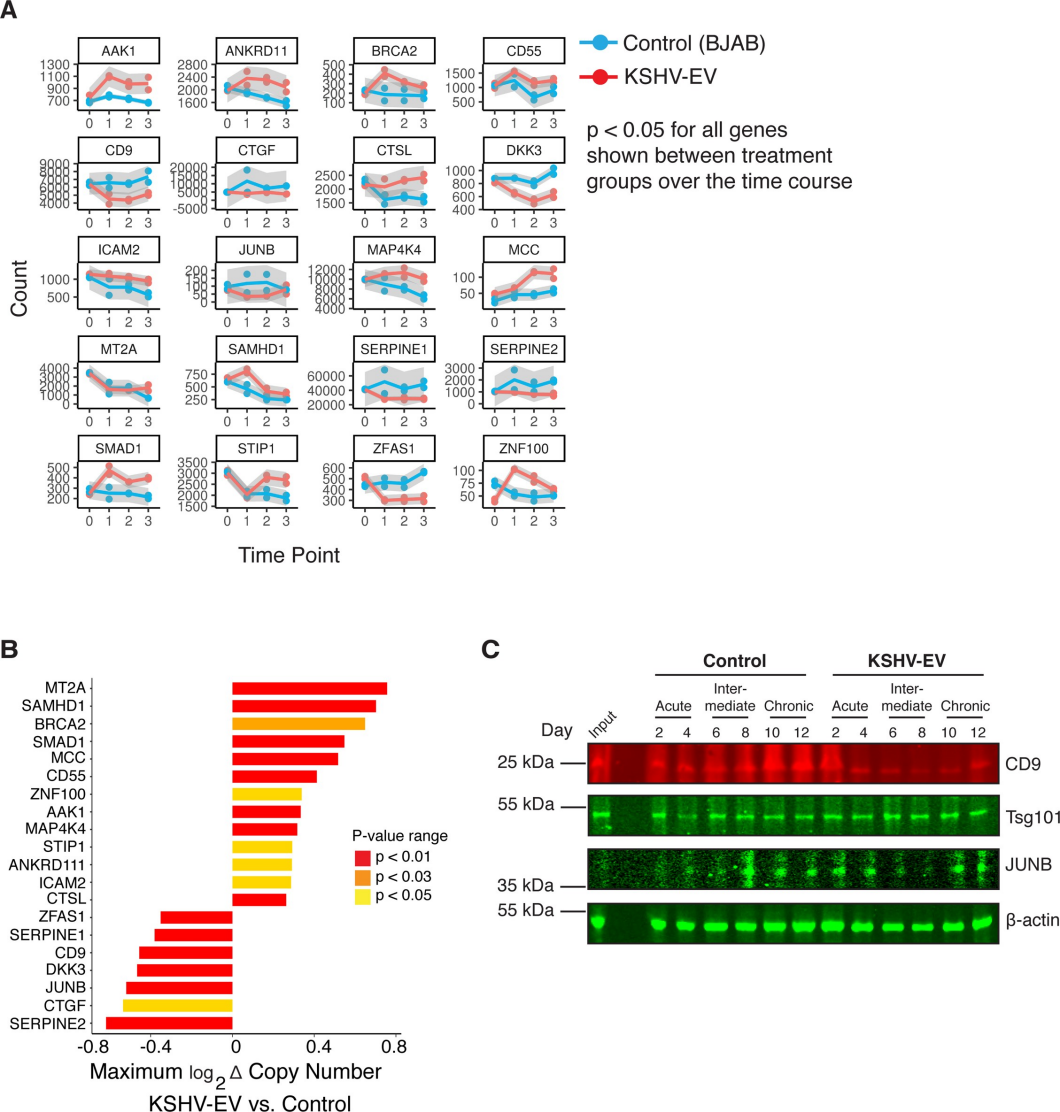
Analysis of significantly altered genes throughout the chronic KSHV-EV exposure. (A) Time dependent analysis of significantly altered genes. Time points of day 2 and 4 were combined for time point 1; day 6 and 8 were combined for time point 2; day 10 and 12 were combined for time point 3 (see methods). (B) Peak gene differential waterfall representation of 30 significantly altered genes between KSHV-EV and control EV treated hTERT-HUVECs. (C) Protein blot verification of RNAseq results. Protein pellets from the EV-treatment course were run to analyze protein levels of CD9 and JUNB, with Tsg101 and β-Actin serving as controls.

Time course analysis allowed for the identification of patterns of transcriptional changes for individual genes. The minimal set of EV-exposure biomarkers was comprised of genes with the highest and most consistent mRNA changes over time (**Fig 10A and 10B**). Among them were CD9 and JUNB, which we chose to validate at the level of protein expression (**Fig 10C**). CD9 protein levels were greatly reduced in KSHV-EV treated cells, particularly in the intermediate and chronic time points. As an internal control, we monitored the protein levels of a separate EV protein Tsg101, which remained constant in both treatment groups over time. JUNB levels were very low in the hTERT-HUVEC cells to begin with and further reduced at the intermediate time frame in the KSHV-EV treatment group. Thus, the transcriptional changes in response to KSHV-EV translated into protein level changes and physiological changes.

**Fig 11:**
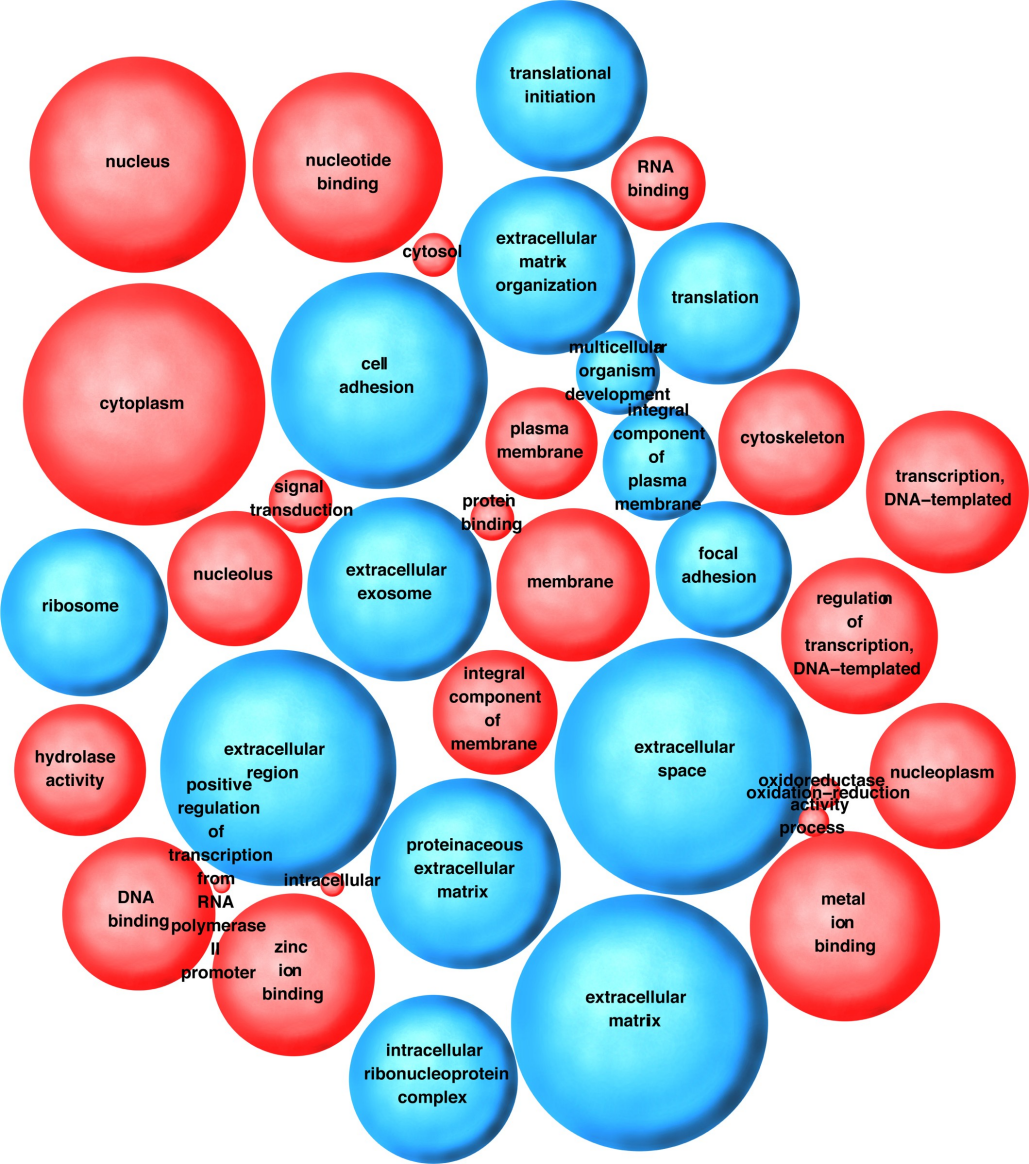
Bubble-plot representation of major pathways that were up- or down-regulated in the KSHV-EV exposure. (Altered genes were assigned to a major pathway and assayed for relative up- or downregulation. The color of the bubble indicates whether the pathway was upregulated (red) or downregulated (blue) and the size of the bubble indicates to the degree of regulation.

**Fig 12:**
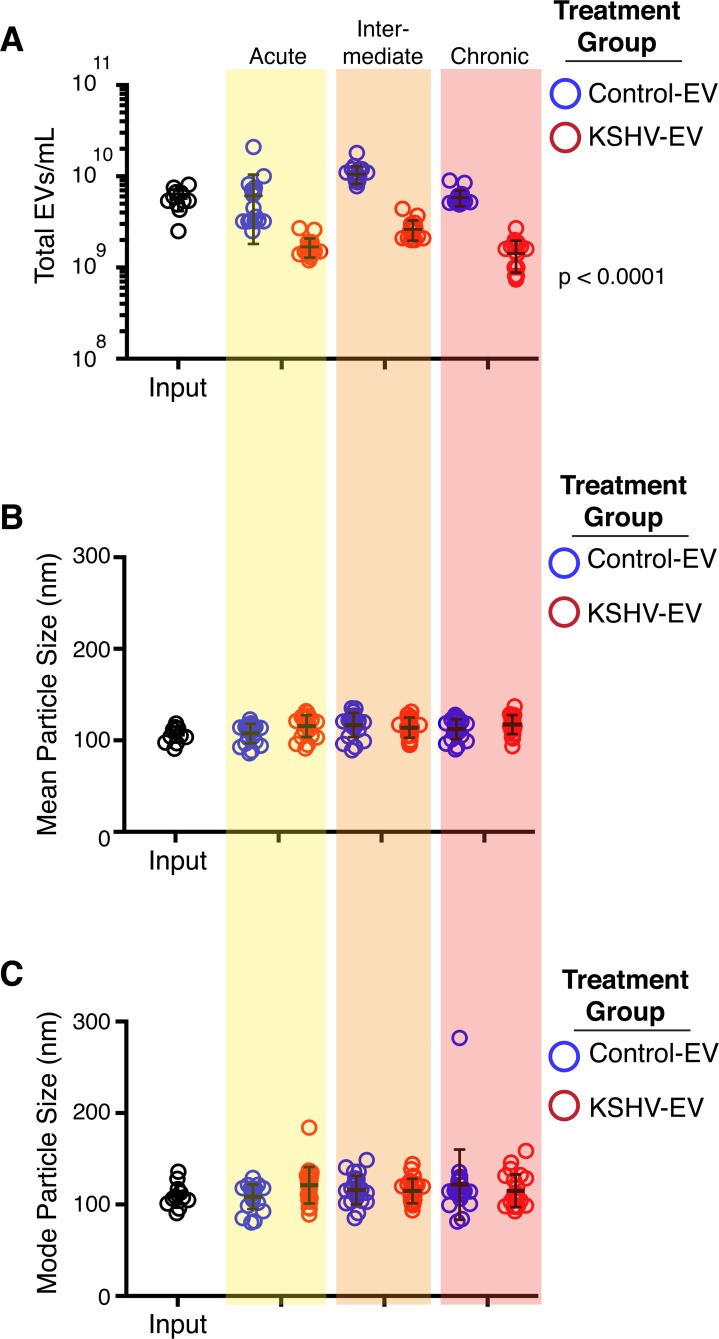
Treatment of endothelial cells with KSHV-EV reduces EV concentration. (A) EV released from hTERT-HUVECs treated with control (BJAB) or KSHV-EV were monitored for concentration through NTA. Acute, intermediate and chronic time points are shown in shaded areas. Groups were analyzed by ANOVA to determine statistical significance followed by Post-hoc tests for time points. (B) Mean particle size of the EV released from Control- or KSHV-EV treated cells. Statistical analyses done in (A) were performed and no significant differences were observed. (C) Mode particle size of the EV released from Control- or KSHV-EV treated cells. Statistical analyses done in (A) were performed and no significant differences were observed. (See also **S19 Fig**).

**Fig 13:**
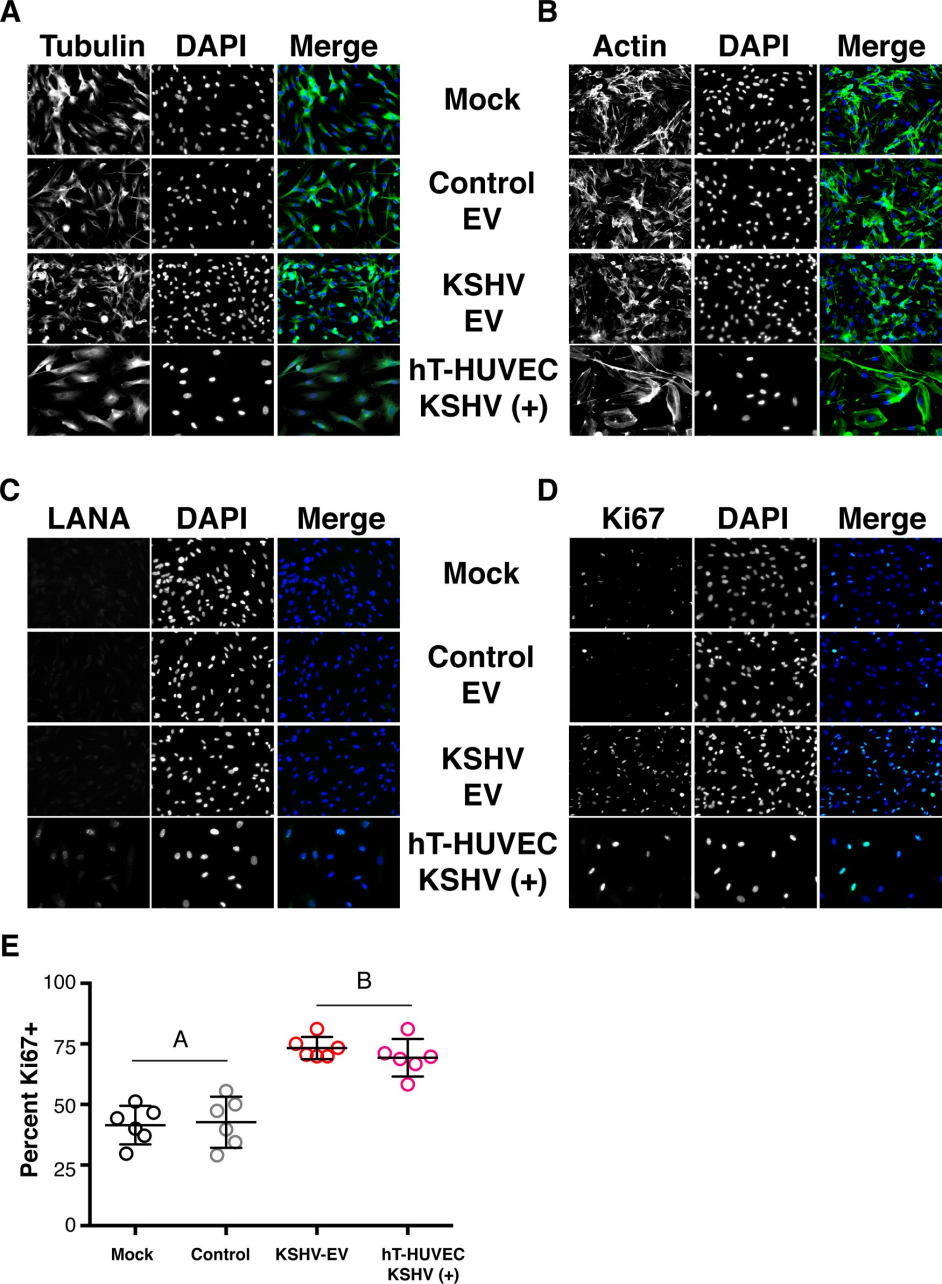
Treatment of endothelial cells with KSHV-EV enhances proliferation staining. (A) hTERT-HUVECs were treated with Mock, Control (BJAB)-EV, or KSHV-EV for 4 days and stained for Tubulin to monitor any changes in intracellular cytoskeleton. Mock-treated hTERT-HUVEC KSHV(+) served as a control. (B) hTERT-HUVECs were treated with Mock, Control (BJAB)-EV, or KSHV-EV for 4 days and stained for β-Actin to monitor any changes in intracellular cytoskeleton/plasma membrane manipulations. Mock-treated hTERT-HUVEC KSHV(+) served as a control. (C) hTERT-HUVECs were treated with Mock, Control (BJAB)-EV, or KSHV-EV for 4 days and stained for the infection marker LANA. Mock-treated hTERT-HUVEC KSHV(+) served as a control. (D) hTERT-HUVECs were treated with Mock, Control (BJAB)-EV, or KSHV-EV for 4 days and stained for the cell proliferation marker Ki-67 to monitor any changes in intracellular cytoskeleton. Mock treated hTERT-HUVEC KSHV(+) served as a control. (E) Percent positive Ki-67 cells were plotted and statistical analyses was done using ANOVA followed by T-tests (p≤0.05, n = 6; statistical groupings indicated with a letter) (See also **S20 Fig**).

The advantage of genome-wide transcriptional profiling lies in the identification of signaling networks, rather than individual genes. Hence, the significantly altered genes were clustered into gene ontology (GO) pathways [31]. The preeminent pathways identified herein related to extracellular matrix modulation, cell adhesion, growth and migration (**Fig 11**). Next, we explored phenotypic changes that would be consistent with the transcriptional pathways that dominated GO analysis. These are summarized in **S3 Table.** Experiments showing increased cell growth and migration (and thus decreased adhesion) in response to KSHV-EV, but not control-EV were already noted above. CD9 is a tetraspanin, which is involved in cell adhesion, motility and junctional integrity. It is also involved in EV biogenesis. To test whether the KSHV-EV-induced downregulation of CD9 at the mRNA and protein level led to a reduction in EV secretion in the recipient cells, we measured EV in the supernatant of hTERT-HUVEC cells at 24 hours after treatment with KSHV-EV. This experiment was possible because earlier studies had shown that exogenously added EV, analogous to liposomal transfection, are taken up within a few hours after addition to media [12, 32]. There were no changes in the biophysical characteristics of the hTERT-HUVEC-derived EV, but the total amount was reduced by comparison to control (**Fig 12** and **S18 Fig**). To test the hypothesis that KSHV-EV induce some, but not all phenotypes as KSHV infection, morphological differences in the recipient hTERT-HUVEC were evaluated. HTERT-HUVEC were exposed to KSHV-EV, mock, or BJAB-derived, control EV for four consecutive days. Chronically infected HUVECs served as a control. KSHV-EV treatment did not alter Tubulin (**Fig 13A**) or Actin (**Fig 13B**) organization. KSHV LANA was present in infected, but not EV-treated cells (**Fig 13C**). By contrast, the proliferation marker Ki-67 was dramatically induced by KSHV-EV, but not control EV. Ki-67 positivity was similar to KSHV infected cells (**Fig 13D and 13E**). H&E stain revealed a greater cell density in KSHV-EV compared to mock or BJAB-EV treated cells (**S20 Fig**). KSHV-infected hTERT cells exhibited greatly increased cell size, as previously described and consistent with mTOR/S6K activation [33]. Overall these results mirror the dramatic dysregulation of infected as well as uninfected EC in KS lesions, where the normal vasculature and extracellular environment is essentially destroyed and slit-like empty spaces develop. This analysis demonstrated that KSHV-EV inducing a long-lasting reprogramming of EC, which results in transcription signatures and pathway alterations consistent with the phenotypic changes observed in KS lesions.

## Discussion

KS is an incredibly angiogenic cancer, second only to hemangioma [3]. It is driven by KSHV-infected EC and defined by a unique molecular mechanism that manifests itself in aberrant EC behavior. Many studies have focused on the cell autonomous roles of KSHV [6, 28, 29, 33–38]. In addition, studies by Mesri and others (reviewed in [39]) have established that the KS phenotype depends to a large degree on paracrine signaling mechanisms to reprogram neighboring uninfected EC. This report establishes that KSHV-EV mediate some of the paracrine phenotypes of KS (summarized in **S3 Table**).

EV mediate a large variety of phenotypes in the immediate microenvironment as well as at distant sites. EV have established roles in cell differentiation, angiogenesis, cell migration as well as metastasis [40–44]. We had shown earlier that KSHV miRNAs are present in systemically circulating EV in KS patients, PEL fluid as well as in transgenic mice, which carry the KSHV miRNAs, but are not competent to make virions [7]. PEL are of post-GC lineage lymphoma, approaching almost plasmablastic stage. They grow i.p. (in body cavities), unlike Burkitt lymphoma, which are also post-GC lymphoma, but pre-plasmablastic and grow as a solid mass in lymph nodes, not as an effusion. This may explain the prominence that EV have in the biology of PEL and KSHV vis-a-vis other tumors.

Crucial to the study of EV is a well-validated purification pipeline [22, 45]. In the context of virus infections, it is important to exclude viral particles, which tend to co-purify with EV in ultracentrifugation, crowding-agent, and size exclusion chromatography approaches. Hence, we added affinity purification using antibody-coated beads directed against CD63 or other tetraspanins as the final purification step. This step depletes virions to below the limit of detection [7, 12], as it positively retains EV on a column rather than collecting a precipitate. It also reduces the complexity of the EV populations [10, 46] to only those EV that are of narrow size, tetraspanin-positive, and inhibited by Annexin-V. Adding an RNAse and DNAse step as well as size exclusion chromatography eliminated contaminating free RNA and DNA and selected against vesicles that are released non-specifically by dying cells. This has not always been done and may explain reports of EV preparations that induce heavy DNA and RNA-dependent immune stimulation in recipient cells. We believe the final product of our purification pipeline represents biologically-relevant KSHV-EV at or slightly below physiological concentration.

It has been a matter of debate as to whether EV induce or suppress the innate immune response. This phenotype depends largely on the specific virus and target cell. Professional immune cells, such as dendritic cells and macrophages are known to receive and transmit pro-inflammatory signals through EV [47–49]. RNA viruses induce a dramatic innate response and large amounts of secondary messengers, such as IFN-β or cGAMP [48]. DNA viruses, such as herpesviruses may also transmit pro-inflammatory signals through EV, which can be sensed by professional antigen presenting cells [47, 50]. While we cannot exclude that lytically replicating cells or professional antigen presenting cells infected with KSHV behave differently, these experiments demonstrate that EC, the primary target of KSHV infection, do not become activated by EV from KSHV-infected lymphoma cells or by EV from primary PEL fluid. They may become activated by cytokines or small soluble molecules, though a non-activated phenotype of uninfected cells would also be consistent with the biology of KSHV as most primary KSHV infections are clinically asymptomatic and not associated with mononucleosis-like symptoms or autoimmunity as seen with EBV or human cytomegalovirus infection.

These results support a model whereby cellular proteins, cellular and viral miRNAs that are carried in KSHV-EV modulate long-term reprogramming of EC in the immediate microenvironment of the tumor and systemically in the human host. Whereas prior studies focused on the immediate effects of a single bolus of EV, these experiments were designed to mimic continuous KSHV-EV exposure as seen in KS and PEL patients or patients with a high latent virus burden. The concentration of EV in normal blood is ~10^10^–10^11^/mL [23], which is ~ 6 orders of magnitude higher than the median concentration of KSHV in the blood of symptomatic, untreated AIDS-KS patients [51]. We added 10^10^/mL to 10^6^ cells (MOI = 10,000). Based on our prior work and **Fig 3**, we assume that all EV are endocytic-competent and all KSHV-EV carry the KSHV miRNAs. EV adsorption plateaus within hours of exposure [32]. Using a MOI bolus of 4000 vs. 1000 is unlikely to result in a qualitatively different response after 4 days. The novelty of this experimental design is mimicking chronic exposure, as is the case in the KS microenvironment or for any endothelial cell lining the blood vessels. Whereas PEL and KS, in the context of uncontrolled progression to AIDS, are rapidly fatal, *de novo* KSHV infection per se is not. HIV-negative endemic, pediatric and classic KS have rapid as well as smoldering clinical progression [52]. KSHV-associated multicentric Castleman’s disease has a waxing and waning presentation, closely associated with IL-6 levels [53]. IL-6, IL-10, VEGF, PDGF and other inflammatory cytokines are elevated in PEL, KS and MCD, and agents, such as pomalidomide, rapamycin and tocilizumab which modulate their levels, modulate disease [54–56]. KSHV-EV consistently induced human IL-6 in uninfected cells. These experiments show that in addition to cytokines EV also transmit pro-growth signals and can reprogram EC.

KSHV-EV induced a much more long-lasting phenotype than acute phase cytokines, which mimics differentiation and trans-differentiation. Whereas it was difficult to identify a single master regulator of this trans-differentiation phenotype, network analysis showed significant changes of transcriptional modules that regulate extracellular matrix remodeling, translation and exosome biogenesis. By comparison, IFN-β and NF-κB transcriptional networks were unaffected. KSHV-EV signaled through MAPK/ERK, which is consistent with MAPK/ERK’s role in modulating EC motility and vascular behavior (reviewed in [57]). Our observations are consistent with a recent study by Yogev *et al*. [58], who showed that KSHV-EV (derived from infected EC) induce metabolic remodeling of nearby uninfected cells. This represents perhaps the initiating step of trans-differentiation. Afterwards, continued KSHV-EV exposure resulted in continued reprogramming as has been described for KSHV-infected and KSHV-miRNA transfected EC [4–6, 34] and these experiments verified that most KSHV-miRNAs are present in KSHV-EV. Reprogramming here is used to defined an altered state of gene transcription and cell lineage, such as published by Hansen *et al*. [6], upon transfection of the KSHV miRNAs into EC, or upon infection of EC with KSHV [4, 28, 29, 59–61]. At this point we do not know if this reprogramming will persist after EV exposure has subsided and if not, how quickly the cells return to their normal state. Clinically, KS lesions and KS-associated edema regress as KSHV is cleared by immune restoration upon cART or lowering of immunosuppressive drugs in the context of transplant KS. Hence, we speculate that that the KSHV-EV induced phenotype likewise is transient. This would be in contrast to permanent lineage reprogramming, which is most commonly associated with epigenetic changes to the cellular DNA. Evidence for KSHV-infection induced chromatin remodeling has been published [62–65]. If in addition to transcriptional reprogramming, the viral miRNAs (or other molecules) that are contained in KSHV-EV also alter chromatin accessibility stably and irreversibly is a fascinating hypothesis and the subject of future studies.

The gradual and long-lasting reprogramming of transcriptional networks is consistent with the mechanism of action for miRNAs, which have their most physiological impact in development rather than acute signaling. EBV and KSHV express miRNAs and in infected cells these miRNAs account for as much as 50% of the miRNA pool. KSHV and EBV incorporate the viral miRNAs into EV [7, 66–68]. Both viruses substantially modulate the protein composition of EV [69]. In addition, EBV incorporates the LMP-1 oncogene into EV [70, 71], whereas no KSHV proteins were detected in EV thus far. This phenotype is consistent with the idea that EV are pivotal for establishing local tissue homeostasis and provides a molecular mechanism for it. Further studies are needed, but for the first time there now exists a highly reproducible, physiologically relevant experimental design to study long-term EV-EC interactions.

In conclusion, our findings point toward a novel means of cellular reprogramming by viruses. They pinpoint novel, actionable pathways for intervention and biomarker development. KSHV is able to infect EC, but the larger importance of EV stems from the fact that these vesicles can carry viral components to distant locations and transfer them into cells that the virus cannot enter. This may explain some of the phenotypes that viruses, including HIV, have on uninfected cells and it may explain why clinical sequalae persist long after the virus has been cleared or entered molecular latency.

## Materials and methods

### Cells and media

All cells were grown at 37°C in 5% CO_2_. hTERT-immortalized HUVECs were cultured in endothelial growth medium (EGM-2 media; Lonza) supplemented with the EGM-2 Bulletkit (Lonza) and 10% EV-depleted fetal bovine serum (FBS). BCBL1 (from the laboratory of Dr. D. Gamen) cells were cultured in RPMI 1640 (Gibco) supplemented with 10% Tetracycline-free, EV-depleted FBS (Clontech), 100 U/mL penicillin G (Gibco), 100 μg/mL streptomycin sulfate (Gibco) and 2 mM L-glutamine (Gibco). BJAB (from the laboratory of Dr. D. Gamen) cells were cultured in RPMI 1640 supplemented with 10% EV-depleted FBS, 100 U/mL penicillin, 100 μg/mL streptomycin sulfate, and 2 mM L-glutamine. Namalwa (EBV-positive, obtained from the ATCC #CRL-1432) were grown in the same conditions as BCBL1 cells.

### EV isolation

Total EV were isolated using approximately 400 mL of cell culture supernatant. Cells were pelleted at 4˚C at 800x g for 10 minutes. Supernatant was then passed through a 0.22 μm Nalgene Rapid Flow Filter (Thermo Fisher). Filtered supernatants were aliquoted into individual 50 mL conical tubes (Corning). EV were precipitated with 40 mg/mL PEG-8000 and incubation at 4˚C for >8 hours. Precipitates were then spun down at 4˚C at 1,200x g for 60 minutes. Pellets were resuspended in 500 μL of ice-cold 1X PBS (Gibco). Removal of non-associated molecules were done by (i) ultracentrifugation or (ii) column chromatography. (i) For ultracentrifugation, the volume was increased to ~4 mL with 1X PBS and centrifuged at 4˚C at 120,000x g for 60 minutes using a Beckmann SW32 rotor. The pellet was then resuspended in 4 mL of fresh 1X PBS and centrifuged again. A total number of three washes was done. The final pellet was resuspended in 100 μL of fresh, ice-cold 1X PBS. (ii) For column chromatography, GE Sephadex G-200 was equilibrated with ice-cold 1X PBS for a total of 4 compacted bead volumes (4 mL). The resuspended EV were added to the equilibrated column and allowed to flow through the column by gravity. EV were collected in the first 1 mL of fresh, cold 1X PBS. EV were also isolated from isolated from 50 mL plasma from health donors or 10 mL PEL. Briefly, blood was processed and erythrocytes, leukocytes, platelets, and plasma were separated using Ficoll reagent (GE 17-1440-02) as above.

### EV purification using CD63, CD9, and CD81 Dynabeads

Samples were enriched for CD63, CD9, and CD81 positive EV using magnetic beads (ThermoFisher 1060D, 10620D, and 10622D, respectively). Briefly, the total EV isolated as above were added to 80 μL of equilibrated, antibody-coated magnetic beads. Non-specific IgG-coated beads were used as a control. EV were bound to beads overnight at 4˚C and beads were washed 3X with 1X PBS. EV were eluted in 100 μL of elution buffer (Invitrogen) or 0.2 M Glycine pH = 2.0 for further analysis. Cell were authenticated by targeted amplification of STR typing loci using Ion Torrent Precision ID GlobalFiler NGS STR Panel and compared against the STR database of the German Collection of Microorganisms and Cell Cultures GmbH.

### Flow cytometry

Total EV were labeled with 1 μM Dil (1,1’-dioctadecyl-3,3,3’3’-tetramethylindocarbocyanine perchlorate; Sigma), 0.5X ExoGreen (SystemBio), followed by G25 column filtration and incubated with 40 μL CD63, CD9, or CD81 beads, washed 3x with PBS and analyzed using the BD Accuri C6 Plus flow cytometer (BD Biosciences). FITC and PE settings were used to detect ExoGreen and Dil with an excitation laser of 488 nm and emission filters of 533 nm and 585 nm, respectively. Unlabeled EV were used to set background fluorescence. Results were analyzed using FloJo 2.0.

### Fluorescence and Immunofluorescence microscopy

hTERT-HUVECs were grown on a cover slip in 6 well plates in a total volume of 3 mL and treated with labeled 10^9^ EV/mL for the indicated time period at 37°C. Cells were then rinsed with PBS and fixed in 4% paraformaldehyde for 10 minutes at RT, washed 3 times with PBS and the cells permeabilized using 0.5% Triton X-100 in PBS for 10 minutes and washed 3x with PBS. For indirect immunofluorescence, coverslips were blocked in a solution of 10% goat serum (Vector Labs) in PBS with 0.2% Triton X-100 and incubated with primary antibodies: anti-IRF3 antibody (Cell Signaling, #4962, 1:100 dilution) anti-P-NF-κB p65 (S536) (clone 93H1, Cell Signaling, 1:100 dilution). Coverslips were washed three times with PBS-0.2% Triton with 2% BSA and incubated with FITC-conjugated anti-rabbit secondary antibody (#FI-1000, Vector Labs Inc. 1:500 dilution). Cells were washed three times with PBS-0.2% Triton X-100 with 2% BSA and stained with 0.2 μg/mL DAPI (Sigma) prior to mounting in VectaShield (Vector Labs). Cells were imaged on a Leica DM4000B microscope with a Q-Imaging Retiga-2000RV camera and HCX-PL-APO 506187 lens at 63x magnification. De-convoluted images (Simple PCI 6 software Metamorph v 7.8.12.0, 10 iterations RB, GB or RGB) were then opened in Imaris V 9.2.0. and background subtraction of all channels was done using recommended settings of 400 um filter width. Localizations of EV-delivered Dil and ExoGreen were done using the “Add Spots” command using spots of different sizes depending on fluorescence intensity. Regions of spot calling were standardized to linear detection ranges using absolute intensity. For nuclei staining, the “Add New Surfaces” command was used.

### WNV infection and Poly I:C treatment

As a positive control for IRF3 activation, hTERT-HUVECs were infected with West Nile Virus NY99 (WNV) at a MOI of 5 (observed after 36 hours) or Poly I:C at 5 μg/mL (observed after 12 hours).

### Western Blotting and Silver staining

Pellets (10^10^ EV or 10^6^ cells) were lysed in 100 μL NP-40 lysis buffer and run on an 8% SDS-PAGE gel, transferred to a nitrocellulose membrane (Hybond) and blocked in 5% dry milk in TBS overnight at 4°C. Antibodies are listed in **S1 Table**. For detection of tetraspanins, non-reducing conditions were used. To visualize total protein by silver stain, we used the Pierce Silver Stain Kit (ThermoFisher) after which bands were excised and analyzed by mass spectrometry at the UT Southwester core (https://www.utsouthwestern.edu/research/core-facilities/proteomics-core.html).

### Annexin-V, heparin, soluble IL-6 receptor and MEK inhibitor treatment

To block EV entry, EV were incubated with recombinant Annexin-V (2 μg/mL, BD Biosciences) for 30 minutes prior to addition to hTERT-HUVECs. To block virus entry EV were incubated with 50 μg/mL heparin (Lonza). To block autocrine IL-6 feedback, 10 ng/mL IL-6 receptor (sIL-6R, Peprotech #200-06R) was added to cells 24 hours before EV addition. To inhibit MEK and ERK1/2, AZD6244 (SelleckChem) and PD184352 (SelleckChem), respectively were used to treat cells 24 hours prior to addition EV addition at indicated concentrations.

### Cell proliferation and migration

(i) Scratch assays were performed as previously described [7]. Briefly, hTERT-HUVECs were grown in a 24-well plate (Corning) prior to treatment with EV. The wound was initiated using a standard 200 μL pipette tip and the cells were then washed and replaced with fresh media containing one of the following as a chemo-attractant: 10% FBS, 10 ng/mL VEGF (Peprotech), 1 U/mL hIL-6 (Peprotech), 10 ng/mL PDGF-β (Peprotech) or 10 ng/mL SDF-1α (CXCL12) (R&D Systems). The culture was monitored over time. Images were obtained using a Leica DMIL microscope with a HI Plan 10x/0.25 PHI objective and QImaging camera (Cooled color, RTV 10 bit) paired with QCapture imaging software 3.0. Images are shown at 100x magnification and were analyzed using ImageJ software to calculate the percent wound closure at a given time point. (ii) Cell proliferation and migration was analyzed using the xCelligence RTCA DP instrument as previously described [7]. Briefly, hTERT-HUVECs were treated with EV for 24 hours and proliferation measured by conductance. For migration, both sides of the xCelligence CIM Plate 16 (Acea Biosciences) plate membrane were coated with 20 μg/mL fibronectin prior to assembly and media containing FBS or a specified cytokine was placed in the lower chamber as the chemo-attractant. Cells were plated at 15,000 cells per well of the upper chamber. Reads were taken every 2 minutes for a period of 12 hours. The cell index reflects the degree of cellular migration towards the specified chemo-attractant.

### ELISA

Levels of IL-6, IL-8, IL-10, IL-18 and IL-1β were determined by ELISA according to the manufacturer’s protocol (eBioscience, #88–7066 (IL-6), #88–8086 (IL-8), #88–7106 (IL-10), #BMS267INST (IL-18) and #88–7010 (IL-1β). The average of at least three wells is reported for each biological replicate.

### Gene expression profiling

Total RNA was isolated using TRI reagent (Molecular Research Center) as previously described (https://www.med.unc.edu/vironomics/services/protocols/), treated with Turbo DNA-free kit (Ambion, Life Technologies) and 100 ng of DNA-free RNA, as determined by Nanodrop, was used as input for High Capacity cDNA synthesis kit (Applied Biosystems, Life Technologies). Custom NF-κB and endothelial lineage real-time qPCR arrays were used previously published [72].

### Electron microscopy

EV were adsorbed on a glow-charged carbon coated 400-mesh copper grids for 2 minutes and then stained with 2% (weight/volume) uranyl acetate in water. Transmission electron microscopy (TEM) images were taken using a Philips CM12 electron microscope at 80 kilovolts. Images were captured on a Gatan Orius camera (2000 x 2000 pixels) using the Digital Micrograph software (Gatan, Pleasanton, CA). Images were then cropped in Adobe Photoshop.

### Statistics and Bioinformatics

(i) For continuous, variable pairwise T-tests were performed to determine statistical significance for n ≥ 3 biological replicates. (ii) For comparison of mass spectrometry data, a hypergeometric test was used. (iii) For analysis of RNAseq data we used a custom pipeline. The decision to use our particular analysis is discussed at length at https://support.bioconductor.org/p/62684/. In brief, STAR-Aligned BAM files representing table of counts for each samples were processed using DESeq and other Bioconductor packages: https://bioconductor.org/packages/devel/bioc/vignettes/GenomicAlignments/inst/doc/summarizeOverlaps.pdf.

## Supporting information

S1 Fig EV Biophysical analysis from B-cell lymphomas. (A) Size distribution analysis post-PEG precipitation of EV taken from various B-cell lymphomas. BJAB (Burkitt lymphoma, non-infected), BCBL1 (Primary Effusion Lymphoma, KSHV-infected), Namalwa (Burkitt lymphoma, EBV-infected).
(B) Total EV particles per mL of supernatant from the B-cell lymphomas.
(C) Mean (open circles) and mode sizes of the EV particles from the B-cell lymphomas.
(TIF)

S2 Fig Mass spectrometry analysis of column filtrated total EV from BJAB and BCBL1 cells. Filtrated EV were run out on a denaturing polyacrylamide gel and gel slices were analyzed by mass spectrometry. Top high-confidence hits were analyzed against known exosome constituents in the ExoCarta database. Statistical analysis for overlay was done using the hypergeometric test.
(TIF)

S3 Fig Analysis of purified post-PEG EV using ultracentrifugation. (A) Size distribution analysis post-ultracentrifugation was done using the PEG-precipitated EV from BJAB and BCBL1 cells. Expected size ranges of exosomes and microvesicles are shown.
(B) Mean (open circle) and mode (gray square) sizes of the ultracentrifuged EV from the PEG-precipitate.
(C) Total EV particles per mL of supernatant from BJAB (solid blue) or BCBL1 (dashed red) cells from the post-ultracentrifugation, PEG precipitate.
(D) Relative acetylcholine esterase (AchE) activity of the post-ultracentrifuged, PEG-precipitated EV. Substrate only is shown for reference against BJAB (solid blue) and BCBL1 (dashed red) EV.
(E) Silver stain analysis of the post-ultracentrifuged, PEG precipitated EV from BJAB and BCBL1. PEG-precipitated cell culture media was used as a control for background.
(TIF)

S4 Fig Analysis of EV purified post-PEG precipitation using column filtration. (A) Size distribution analysis post-column filtration was done using the PEG-precipitated EV from BJAB and BCBL1 cells. Expected size ranges of exosomes and microvesicles are shown.
(B) Mean (open circle) and mode (gray square) sizes of the column filtrated EV from the PEG-precipitate.
(C) Total EV particles per mL of supernatant from BJAB (solid blue) or BCBL1 (dashed red) cells from the post-column filtrated, PEG precipitate.
(D) Relative acetylcholine esterase (AchE) activity of the post-column filtrated, PEG-precipitated EV. Substrate only is shown for reference against BJAB (solid blue) and BCBL1 (dashed red) EV.
(E) Silver stain analysis of the post-ultracentrifuged, PEG precipitated EV from BJAB and BCBL1. PEG-precipitated cell culture media was used as a control for background.
(TIF)

S5 Fig Analysis of EV from healthy donors or primary effusion lymphoma purified post-PEG precipitation using column filtration. (A) Size distribution analysis post-column filtration was done using the PEG-precipitated EV from healthy donors and primary effusion lymphoma (PEL). Expected size ranges of exosomes and microvesicles are shown.
(B) Mean (open circle) and mode (gray square) sizes of the column filtrated EV from the PEG-precipitate.
(C) Total EV particles per mL of supernatant from the healthy donors and the PEL samples from the post-column filtrated, PEG precipitate.
(D) Relative acetylcholine esterase (AchE) activity of the post-column filtrated, PEG-precipitated EV. Substrate only is shown for reference against healthy donors and PEL EV.
(TIF)

S6 Fig Affinity purification of EV from the total EV fraction. (A) EV were affinity captured using anti-CD63 magnetic beads and products were run out for protein and nucleic acid analysis. CD63, CD81, CD9, and Flotillin-2 were used to monitor the successful immunoprecipitation.
(B) miRK12-5 was reverse transcribed from the fractions and amplified by qRT-PCR. Products were run on the Caliper LabChip GX.
(C) KSHV DNA genomes were quantified from each fraction via qPCR.
(D) Size distribution analysis post-affinity capture was done using the BJAB, BCBL1, HD, PEL EV. Expected size ranges of exosomes and microvesicles are shown.
(C) Mean (open circle) and mode (gray square) sizes of the affinity captured EV from the PEG-precipitate.
(D) EV particles per mL of supernatant from the healthy donors and the PEL samples from the post-column filtrated, PEG precipitate.
(E) Negative stain electron micrographs of affinity captured EV from HD.
(F) Negative stain electron micrographs of affinity captured EV from PEL.
(TIF)

S7 Fig Labeling of CD63+ affinity-captured EV. (A) Scheme for labeling of affinity purified EV. EV were purified using antibodies directed to the tetraspanins presented on the surface of EV (CD63, CD9, and CD81). The lipid dye Dil will fluorescently label the EV red and the AchE reporter ExoGreen will fluorescently label internal proteins green.
(B) The affinity capture-negative control (PBS) without any label was conjugated to anti-CD63 beads and run for flow cytometry analysis.
(C) The affinity capture-negative control (PBS) was incubated with Dil and conjugated to anti-CD63 beads and run for flow cytometry analysis.
(D) The affinity capture-negative control (PBS) was incubated with ExoGreen and conjugated to anti-CD63 beads and run for flow cytometry analysis.
(E) The affinity capture-negative control (PBS) was incubated with both Dil and ExoGreen and conjugated to anti-CD63 beads and run for flow cytometry analysis.
(F) The affinity capture of HD EV without any label was conjugated to anti-CD63 beads and run for flow cytometry analysis.
(G) The affinity capture of HD EV was incubated with Dil and conjugated to anti-CD63 beads and run for flow cytometry analysis.
(H) The affinity capture of HD EV was incubated with ExoGreen and conjugated to anti-CD63 beads and run for flow cytometry analysis.
(I) The affinity capture of HD EV was incubated with both Dil and ExoGreen and conjugated to anti-CD63 beads and run for flow cytometry analysis.
(J) The affinity capture of PEL EV without any label was conjugated to anti-CD63 beads and run for flow cytometry analysis.
(K) The affinity capture of PEL EV was incubated with Dil and conjugated to anti-CD63 beads and run for flow cytometry analysis.
(L) The affinity capture of PEL EV was incubated with ExoGreen and conjugated to anti-CD63 beads and run for flow cytometry analysis.
(M) The affinity capture of PEL EV was incubated with both Dil and ExoGreen and conjugated to anti-CD63 beads and run for flow cytometry analysis.
(TIF)

S8 Fig Labeling of CD9+ affinity-captured EV. (A) The affinity capture-negative control (PBS) without any label was conjugated to anti-CD9 beads and run for flow cytometry analysis.
(B) The affinity capture-negative control (PBS) was incubated with Dil and conjugated to anti-CD9 beads and run for flow cytometry analysis.
(C) The affinity capture-negative control (PBS) was incubated with ExoGreen and conjugated to anti-CD9 beads and run for flow cytometry analysis.
(D) The affinity capture-negative control (PBS) was incubated with both Dil and ExoGreen and conjugated to anti-CD9 beads and run for flow cytometry analysis.
(E) The affinity capture of HD EV without any label was conjugated to anti-CD9 beads and run for flow cytometry analysis.
(F) The affinity capture of HD EV was incubated with Dil and conjugated to anti-CD9 beads and run for flow cytometry analysis.
(G) The affinity capture of HD EV was incubated with ExoGreen and conjugated to anti-CD9 beads and run for flow cytometry analysis.
(H) The affinity capture of HD EV was incubated with both Dil and ExoGreen and conjugated to anti-CD9 beads and run for flow cytometry analysis.
(I) The affinity capture of PEL EV without any label was conjugated to anti-CD9 beads and run for flow cytometry analysis.
(J) The affinity capture of PEL EV was incubated with Dil and conjugated to anti-CD9 beads and run for flow cytometry analysis.
(K) The affinity capture of PEL EV was incubated with ExoGreen and conjugated to anti-CD9 beads and run for flow cytometry analysis.
(L) The affinity capture of PEL EV was incubated with both Dil and ExoGreen and conjugated to anti-CD9 beads and run for flow cytometry analysis.
(TIF)

S9 Fig Labeling of CD81+ affinity-captured EV. (A) The affinity capture-negative control (PBS) without any label was conjugated to anti-CD81 beads and run for flow cytometry analysis.
(B) The affinity capture-negative control (PBS) was incubated with Dil and conjugated to anti-CD81 beads and run for flow cytometry analysis.
(C) The affinity capture-negative control (PBS) was incubated with ExoGreen and conjugated to anti-CD81 beads and run for flow cytometry analysis.
(D) The affinity capture-negative control (PBS) was incubated with both Dil and ExoGreen and conjugated to anti-CD81 beads and run for flow cytometry analysis.
(E) The affinity capture of HD EV without any label was conjugated to anti-CD81 beads and run for flow cytometry analysis.
(F) The affinity capture of HD EV was incubated with Dil and conjugated to anti-CD81 beads and run for flow cytometry analysis.
(G) The affinity capture of HD EV was incubated with ExoGreen and conjugated to anti-CD81 beads and run for flow cytometry analysis.
(H) The affinity capture of HD EV was incubated with both Dil and ExoGreen and conjugated to anti-CD81 beads and run for flow cytometry analysis.
(I) The affinity capture of PEL EV without any label was conjugated to anti-CD81 beads and run for flow cytometry analysis.
(J) The affinity capture of PEL EV was incubated with Dil and conjugated to anti-CD81 beads and run for flow cytometry analysis.
(K) The affinity capture of PEL EV was incubated with ExoGreen and conjugated to anti-CD81 beads and run for flow cytometry analysis.
(L) The affinity capture of PEL EV was incubated with both Dil and ExoGreen and conjugated to anti-CD81 beads and run for flow cytometry analysis.
(TIF)

S10 Fig Prolonged treatment of cells with labeled EV results in dispersement of the lipid dye. CD63-captured EV from BJAB, BCBL1 cultured cells, or from HD or primary PEL were added to hTERT-HUVEC for 24 hours and cells were assayed for uptake by fluorescence microcopy. 3-D images were taken and deconvoluted. The lipid dye Dil showed a more dispersed fluorescence pattern, whereas the protein dye ExoGreen remained in punctate structures.
(TIF)

S11 Fig ExoDot representation of cells treated with dual-labeled EV after 24 hour exposure. (A) 3-D dot representation of hTERT-HUVECs treated with dual labeled EV taken from BJAB cells.
(B) 3-D dot representation of hTERT-HUVECs treated with dual labeled EV taken from BCBL1 cells.
(C) 3-D dot representation of hTERT-HUVECs treated with dual labeled EV taken from HD plasma.
(D) 3-D dot representation of hTERT-HUVECs treated with dual labeled EV taken from primary PEL fluid.
(TIF)

S12 Fig PEL EV accelerates cell migration in the presence of the cytokines. (A) hTERT-HUVECs were plated in a specialized xCelligence CIM-plate in the presence of VEGF. In the chamber below the cells were HD EV, KSHV-EV, or primary PEL EV.
(B) Same as for (A), but with IL-6.
(C) Same as for (A), but with PDGF-β.
(D) Same as for (A), but for SDF1-α.
(E) Endpoint analysis of the relative migration of hTERT-HUVECs in the presence of VEGF toward the bottom chamber containing HD EV, KSHV-EV, or primary PEL EV (asterisks indicates that HD was significantly different from KSHV-EV and primary PEL EV treated samples).
(F) Endpoint analysis of the relative migration of hTERT-HUVECs in the presence of IL-6 toward the bottom chamber containing HD EV, KSHV-EV, or primary PEL EV (asterisks indicates that HD was significantly different from KSHV-EV and primary PEL EV treated samples).
(G) Endpoint analysis of the relative migration of hTERT-HUVECs in the presence of PEGF-β toward the bottom chamber containing HD EV, KSHV-EV, or primary PEL EV.
(H) Endpoint analysis of the relative migration of hTERT-HUVECs in the presence of SDF1-α toward the bottom chamber containing HD EV, KSHV-EV, or primary PEL EV.
(TIF)

S13 Fig Quantitation of cytokine secretion by hTERT-HUVECs in response to EV treatment. (A) hTERT-HUVECs were incubated with HD EV, KSHV-EV, or primary PEL EV and supernatant was assayed for the presence of the cytokine IL-10. ELISA determined amounts of the cytokine. As a positive control, cells were transfected with the double-stranded RNA mimic Poly I:C.
(B) hTERT-HUVECs were incubated with HD EV, KSHV-EV, or primary PEL EV and supernatant was assayed for the presence of the cytokine IL-18. ELISA determined amounts of the cytokine. As a positive control, cells were transfected with the double-stranded RNA mimic Poly I:C.
(C) hTERT-HUVECs were incubated with HD EV, KSHV-EV, or primary PEL EV and supernatant was assayed for the presence of the cytokine IL-1β. ELISA determined amounts of the cytokine. As a positive control, cells were transfected with the double-stranded RNA mimic Poly I:C.
(D) hTERT-HUVECs were incubated with HD EV, KSHV-EV, or primary PEL EV and supernatant was assayed for the presence of the cytokine INF-α. ELISA determined amounts of the cytokine. As a positive control, cells were transfected with the double-stranded RNA mimic Poly I:C.
(TIF)

S14 Fig KSHV/PEL EV do not induce translocation of the p65 subunit of NF-κB. hTERT-HUVECs were incubated with HD EV, KSHV-EV, or primary PEL EV and assayed for induced translocation of the p65 subunit of NF-κB. As a positive control, cells were transfected with Poly I:C.
(TIF)

S15 Fig KSHV/PEL EV do not induce NF-κB-dependent gene expression. Array of 90 known NF-κB regulated genes were monitored for activation by HD EV, KSHV-EV, or primary PEL EV at 6 and 24 hours post EV treatment. Fold change of the indicated gene is shown in a heatmap.
(TIF)

S16 Fig Inhibition of the MEK/ERK signaling pathway inhibits EV-dependent cell migration. hTERT-HUVECs seeded in a specialized xCelligence CIM plate in the presence of DMSO or PD184352 (1 μM in DMSO). Cells were pre-treated with the inhibitor and subsequently exposed to control- or KSHV-EV. Cell index was monitored over the course of 24 hours. Shaded areas represent S.D.
(TIF)

S17 Fig PCA Plot Representation of RNAseq profile of cells treated with EV during the time course. PCA plot of RNAseq reads from cells treated with BJAB (control) EV or KSHV-EV. hTERT-HUVECs were treated with Control EV or KSHV-EV over the course of 12 days. Ellipsoids represent clustering of the treatment groups (p < 0.05).
(TIF)

S18 Fig KSHV-EV does not alter genes directly influenced by virus infection or KSHV miRNA-expression alone. Heatmap of mRNAs that are significantly altered during KSHV infection of endothelial cells or by the viral miRNA as previously reported [6, 28, 29]. Shown is a comparison of control and KSHV-EV treated cells. Of the genes identified, only 3 reached statistical significance, which contain a * next their gene name.
(TIF)

S19 Fig KSHV-chronically infected endothelial cells release fewer EV than non-infected cells. (A) Size distribution profile of EV isolated from hTERT-HUVECs or hTERT-HUVECs chronically infected with KSHV (hTERT-HUVEC KSHV(+)).
(B) Mean and Mode sizes of EV isolated from hTERT-HUVECs or hTERT-HUVEC KSHV (+).
(C) Concentration of EV released from hTERT-HUVECs or hTERT-HUVECS KSHV (+). Significant differences were determined by T-test.
(TIF)

S20 Fig Treatment with KSHV-EV do not change morphological features of ECs. (A) hTERT-HUVEC were stained with Hematoxylin and eosin (H and E) after four days of continuous mock treatment. Objective = 40X.
(B) hTERT-HUVECs were stained with H and E after four continuous days of Control-(BJAB) EV treatment. Objective = 40X.
(C) hTERT-HUVECs were stained with H and E after four continuous days of KSHV-EV treatment. Objective = 40X.
(D) hTERT-HUVEC KSHV (+) were stained with H and E as a control. Objective = 40X.
(TIF)

S1 Table List of antibodies used in this study, as well as their manufacturer and catalog numbers. (DOCX)

S2 Table List of high-confidence, significantly altered genes in the KSHV-EV treatment group. All genes listed are significantly different from both Input and the Control-EV treatment groups all time points.
(DOCX)

S3 Table Summary of functional assays performed with KSHV-EV and how the resulting phenotype compared with Control-EV (either BJAB-EV or HD-EV). (DOCX)
