# Supplementary figures and images for: Extracellular vesicles from Kaposi Sarcoma-associated herpesvirus lymphoma induce long-term endothelial cell reprogramming
Source: PLoS Pathog. 2019 Feb 4;15(2):e1007536. doi: 10.1371/journal.ppat.1007536 (PMC6361468; doi:10.1371/journal.ppat.1007536)

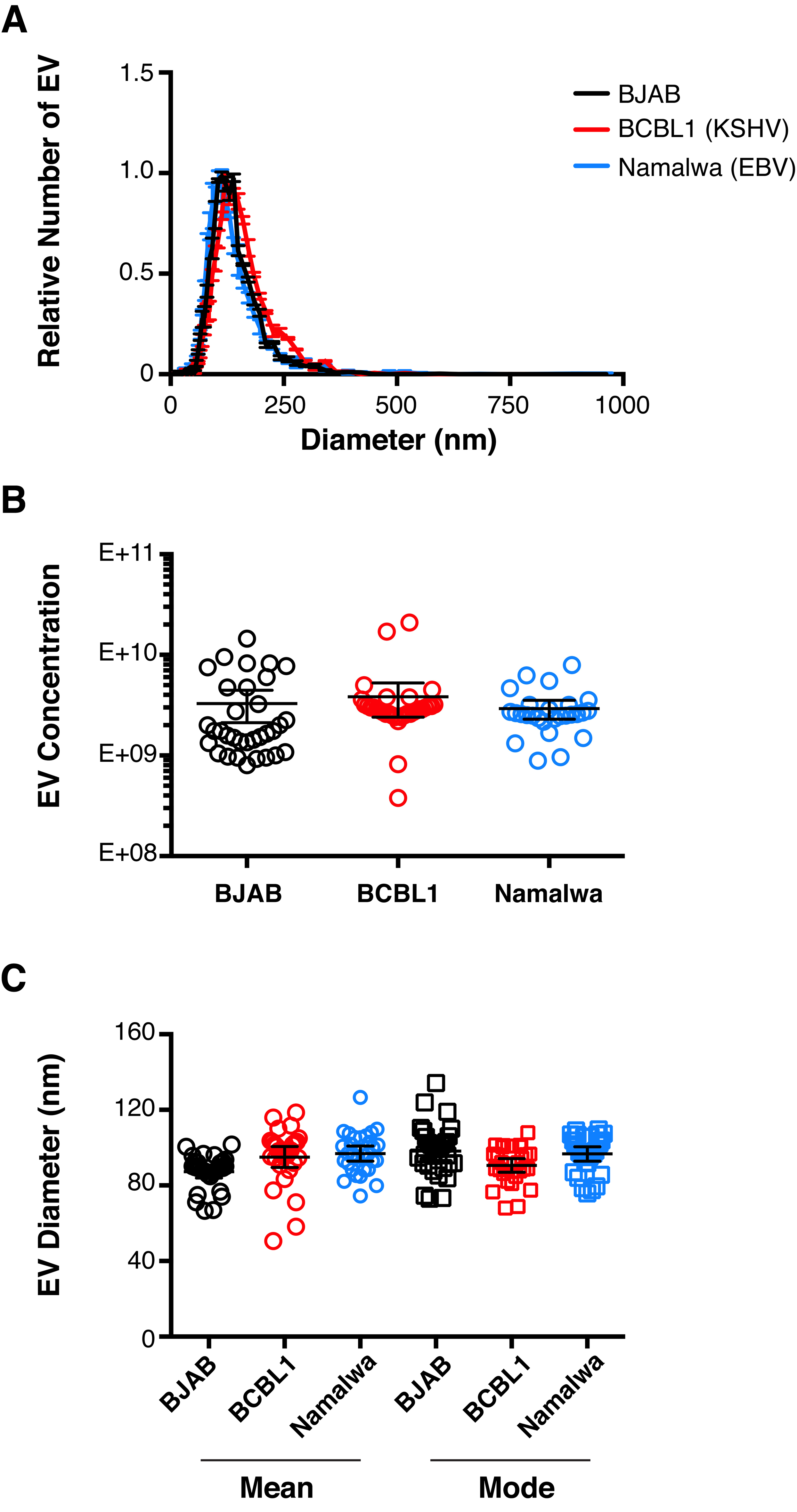

Supplement: S1 Fig — (A) Size distribution analysis post-PEG precipitation of EV taken from various B-cell lymphomas. BJAB (Burkitt lymphoma, non-infected), BCBL1 (Primary Effusion Lymphoma, KSHV-infected), Namalwa (Burkitt lymphoma, EBV-infected). (B) Total EV particles per mL of supernatant from the B-cell lymphomas. (C) Mean (open circles) and mode sizes of the EV particles from the B-cell lymphomas. (TIF) [file ppat.1007536.s001.tif]

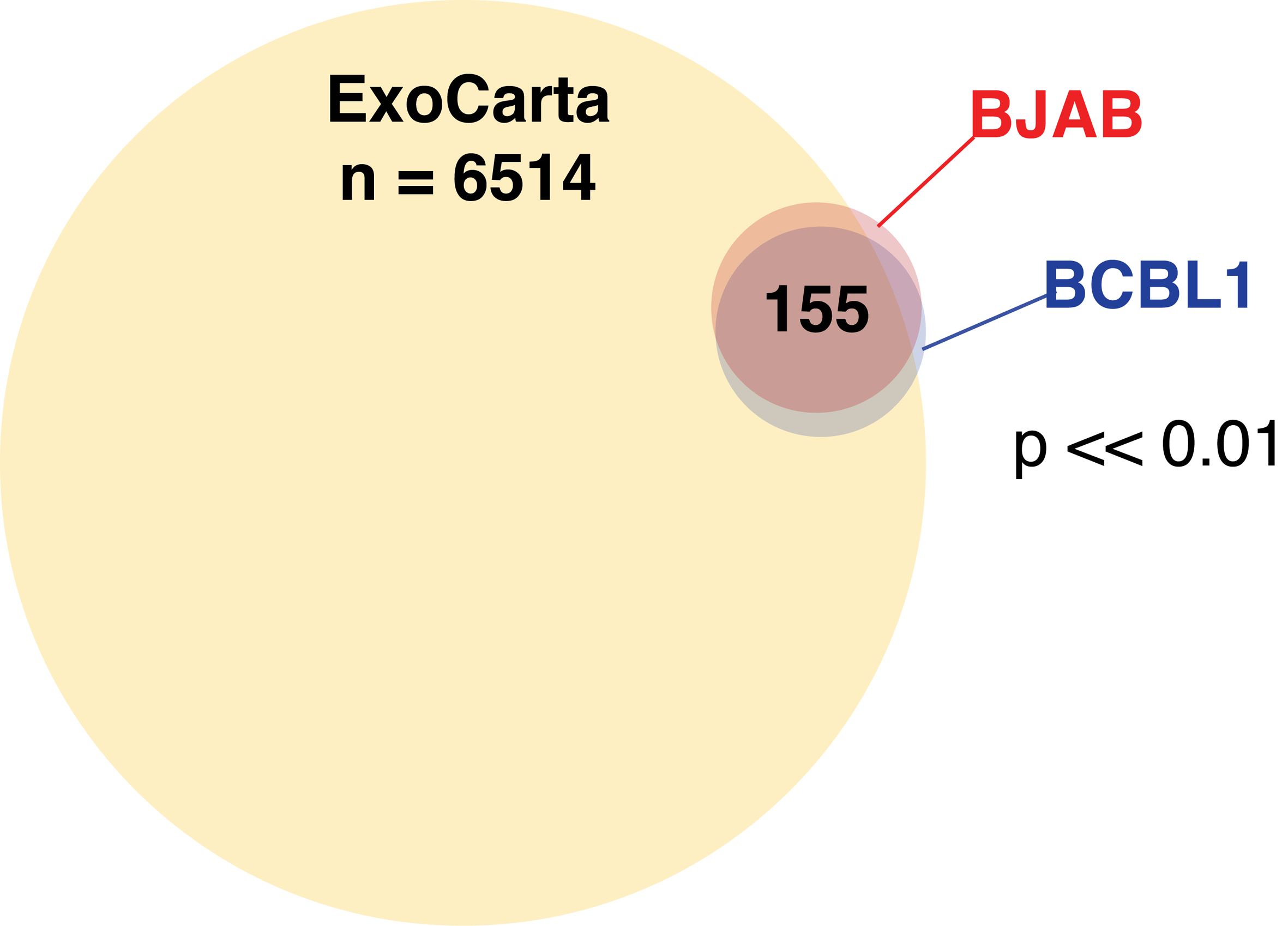

Supplement: S2 Fig — Filtrated EV were run out on a denaturing polyacrylamide gel and gel slices were analyzed by mass spectrometry. Top high-confidence hits were analyzed against known exosome constituents in the ExoCarta database. Statistical analysis for overlay was done using the hypergeometric test. (TIF) [file ppat.1007536.s002.tif]

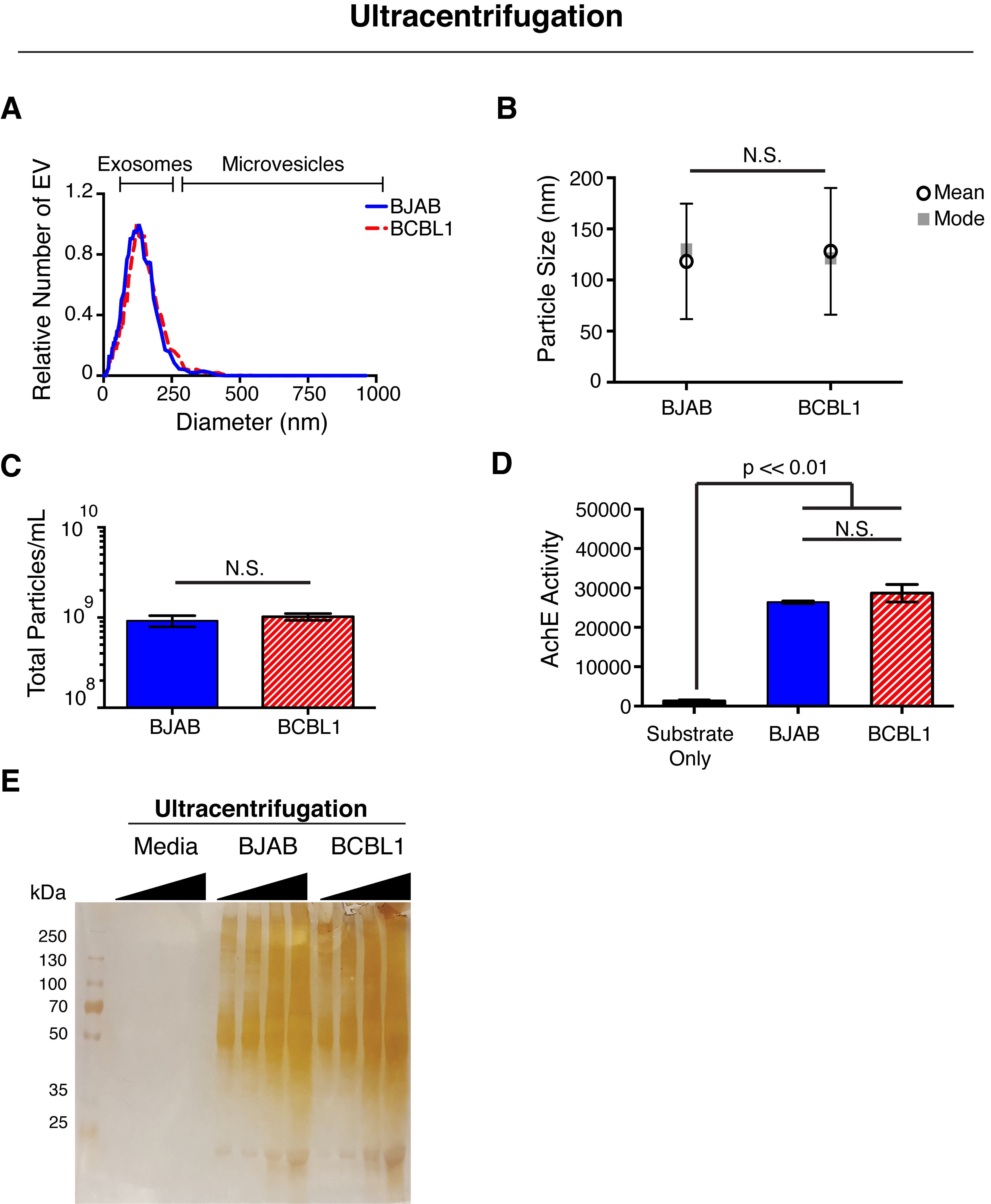

Supplement: S3 Fig — (A) Size distribution analysis post-ultracentrifugation was done using the PEG-precipitated EV from BJAB and BCBL1 cells. Expected size ranges of exosomes and microvesicles are shown. (B) Mean (open circle) and mode (gray square) sizes of the ultracentrifuged EV from the PEG-precipitate. (C) Total EV particles per mL of supernatant from BJAB (solid blue) or BCBL1 (dashed red) cells from the post-ultracentrifugation, PEG precipitate. (D) Relative acetylcholine esterase (AchE) activity of the post-ultracentrifuged, PEG-precipitated EV. Substrate only is shown for reference against BJAB (solid blue) and BCBL1 (dashed red) EV. (E) Silver stain analysis of the post-ultracentrifuged, PEG precipitated EV from BJAB and BCBL1. PEG-precipitated cell culture media was used as a control for background. (TIF) [file ppat.1007536.s003.tif]

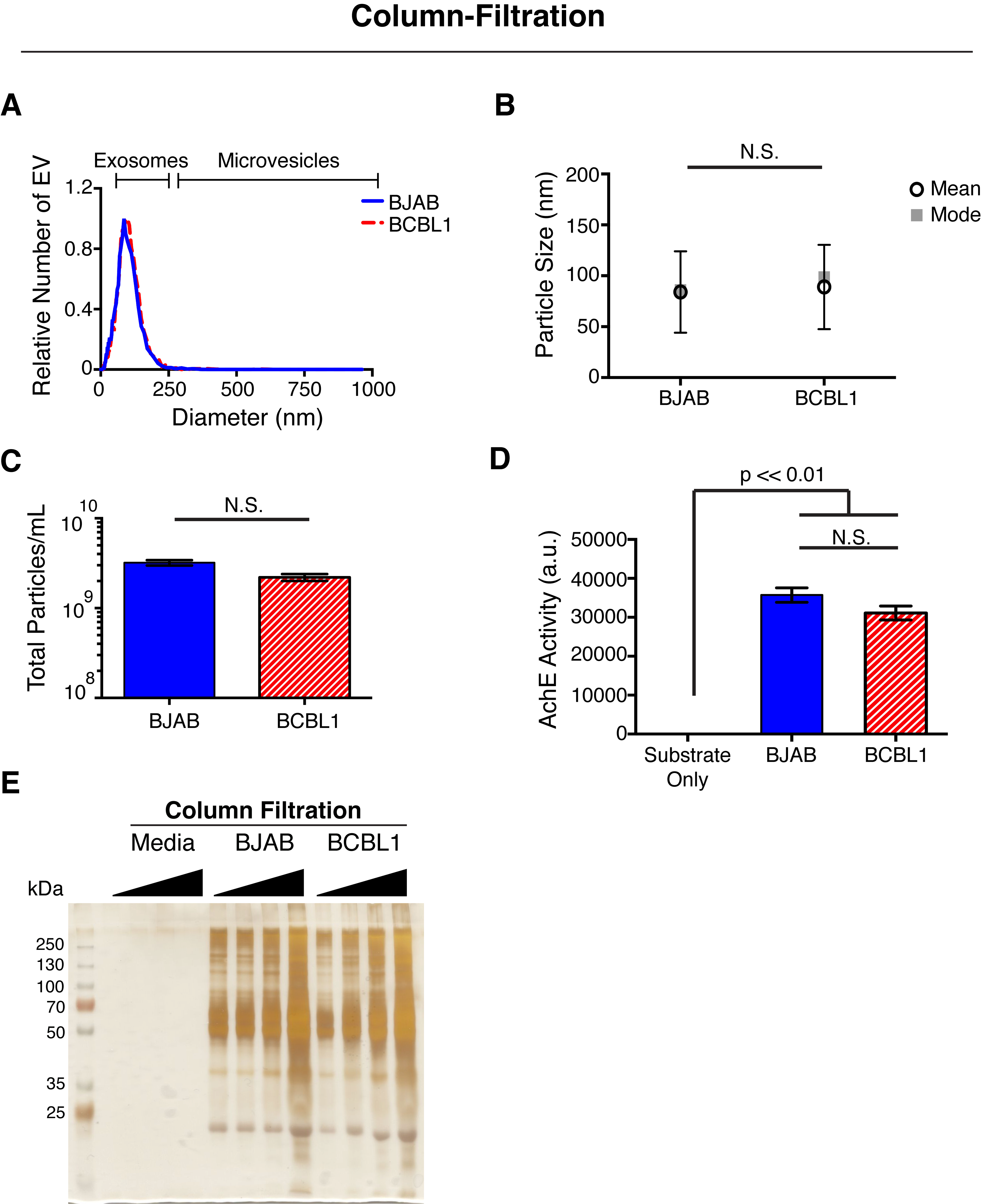

Supplement: S4 Fig — (A) Size distribution analysis post-column filtration was done using the PEG-precipitated EV from BJAB and BCBL1 cells. Expected size ranges of exosomes and microvesicles are shown. (B) Mean (open circle) and mode (gray square) sizes of the column filtrated EV from the PEG-precipitate. (C) Total EV particles per mL of supernatant from BJAB (solid blue) or BCBL1 (dashed red) cells from the post-column filtrated, PEG precipitate. (D) Relative acetylcholine esterase (AchE) activity of the post-column filtrated, PEG-precipitated EV. Substrate only is shown for reference against BJAB (solid blue) and BCBL1 (dashed red) EV. (E) Silver stain analysis of the post-ultracentrifuged, PEG precipitated EV from BJAB and BCBL1. PEG-precipitated cell culture media was used as a control for background. (TIF) [file ppat.1007536.s004.tif]

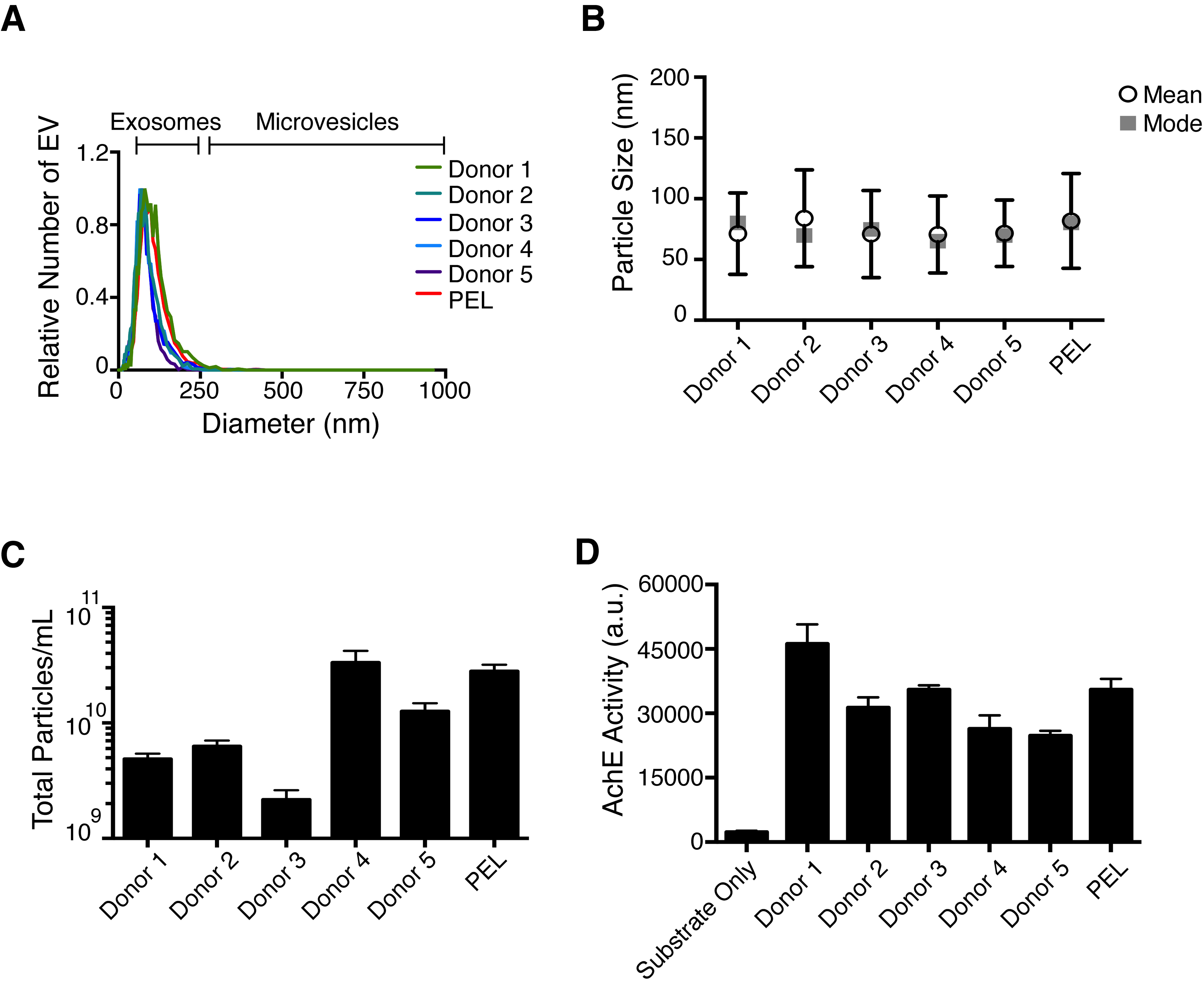

Supplement: S5 Fig — (A) Size distribution analysis post-column filtration was done using the PEG-precipitated EV from healthy donors and primary effusion lymphoma (PEL). Expected size ranges of exosomes and microvesicles are shown. (B) Mean (open circle) and mode (gray square) sizes of the column filtrated EV from the PEG-precipitate. (C) Total EV particles per mL of supernatant from the healthy donors and the PEL samples from the post-column filtrated, PEG precipitate. (D) Relative acetylcholine esterase (AchE) activity of the post-column filtrated, PEG-precipitated EV. Substrate only is shown for reference against healthy donors and PEL EV. (TIF) [file ppat.1007536.s005.tif]

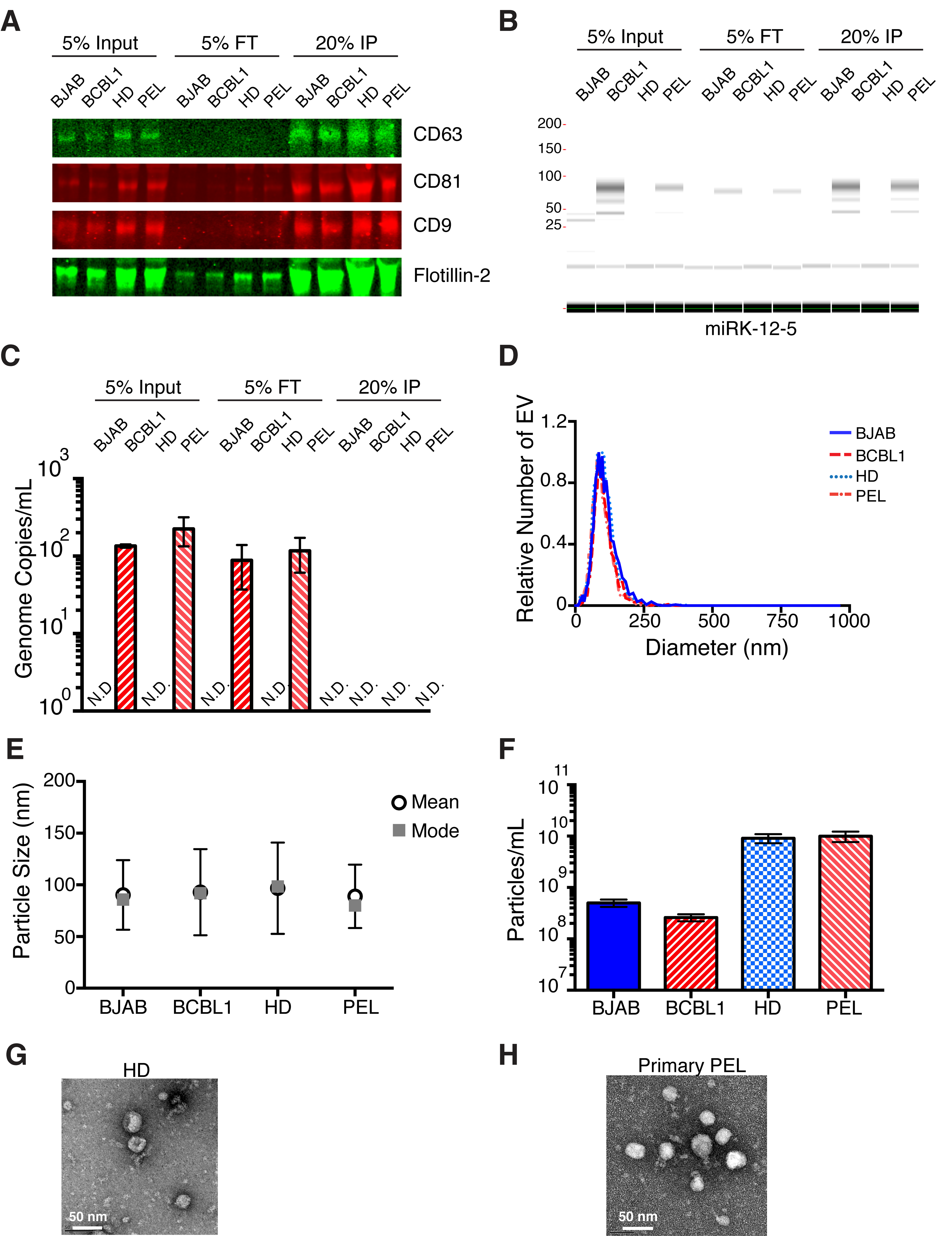

Supplement: S6 Fig — (A) EV were affinity captured using anti-CD63 magnetic beads and products were run out for protein and nucleic acid analysis. CD63, CD81, CD9, and Flotillin-2 were used to monitor the successful immunoprecipitation. (B) miRK12-5 was reverse transcribed from the fractions and amplified by qRT-PCR. Products were run on the Caliper LabChip GX. (C) KSHV DNA genomes were quantified from each fraction via qPCR. (D) Size distribution analysis post-affinity capture was done using the BJAB, BCBL1, HD, PEL EV. Expected size ranges of exosomes and microvesicles are shown. (C) Mean (open circle) and mode (gray square) sizes of the affinity captured EV from the PEG-precipitate. (D) EV particles per mL of supernatant from the healthy donors and the PEL samples from the post-column filtrated, PEG precipitate. (E) Negative stain electron micrographs of affinity captured EV from HD. (F) Negative stain electron micrographs of affinity captured EV from PEL. (TIF) [file ppat.1007536.s006.tif]

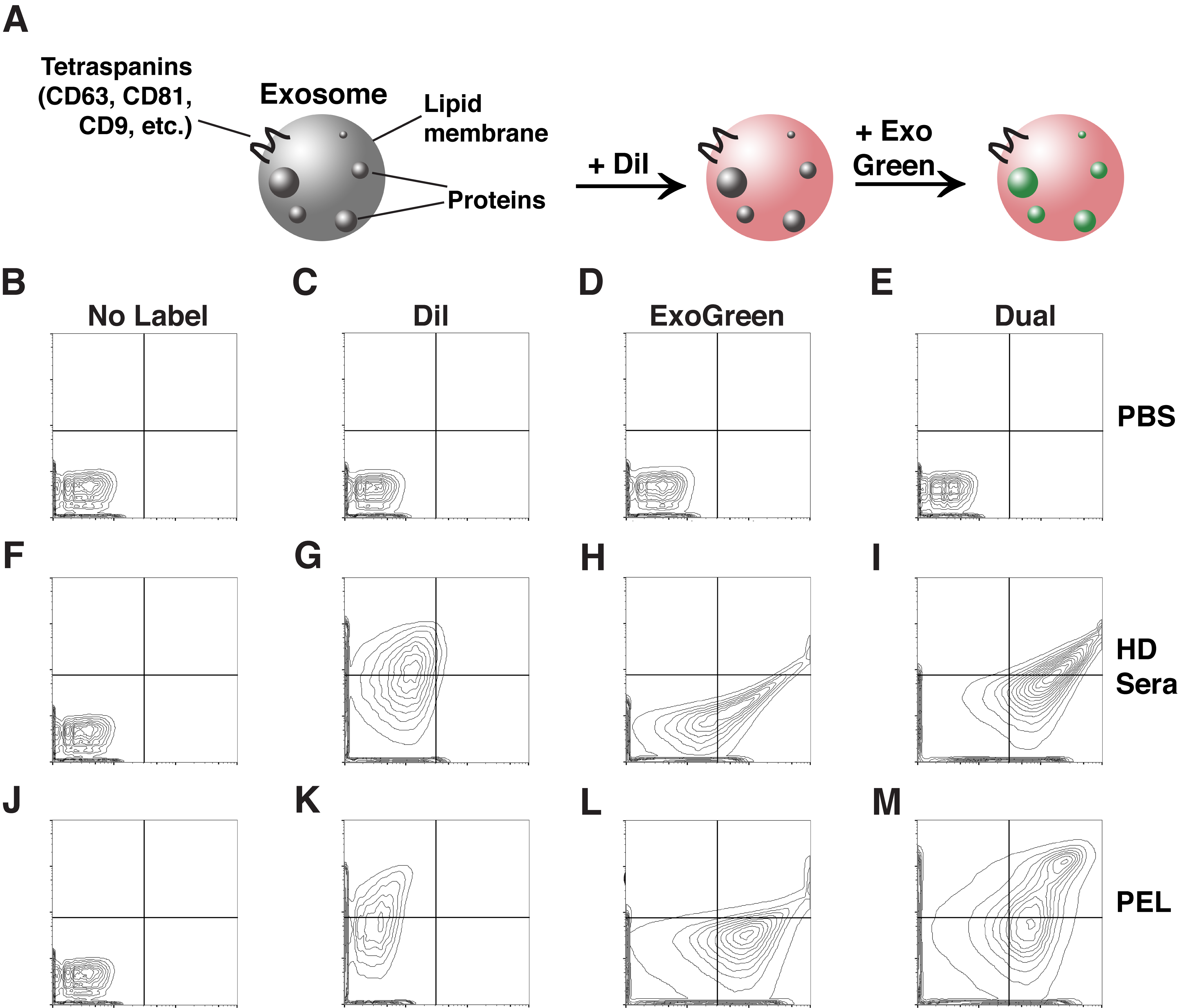

Supplement: S7 Fig — (A) Scheme for labeling of affinity purified EV. EV were purified using antibodies directed to the tetraspanins presented on the surface of EV (CD63, CD9, and CD81). The lipid dye Dil will fluorescently label the EV red and the AchE reporter ExoGreen will fluorescently label internal proteins green. (B) The affinity capture-negative control (PBS) without any label was conjugated to anti-CD63 beads and run for flow cytometry analysis. (C) The affinity capture-negative control (PBS) was incubated with Dil and conjugated to anti-CD63 beads and run for flow cytometry analysis. (D) The affinity capture-negative control (PBS) was incubated with ExoGreen and conjugated to anti-CD63 beads and run for flow cytometry analysis. (E) The affinity capture-negative control (PBS) was incubated with both Dil and ExoGreen and conjugated to anti-CD63 beads and run for flow cytometry analysis. (F) The affinity capture of HD EV without any label was conjugated to anti-CD63 beads and run for flow cytometry analysis. (G) The affinity capture of HD EV was incubated with Dil and conjugated to anti-CD63 beads and run for flow cytometry analysis. (H) The affinity capture of HD EV was incubated with ExoGreen and conjugated to anti-CD63 beads and run for flow cytometry analysis. (I) The affinity capture of HD EV was incubated with both Dil and ExoGreen and conjugated to anti-CD63 beads and run for flow cytometry analysis. (J) The affinity capture of PEL EV without any label was conjugated to anti-CD63 beads and run for flow cytometry analysis. (K) The affinity capture of PEL EV was incubated with Dil and conjugated to anti-CD63 beads and run for flow cytometry analysis. (L) The affinity capture of PEL EV was incubated with ExoGreen and conjugated to anti-CD63 beads and run for flow cytometry analysis. (M) The affinity capture of PEL EV was incubated with both Dil and ExoGreen and conjugated to anti-CD63 beads and run for flow cytometry analysis. (TIF) [file ppat.1007536.s007.tif]

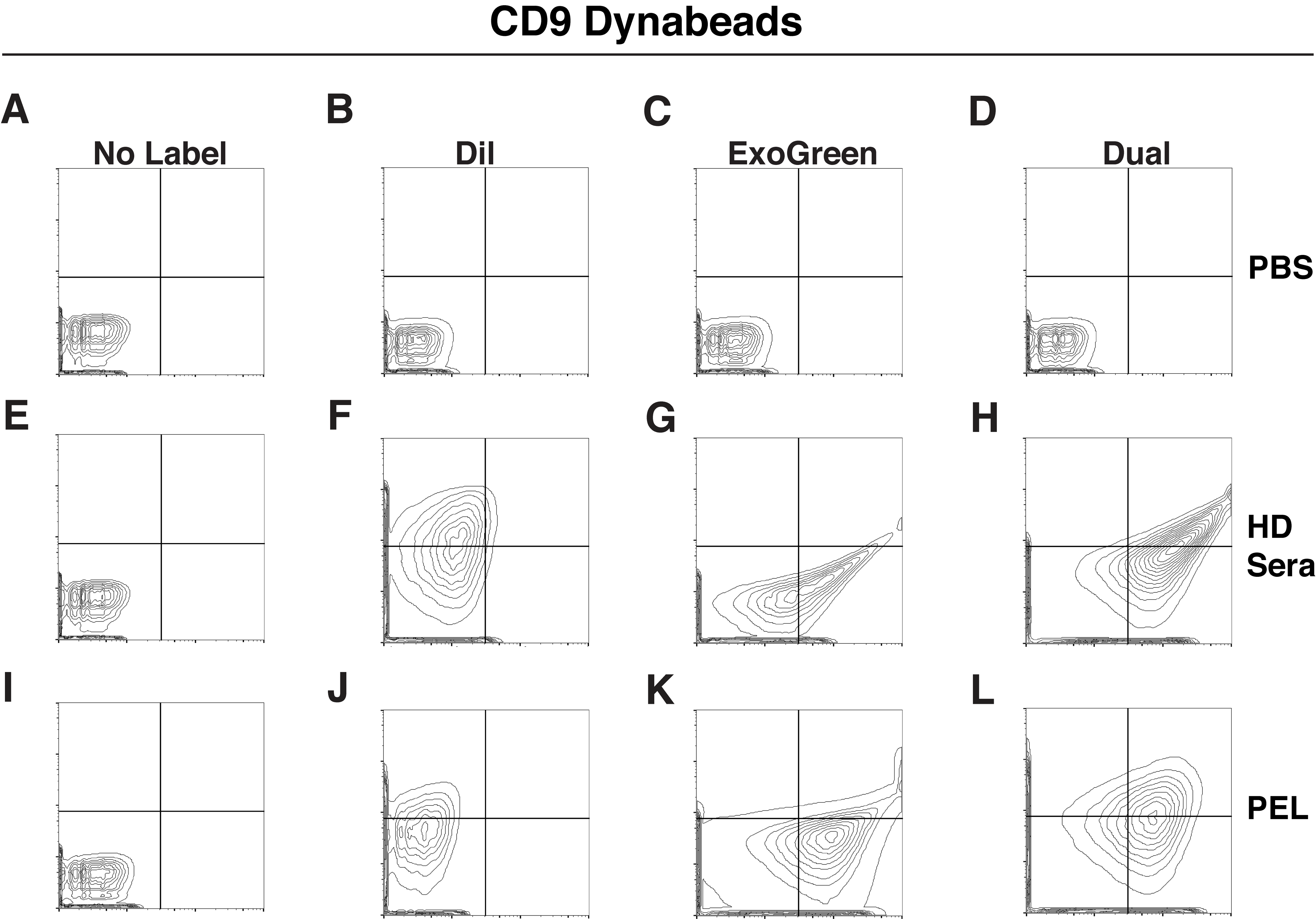

Supplement: S8 Fig — (A) The affinity capture-negative control (PBS) without any label was conjugated to anti-CD9 beads and run for flow cytometry analysis. (B) The affinity capture-negative control (PBS) was incubated with Dil and conjugated to anti-CD9 beads and run for flow cytometry analysis. (C) The affinity capture-negative control (PBS) was incubated with ExoGreen and conjugated to anti-CD9 beads and run for flow cytometry analysis. (D) The affinity capture-negative control (PBS) was incubated with both Dil and ExoGreen and conjugated to anti-CD9 beads and run for flow cytometry analysis. (E) The affinity capture of HD EV without any label was conjugated to anti-CD9 beads and run for flow cytometry analysis. (F) The affinity capture of HD EV was incubated with Dil and conjugated to anti-CD9 beads and run for flow cytometry analysis. (G) The affinity capture of HD EV was incubated with ExoGreen and conjugated to anti-CD9 beads and run for flow cytometry analysis. (H) The affinity capture of HD EV was incubated with both Dil and ExoGreen and conjugated to anti-CD9 beads and run for flow cytometry analysis. (I) The affinity capture of PEL EV without any label was conjugated to anti-CD9 beads and run for flow cytometry analysis. (J) The affinity capture of PEL EV was incubated with Dil and conjugated to anti-CD9 beads and run for flow cytometry analysis. (K) The affinity capture of PEL EV was incubated with ExoGreen and conjugated to anti-CD9 beads and run for flow cytometry analysis. (L) The affinity capture of PEL EV was incubated with both Dil and ExoGreen and conjugated to anti-CD9 beads and run for flow cytometry analysis. (TIF) [file ppat.1007536.s008.tif]

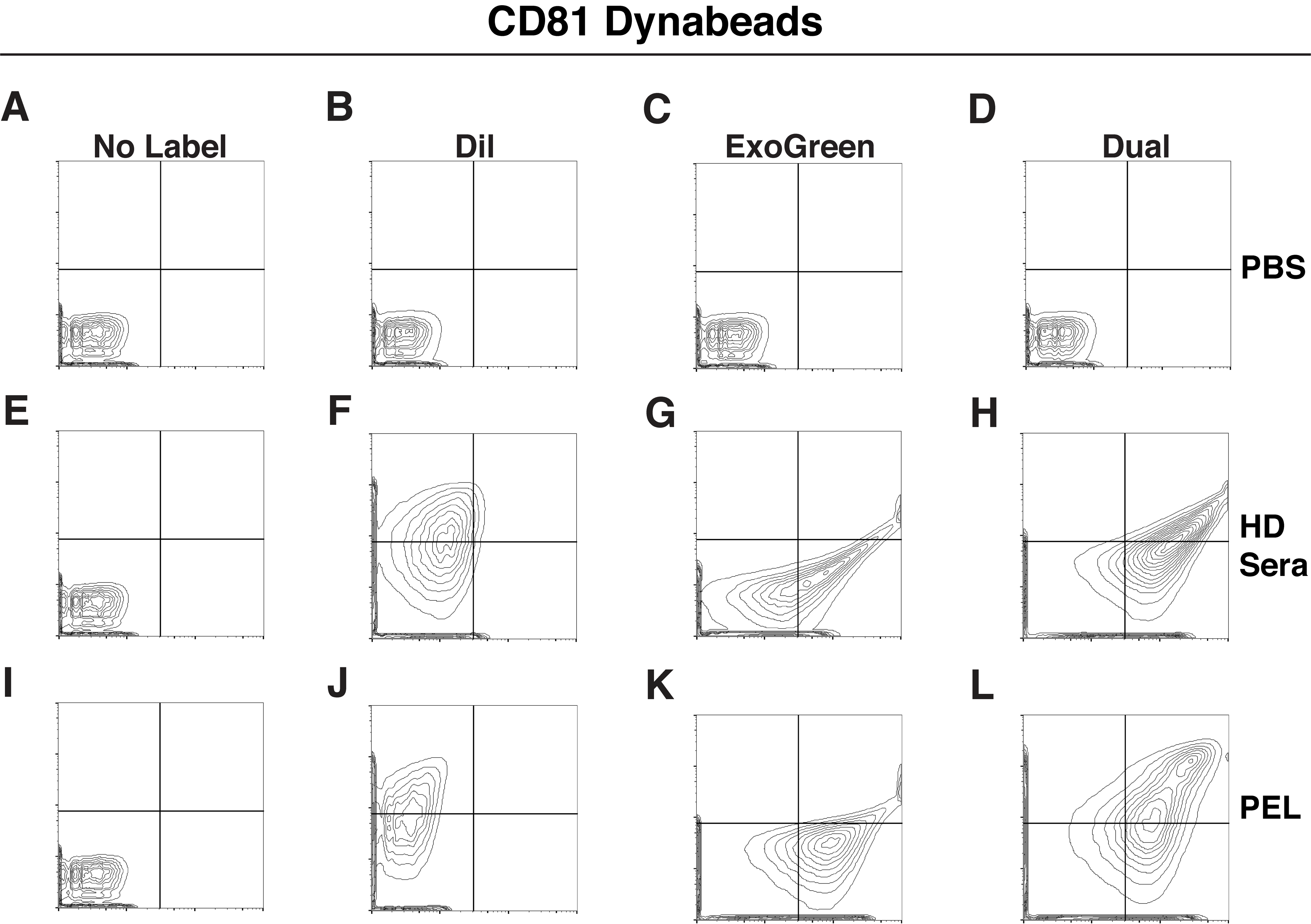

Supplement: S9 Fig — (A) The affinity capture-negative control (PBS) without any label was conjugated to anti-CD81 beads and run for flow cytometry analysis. (B) The affinity capture-negative control (PBS) was incubated with Dil and conjugated to anti-CD81 beads and run for flow cytometry analysis. (C) The affinity capture-negative control (PBS) was incubated with ExoGreen and conjugated to anti-CD81 beads and run for flow cytometry analysis. (D) The affinity capture-negative control (PBS) was incubated with both Dil and ExoGreen and conjugated to anti-CD81 beads and run for flow cytometry analysis. (E) The affinity capture of HD EV without any label was conjugated to anti-CD81 beads and run for flow cytometry analysis. (F) The affinity capture of HD EV was incubated with Dil and conjugated to anti-CD81 beads and run for flow cytometry analysis. (G) The affinity capture of HD EV was incubated with ExoGreen and conjugated to anti-CD81 beads and run for flow cytometry analysis. (H) The affinity capture of HD EV was incubated with both Dil and ExoGreen and conjugated to anti-CD81 beads and run for flow cytometry analysis. (I) The affinity capture of PEL EV without any label was conjugated to anti-CD81 beads and run for flow cytometry analysis. (J) The affinity capture of PEL EV was incubated with Dil and conjugated to anti-CD81 beads and run for flow cytometry analysis. (K) The affinity capture of PEL EV was incubated with ExoGreen and conjugated to anti-CD81 beads and run for flow cytometry analysis. (L) The affinity capture of PEL EV was incubated with both Dil and ExoGreen and conjugated to anti-CD81 beads and run for flow cytometry analysis. (TIF) [file ppat.1007536.s009.tif]

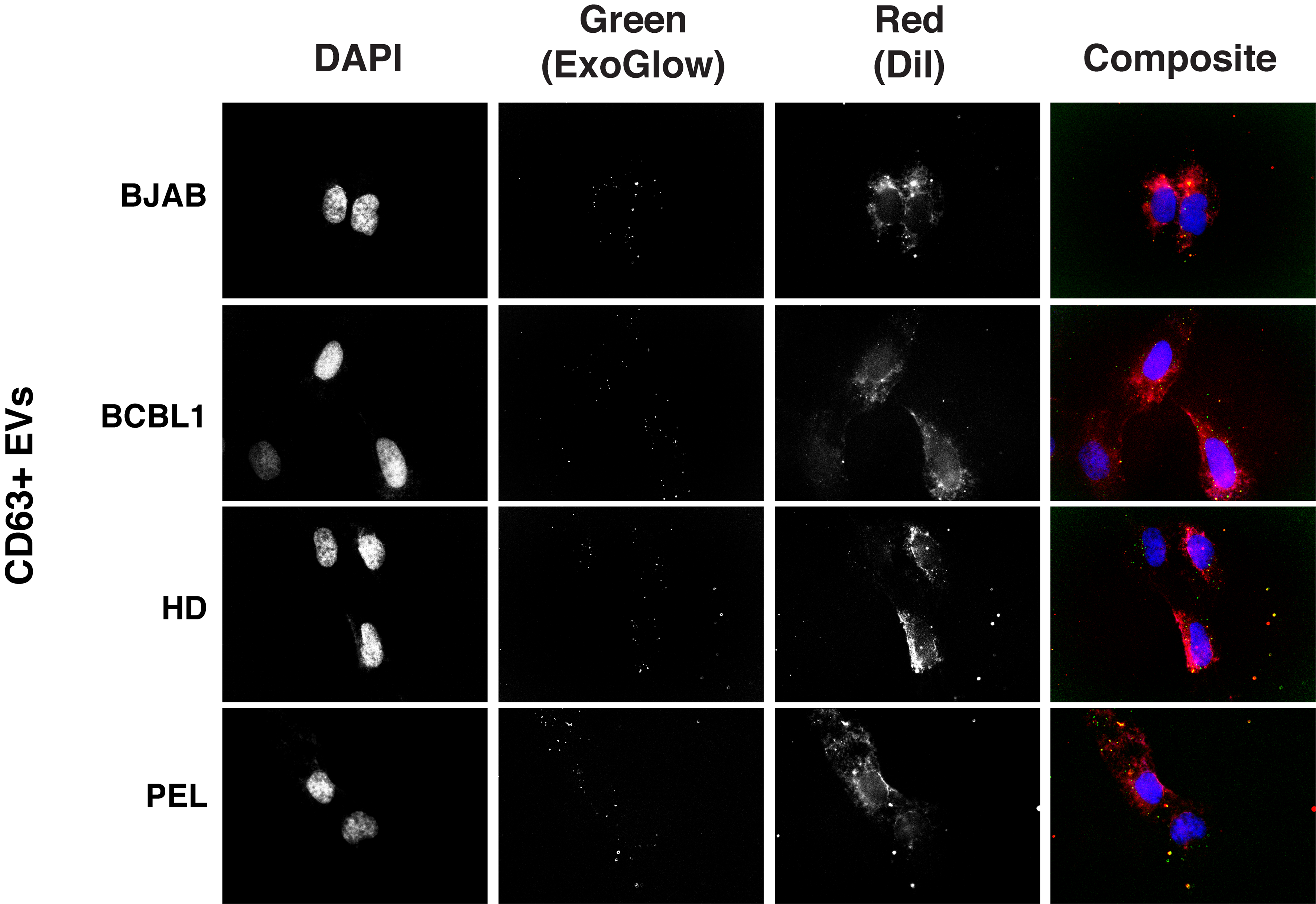

Supplement: S10 Fig — CD63-captured EV from BJAB, BCBL1 cultured cells, or from HD or primary PEL were added to hTERT-HUVEC for 24 hours and cells were assayed for uptake by fluorescence microcopy. 3-D images were taken and deconvoluted. The lipid dye Dil showed a more dispersed fluorescence pattern, whereas the protein dye ExoGreen remained in punctate structures. (TIF) [file ppat.1007536.s010.tif]

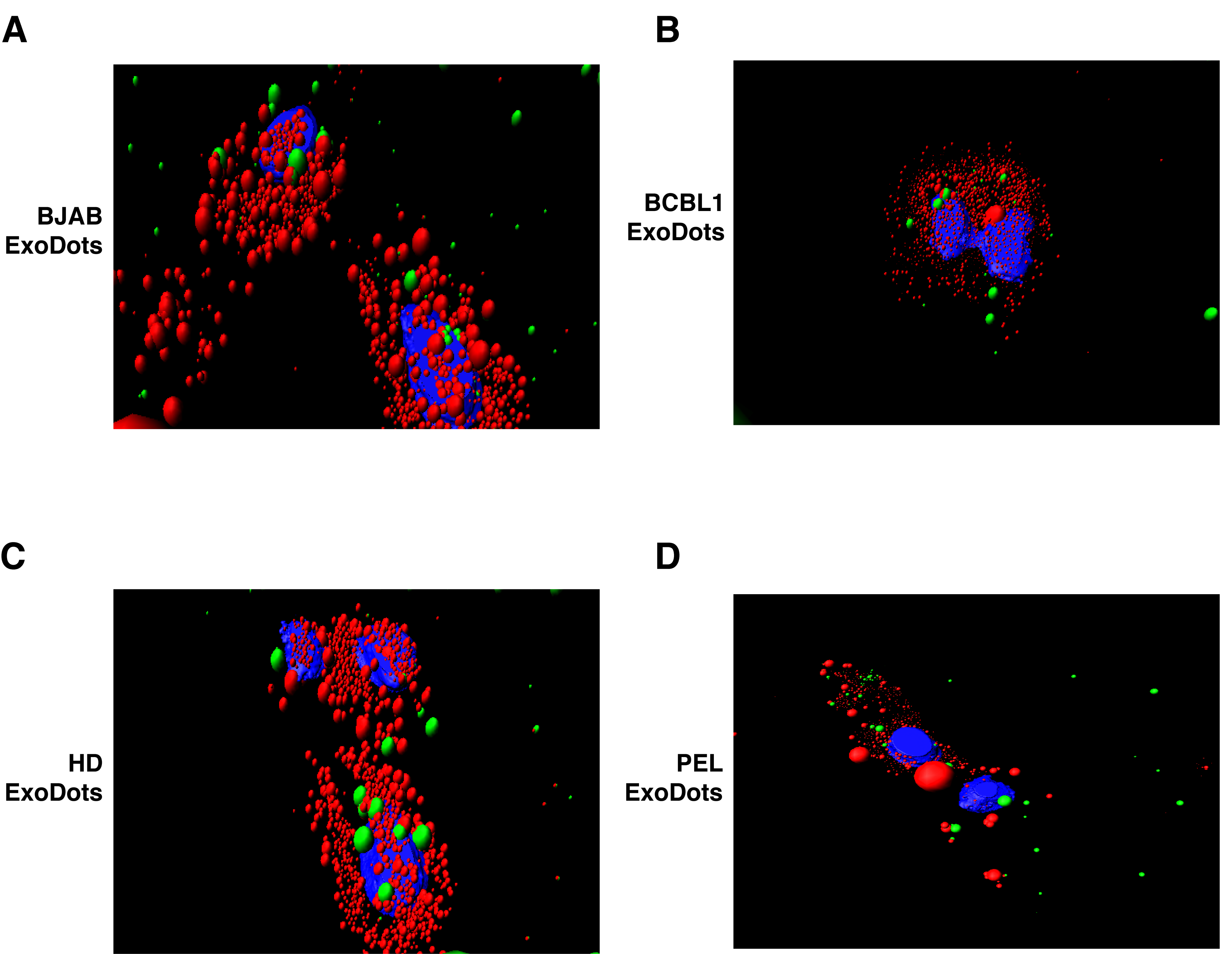

Supplement: S11 Fig — (A) 3-D dot representation of hTERT-HUVECs treated with dual labeled EV taken from BJAB cells. (B) 3-D dot representation of hTERT-HUVECs treated with dual labeled EV taken from BCBL1 cells. (C) 3-D dot representation of hTERT-HUVECs treated with dual labeled EV taken from HD plasma. (D) 3-D dot representation of hTERT-HUVECs treated with dual labeled EV taken from primary PEL fluid. (TIF) [file ppat.1007536.s011.tif]

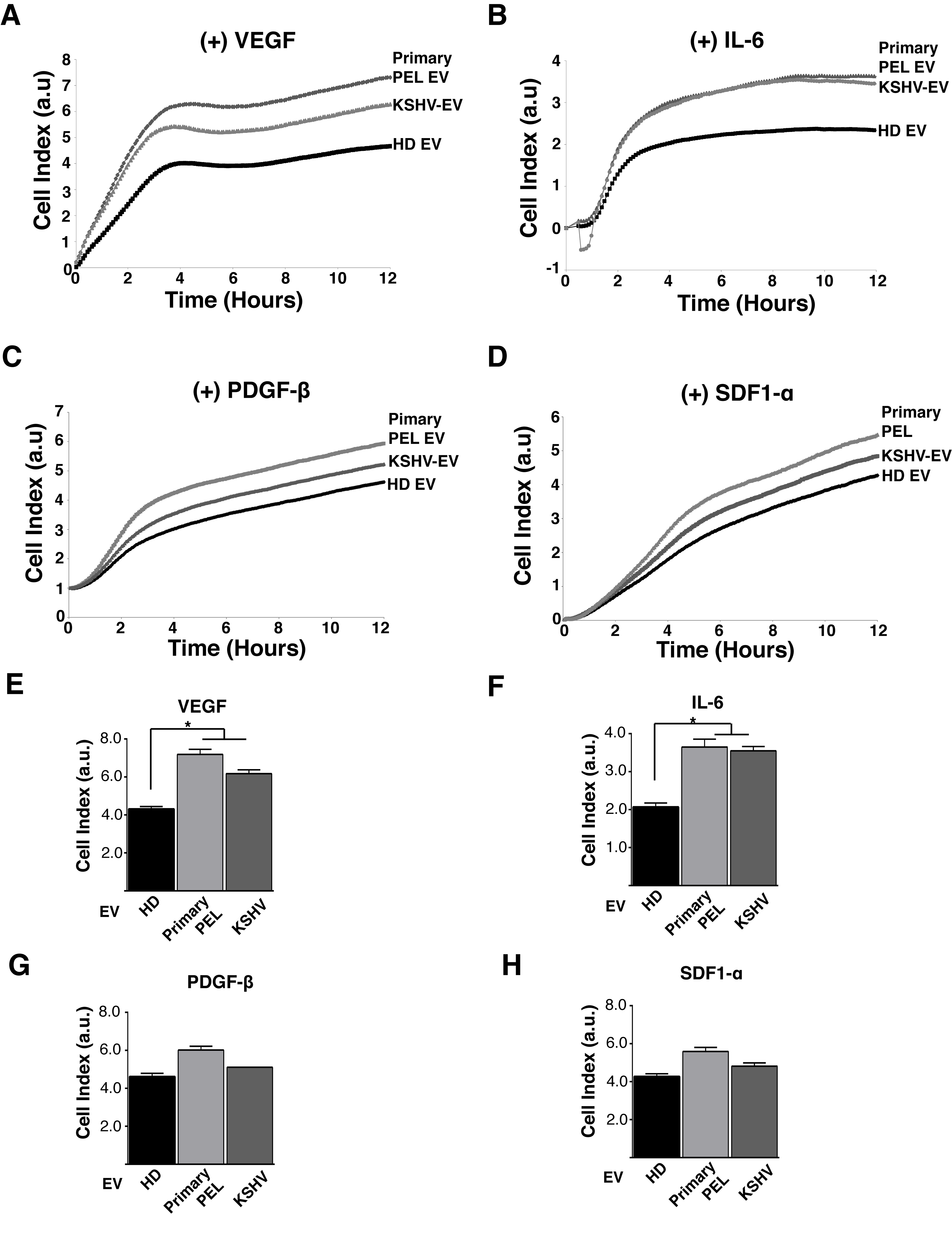

Supplement: S12 Fig — (A) hTERT-HUVECs were plated in a specialized xCelligence CIM-plate in the presence of VEGF. In the chamber below the cells were HD EV, KSHV-EV, or primary PEL EV. (B) Same as for (A), but with IL-6. (C) Same as for (A), but with PDGF-β. (D) Same as for (A), but for SDF1-α. (E) Endpoint analysis of the relative migration of hTERT-HUVECs in the presence of VEGF toward the bottom chamber containing HD EV, KSHV-EV, or primary PEL EV (asterisks indicates that HD was significantly different from KSHV-EV and primary PEL EV treated samples). (F) Endpoint analysis of the relative migration of hTERT-HUVECs in the presence of IL-6 toward the bottom chamber containing HD EV, KSHV-EV, or primary PEL EV (asterisks indicates that HD was significantly different from KSHV-EV and primary PEL EV treated samples). (G) Endpoint analysis of the relative migration of hTERT-HUVECs in the presence of PEGF-β toward the bottom chamber containing HD EV, KSHV-EV, or primary PEL EV. (H) Endpoint analysis of the relative migration of hTERT-HUVECs in the presence of SDF1-α toward the bottom chamber containing HD EV, KSHV-EV, or primary PEL EV. (TIF) [file ppat.1007536.s012.tif]

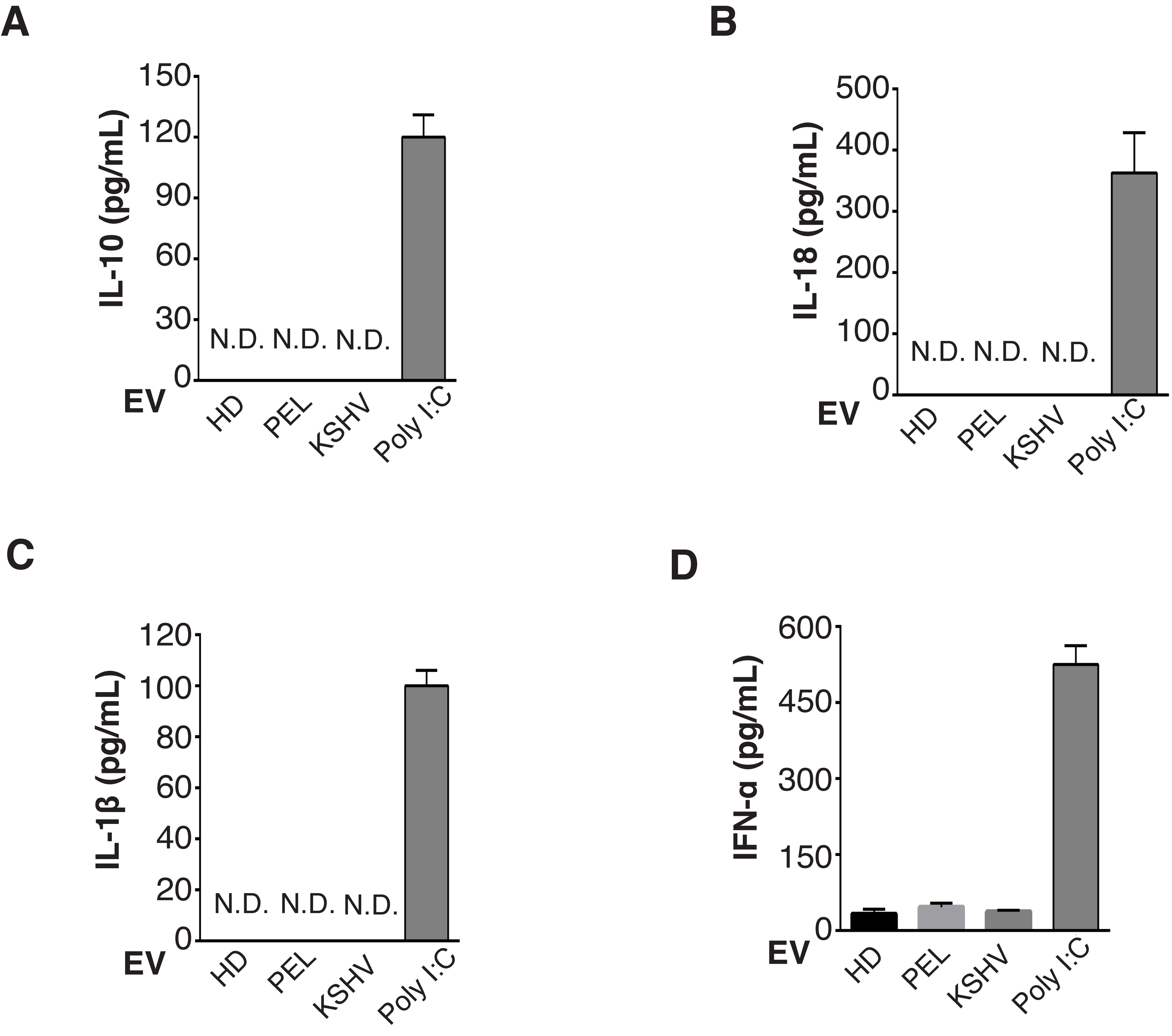

Supplement: S13 Fig — (A) hTERT-HUVECs were incubated with HD EV, KSHV-EV, or primary PEL EV and supernatant was assayed for the presence of the cytokine IL-10. ELISA determined amounts of the cytokine. As a positive control, cells were transfected with the double-stranded RNA mimic Poly I:C. (B) hTERT-HUVECs were incubated with HD EV, KSHV-EV, or primary PEL EV and supernatant was assayed for the presence of the cytokine IL-18. ELISA determined amounts of the cytokine. As a positive control, cells were transfected with the double-stranded RNA mimic Poly I:C. (C) hTERT-HUVECs were incubated with HD EV, KSHV-EV, or primary PEL EV and supernatant was assayed for the presence of the cytokine IL-1β. ELISA determined amounts of the cytokine. As a positive control, cells were transfected with the double-stranded RNA mimic Poly I:C. (D) hTERT-HUVECs were incubated with HD EV, KSHV-EV, or primary PEL EV and supernatant was assayed for the presence of the cytokine INF-α. ELISA determined amounts of the cytokine. As a positive control, cells were transfected with the double-stranded RNA mimic Poly I:C. (TIF) [file ppat.1007536.s013.tif]

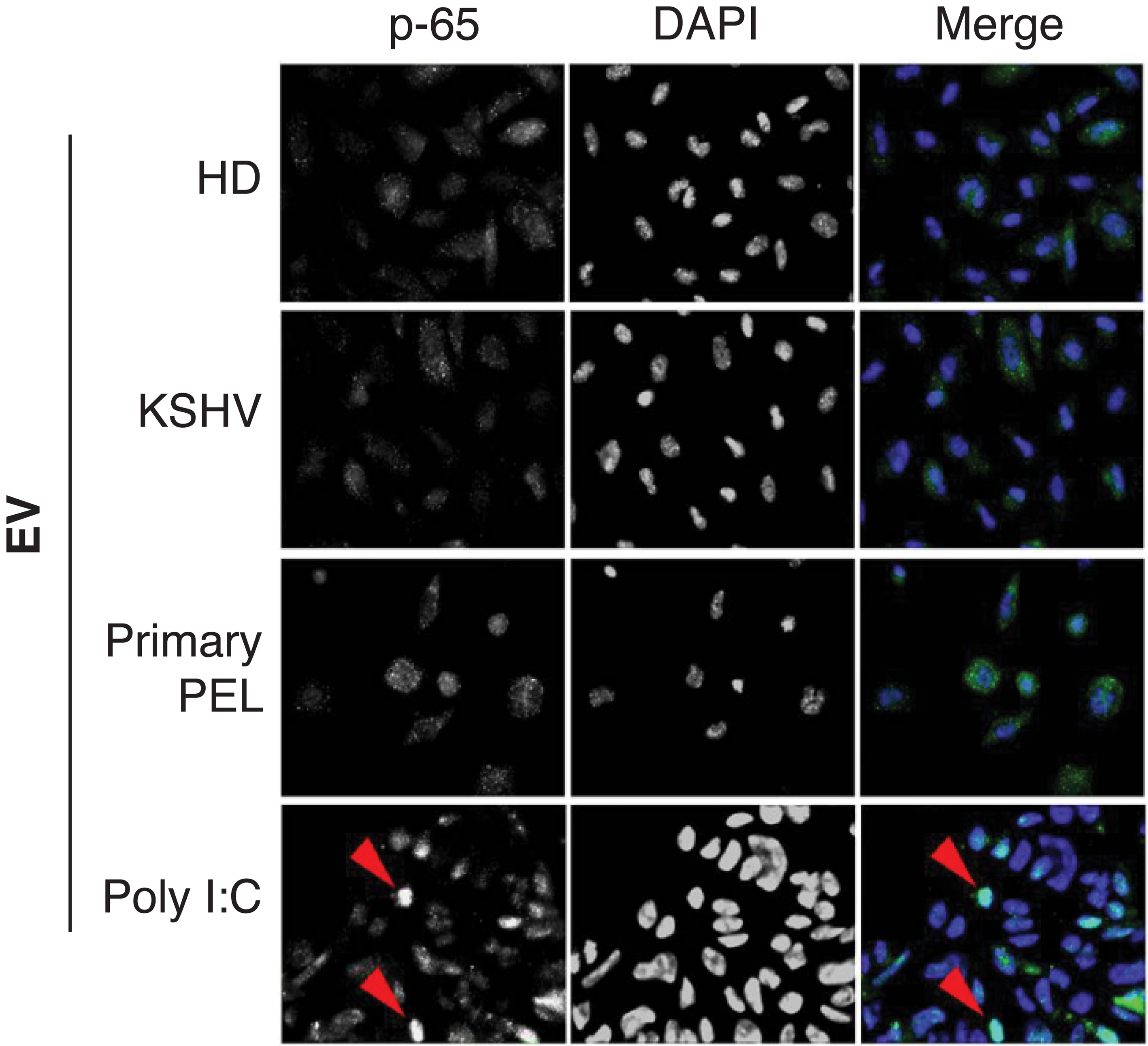

Supplement: S14 Fig — hTERT-HUVECs were incubated with HD EV, KSHV-EV, or primary PEL EV and assayed for induced translocation of the p65 subunit of NF-κB. As a positive control, cells were transfected with Poly I:C. (TIF) [file ppat.1007536.s014.tif]

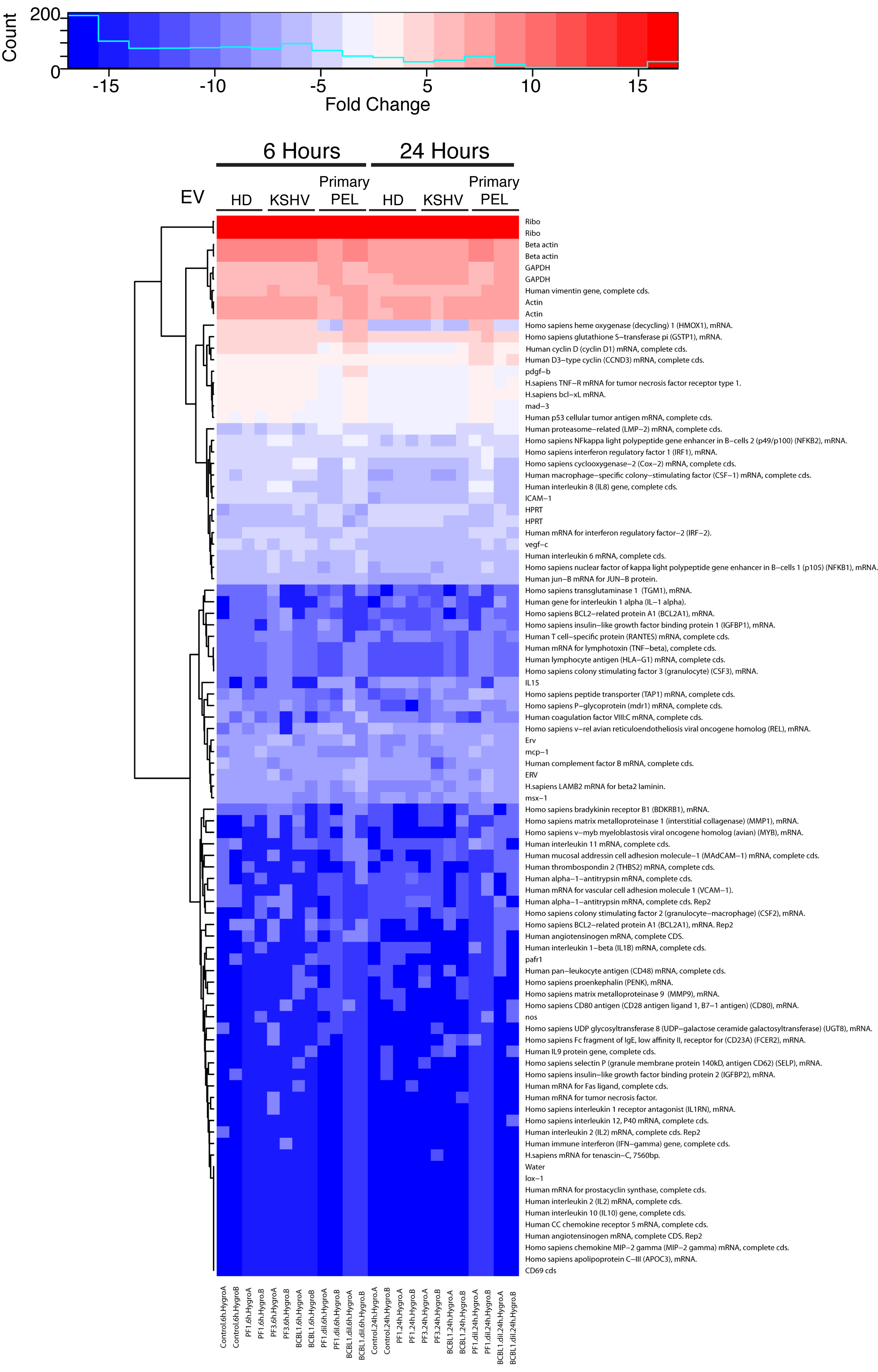

Supplement: S15 Fig — Array of 90 known NF-κB regulated genes were monitored for activation by HD EV, KSHV-EV, or primary PEL EV at 6 and 24 hours post EV treatment. Fold change of the indicated gene is shown in a heatmap. (TIF) [file ppat.1007536.s015.tif]

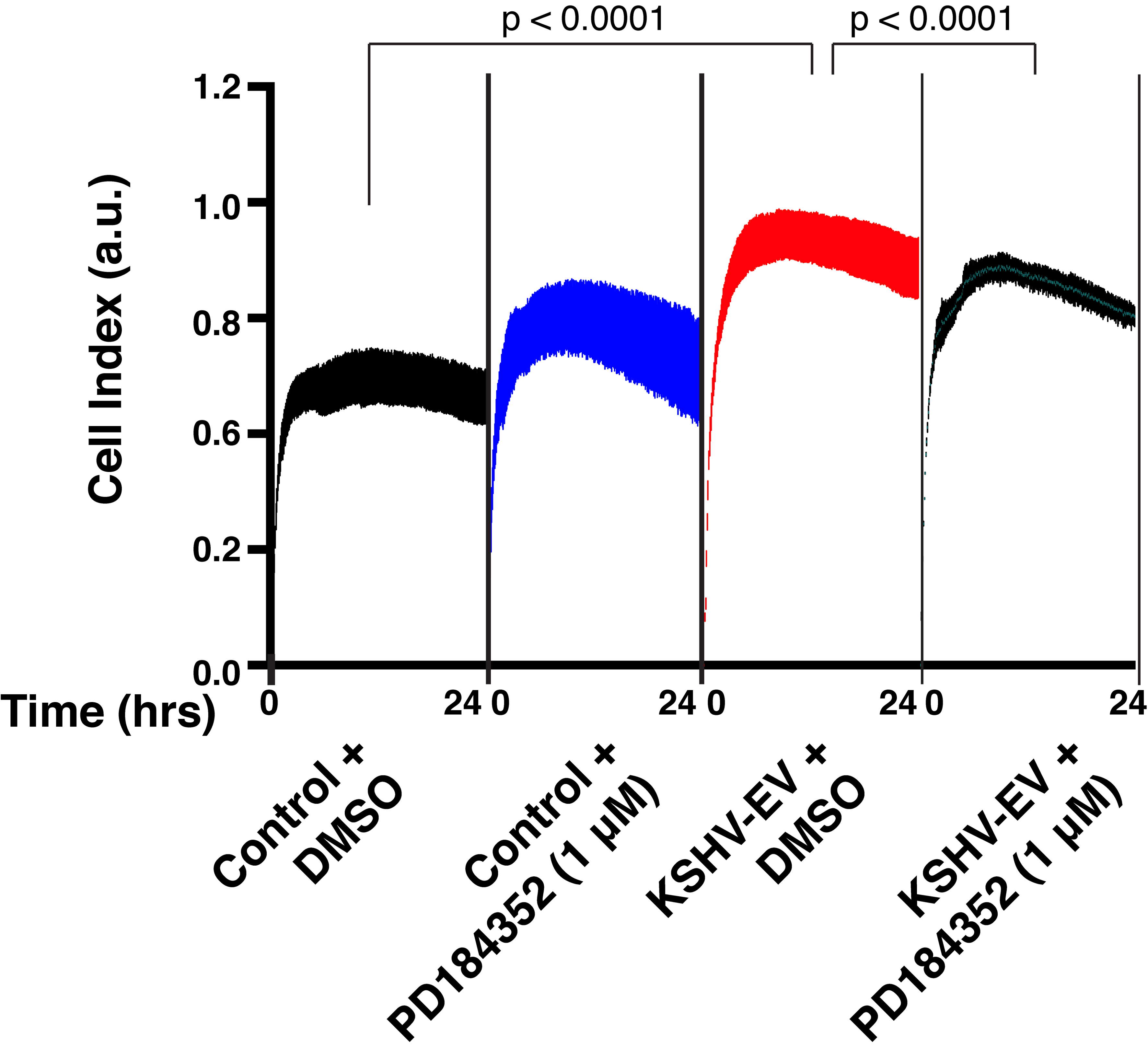

Supplement: S16 Fig — hTERT-HUVECs seeded in a specialized xCelligence CIM plate in the presence of DMSO or PD184352 (1 μM in DMSO). Cells were pre-treated with the inhibitor and subsequently exposed to control- or KSHV-EV. Cell index was monitored over the course of 24 hours. Shaded areas represent S.D. (TIF) [file ppat.1007536.s016.tif]

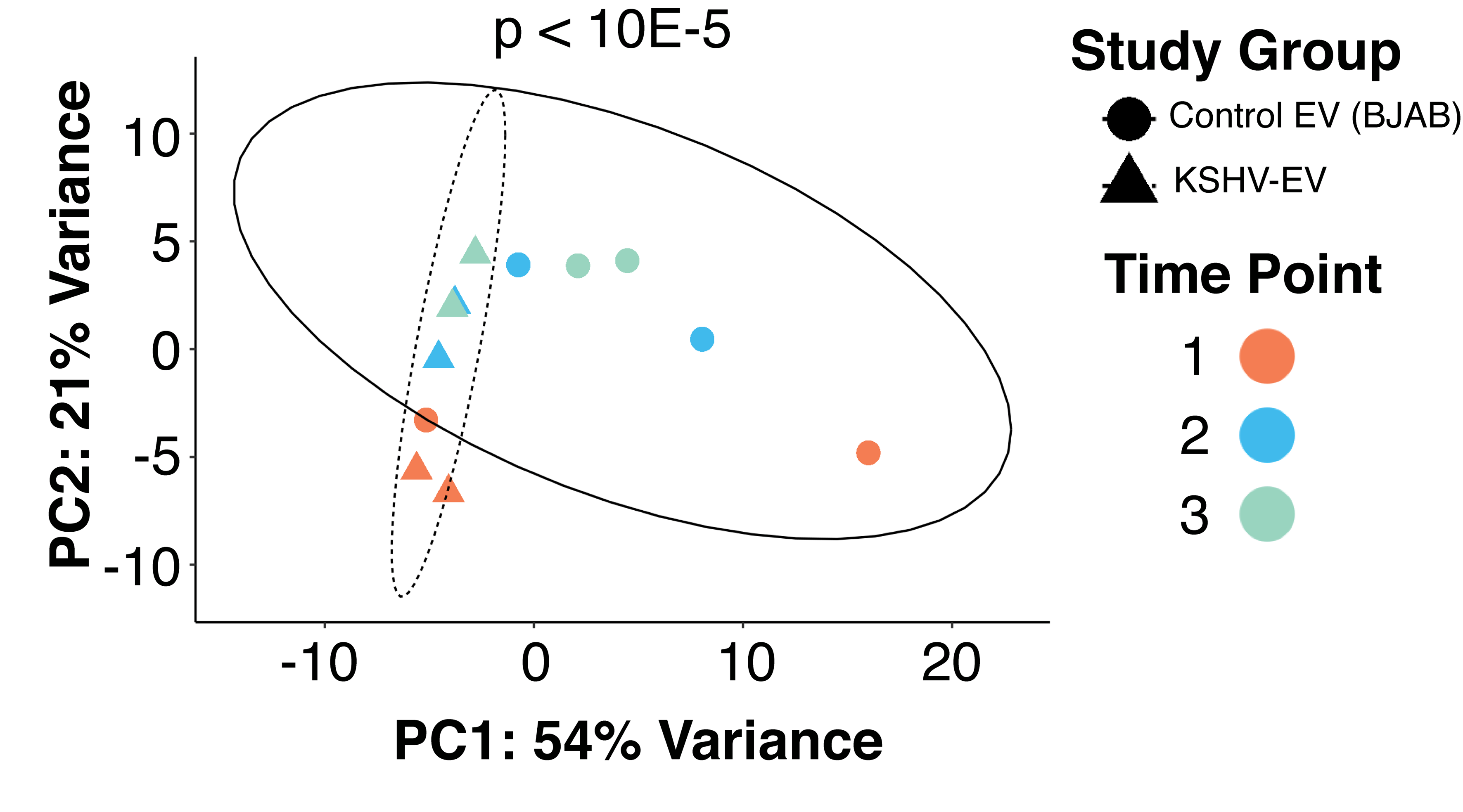

Supplement: S17 Fig — PCA plot of RNAseq reads from cells treated with BJAB (control) EV or KSHV-EV. hTERT-HUVECs were treated with Control EV or KSHV-EV over the course of 12 days. Ellipsoids represent clustering of the treatment groups (p < 0.05). (TIF) [file ppat.1007536.s017.tif]

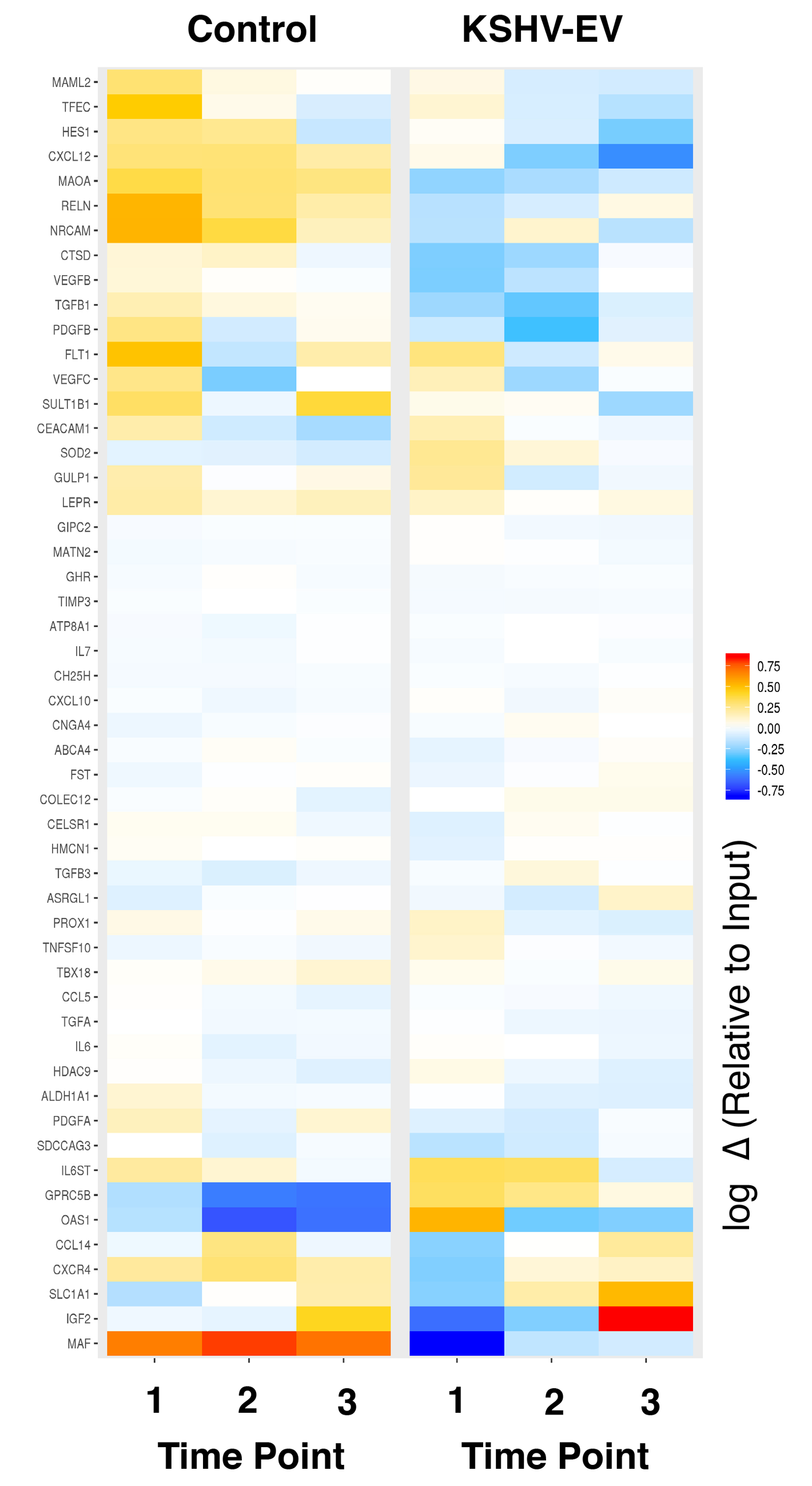

Supplement: S18 Fig — Heatmap of mRNAs that are significantly altered during KSHV infection of endothelial cells or by the viral miRNA as previously reported [6, 28, 29]. Shown is a comparison of control and KSHV-EV treated cells. Of the genes identified, only 3 reached statistical significance, which contain a * next their gene name. (TIF) [file ppat.1007536.s018.tif]

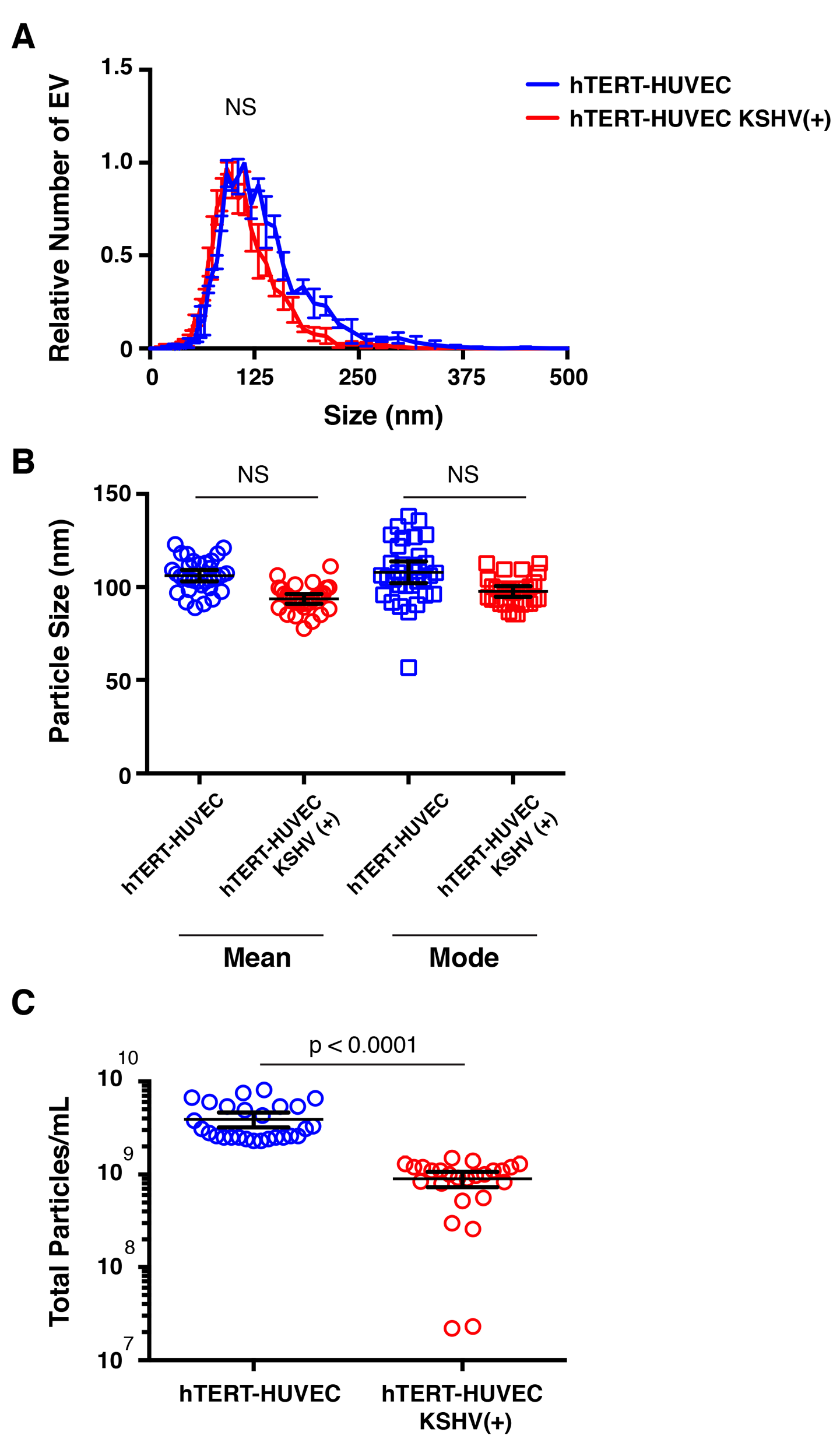

Supplement: S19 Fig — (A) Size distribution profile of EV isolated from hTERT-HUVECs or hTERT-HUVECs chronically infected with KSHV (hTERT-HUVEC KSHV(+)). (B) Mean and Mode sizes of EV isolated from hTERT-HUVECs or hTERT-HUVEC KSHV (+). (C) Concentration of EV released from hTERT-HUVECs or hTERT-HUVECS KSHV (+). Significant differences were determined by T-test. (TIF) [file ppat.1007536.s019.tif]

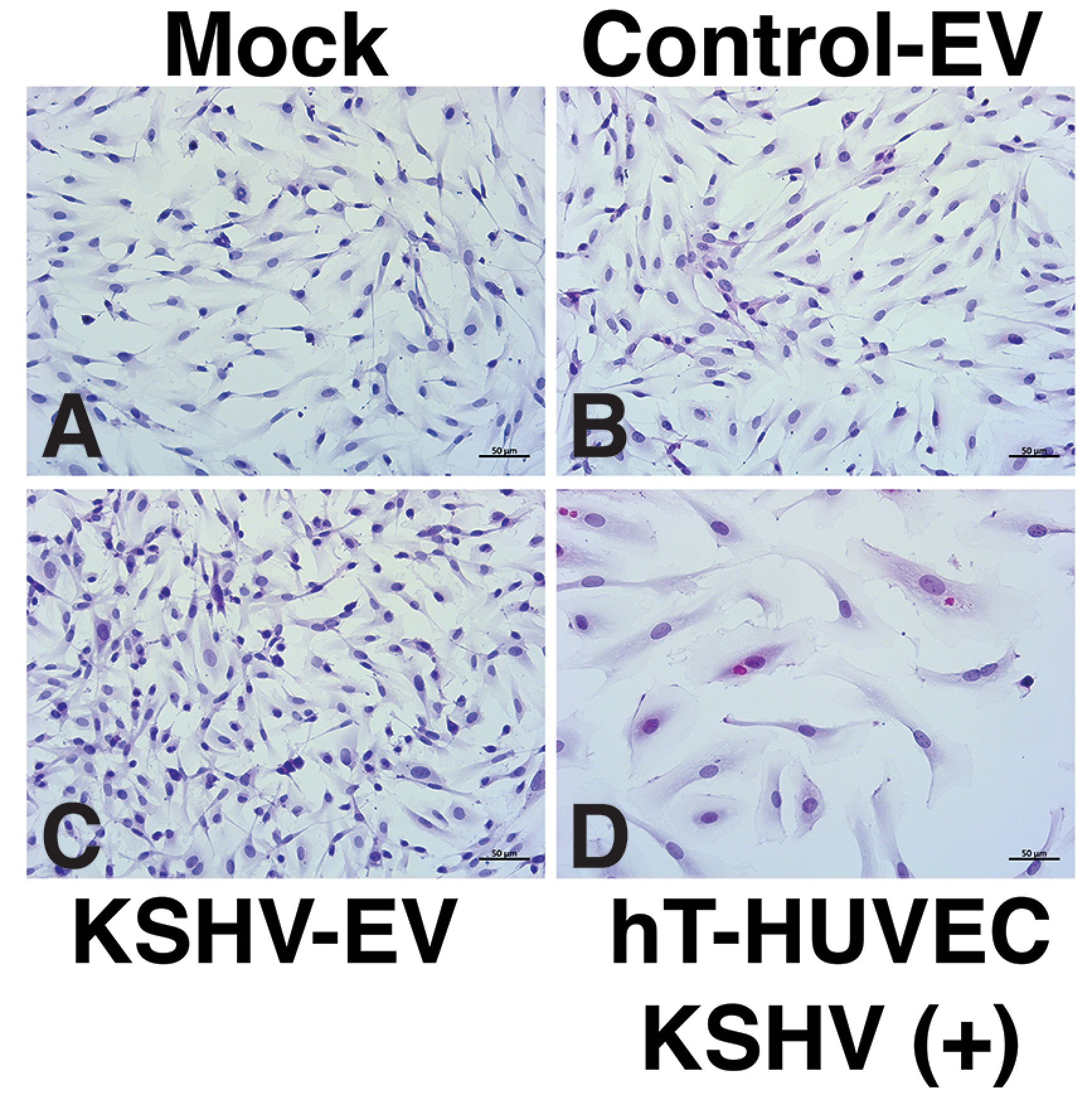

Supplement: S20 Fig — (A) hTERT-HUVEC were stained with Hematoxylin and eosin (H and E) after four days of continuous mock treatment. Objective = 40X. (B) hTERT-HUVECs were stained with H and E after four continuous days of Control-(BJAB) EV treatment. Objective = 40X. (C) hTERT-HUVECs were stained with H and E after four continuous days of KSHV-EV treatment. Objective = 40X. (D) hTERT-HUVEC KSHV (+) were stained with H and E as a control. Objective = 40X. (TIF) [file ppat.1007536.s020.tif]
