# Supplementary material for: Extracellular vesicles from Kaposi Sarcoma-associated herpesvirus lymphoma induce long-term endothelial cell reprogramming
Source: PLoS Pathog. 2019 Feb 4;15(2):e1007536. doi: 10.1371/journal.ppat.1007536 (PMC6361468; doi:10.1371/journal.ppat.1007536)
Supplement: S1 Table — (DOCX) [file ppat.1007536.s021.docx]

**Table S1**

| **Antibody** | **Manufacturer** | **Catalog** |
| --- | --- | --- |
| CD63 | Santa Cruz Biotech | SC-15363 |
| CD9 | Santa Cruz Biotech | SC-9148 |
| GAPDH | Santa Cruz Biotech | Sc-47724 |
| CD81 | Santa Cruz Biotech | Sc-166029 |
| Alix | Cell Signaling | 2171 |
| Flotillin-2 | Cell Signaling | 3244 |
| Actin | Cell Signaling | 4970 |
| JUNB | Cell Signaling | 3753 |
| Phosphor-ERK (p-ERK) | Cell Signaling | 4370 |
| ERK1/2 | Cell Signaling | 4696 |
| TBK1 | Cell Signaling | 3013S |
| Phosphor-TBK1 (p-TBK1) | Cell Signaling | 5483 |
| IRF3 | Cell Signaling | 4962 |
| Phosphor-p65 (p-p65) | Cell Signaling | 3033 |
| Tsg101 | ABcam | Ab225877 |
| Actin | ABcam | 8226 |
| Phosphor-IRF3 (p-IRF3) | Epitomics/ABcam | 2562-1 |
| Donkey Anti-mouse 680 nm | LiCor | 926-68072 |
| Donkey Anti-rabbit 800 nm | LiCor | 926-32213 |
| Donkey Anti-goat 680 nm | LiCor | 926-68074 |
