# Supplementary material for: Extracellular vesicles from Kaposi Sarcoma-associated herpesvirus lymphoma induce long-term endothelial cell reprogramming
Source: PLoS Pathog. 2019 Feb 4;15(2):e1007536. doi: 10.1371/journal.ppat.1007536 (PMC6361468; doi:10.1371/journal.ppat.1007536)
Supplement: S2 Table — All genes listed are significantly different from both Input and the Control-EV treatment groups all time points. (DOCX) [file ppat.1007536.s022.docx]

**Table S2**

| **Gene ID** | **Gene Name** | **Log2 Fold Change** | **P value** |
| --- | --- | --- | --- |
| ENSG00000171444.16 | MCC | 0.83 | 0.002 |
| ENSG00000112379.8 | ARFGEF3 | 0.66 | 0.013 |
| ENSG00000163359.14 | COL6A3 | 0.64 | 0.022 |
| ENSG00000275993.2 | CU639417.2 | 0.36 | 0.031 |
| ENSG00000136449.12 | MYCBPAP | 0.33 | 0.040 |
| ENSG00000145431.9 | PDGFC | 0.24 | 0.0081 |
| ENSG00000073737.15 | DHRS9 | 0.23 | 1.2E-06 |
| ENSG00000177410.11 | ZFAS1 | 0.22 | 1.1E-05 |
| ENSG00000180530.8 | NRIP1 | 0.22 | 0.010 |
| ENSG00000166922.7 | SCG5 | 0.21 | 0.00011 |
| ENSG00000128849.10 | CGNL1 | 0.20 | 0.032 |
| ENSG00000198695.2 | MT-ND6 | 0.19 | 0.014 |
| ENSG00000196562.13 | SULF2 | 0.16 | 0.0017 |
| ENSG00000221968.7 | FADS3 | 0.15 | 0.030 |
| ENSG00000189221.8 | MAOA | 0.15 | 0.033 |
| ENSG00000196950.12 | SLC39A10 | 0.13 | 9.45E-05 |
| ENSG00000171056.7 | SOX7 | 0.12 | 0.023 |
| ENSG00000115977.17 | AAK1 | 0.12 | 0.0009 |
| ENSG00000245910.7 | SNHG6 | 0.12 | 0.04 |
| ENSG00000136237.17 | RAPGEF5 | 0.12 | 9.70E-05 |
| ENSG00000108622.9 | ICAM2 | 0.11 | 0.049 |
| ENSG00000231500.5 | RPS18 | 0.11 | 0.013 |
| ENSG00000144118.12 | RALB | 0.10 | 0.019 |
| ENSG00000249264.1 | EEF1A1P9 | 0.10 | 0.049 |
| ENSG00000234741.6 | GAS5 | 0.10 | 3.31E-05 |
| ENSG00000163638.12 | ADAMTS9 | 0.098 | 0.011 |
| ENSG00000101347.8 | SAMHD1 | 0.095 | 0.003 |
| ENSG00000256618.2 | MTRNR2L1 | 0.092 | 0.00051 |
| ENSG00000085662.12 | AKR1B1 | 0.082 | 0.044 |
| ENSG00000109475.15 | RPL34 | 0.081 | 0.0045 |
| ENSG00000099204.17 | ABLIM1 | 0.081 | 6.26E-05 |
| ENSG00000038427.14 | VCAN | 0.080 | 0.003 |
| ENSG00000139116.16 | KIF21A | 0.077 | 0.022 |
| ENSG00000100968.12 | NFATC4 | 0.071 | 0.033 |
| ENSG00000130508.9 | PXDN | 0.066 | 0.016 |
| ENSG00000172493.19 | AFF1 | 0.062 | 0.013 |
| ENSG00000122779.15 | TRIM24 | 0.059 | 0.022 |
| ENSG00000198286.8 | CARD11 | 0.054 | 0.0079 |
| ENSG00000196739.13 | COL27A1 | 0.051 | 0.00043 |
| ENSG00000163682.14 | RPL9 | 0.044 | 0.0045 |
| ENSG00000171863.11 | RPS7 | 0.039 | 0.020 |
| ENSG00000181222.13 | POLR2A | 0.039 | 0.018 |
| ENSG00000132688.10 | NES | 0.035 | 0.038 |
| ENSG00000125534.8 | PPDPF | 0.034 | 0.020 |
| ENSG00000081189.12 | MEF2C | 0.033 | 0.047 |
| ENSG00000269028.3 | MTRNR2L12 | 0.031 | 0.0076 |
| ENSG00000148773.11 | MKI67 | 0.028 | 0.025 |
| ENSG00000118523.5 | CTGF | 0.026 | 0.040 |
| ENSG00000141298.16 | SSH2 | 0.026 | 0.028 |
| ENSG00000125347.12 | IRF1 | 0.024 | 0.049 |
| ENSG00000080345.16 | RIF1 | 0.024 | 0.049 |
| ENSG00000198786.2 | MT-ND5 | 0.021 | 0.032 |
| ENSG00000128284.18 | APOL3 | 0.019 | 0.003 |
| ENSG00000159176.12 | CSRP1 | 0.019 | 0.018 |
| ENSG00000115053.14 | NCL | 0.018 | 0.00037 |
| ENSG00000177954.10 | RPS27 | 0.017 | 0.042 |
| ENSG00000158417.9 | EIF5B | 0.016 | 0.025 |
| ENSG00000211459.2 | MT-RNR1 | 0.015 | 0.010 |
| ENSG00000115306.14 | SPTBN1 | 0.013 | 0.0086 |
| ENSG00000142534.5 | RPS11 | 0.011 | 0.0024 |
| ENSG00000255823.2 | MTRNR2L8 | 0.010 | 0.047 |
| ENSG00000187689.8 | AMTN | 0.010 | 0.00010 |
| ENSG00000013588.5 | GPRC5A | 0.0099 | 0.00041 |
| ENSG00000175061.16 | LRRC75A-AS1 | 0.0082 | 0.020 |
| ENSG00000115648.12 | MLPH | 0.0036 | 0.0087 |
| ENSG00000148677.6 | ANKRD1 | 0.0031 | 0.018 |
| ENSG00000142871.14 | CYR61 | 0.0017 | 0.0045 |
| ENSG00000117523.14 | PRRC2C | -0.00029 | 0.0059 |
| ENSG00000198886.2 | MT-ND4 | -0.00054 | 0.0084 |
| ENSG00000124614.12 | RPS10 | -0.0016 | 0.024 |
| ENSG00000152818.17 | UTRN | -0.0030 | 0.033 |
| ENSG00000210082.2 | MT-RNR2 | -0.0031 | 0.043 |
| ENSG00000140416.18 | TPM1 | -0.0038 | 0.00044 |
| ENSG00000184232.7 | OAF | -0.0053 | 0.049 |
| ENSG00000198888.2 | MT-ND1 | -0.0056 | 1.10E-06 |
| ENSG00000196352.12 | CD55 | -0.0056 | 0.014 |
| ENSG00000198804.2 | MT-CO1 | -0.00653 | 0.047 |
| ENSG00000071054.14 | MAP4K4 | -0.0068 | 0.0024 |
| ENSG00000118181.9 | RPS25 | -0.011 | 0.033 |
| ENSG00000100342.19 | APOL1 | -0.011 | 0.016 |
| ENSG00000205413.6 | SAMD9 | -0.012 | 0.037 |
| ENSG00000106484.13 | MEST | -0.013 | 9.70E-05 |
| ENSG00000106366.8 | SERPINE1 | -0.013 | 0.00018 |
| ENSG00000010278.10 | CD9 | -0.014 | 0.00065 |
| ENSG00000259207.6 | ITGB3 | -0.015 | 0.017 |
| ENSG00000228502.1 | EEF1A1P11 | -0.015 | 0.033 |
| ENSG00000125148.6 | MT2A | -0.016 | 0.00051 |
| ENSG00000168439.15 | STIP1 | -0.023 | 0.049 |
| ENSG00000107562.15 | CXCL12 | -0.027 | 0.00010 |
| ENSG00000110700.5 | RPS13 | -0.031 | 0.041 |
| ENSG00000089159.14 | PXN | -0.033 | 0.024 |
| ENSG00000151491.11 | EPS8 | -0.035 | 0.0066 |
| ENSG00000055332.15 | EIF2AK2 | -0.037 | 0.030 |
| ENSG00000133110.13 | POSTN | -0.038 | 0.0033 |
| ENSG00000235770.4 | LINC00607 | -0.040 | 0.00018 |
| ENSG00000111057.9 | KRT18 | -0.043 | 0.0076 |
| ENSG00000179222.16 | MAGED1 | -0.047 | 0.0050 |
| ENSG00000167191.10 | GPRC5B | -0.049 | 0.0075 |
| ENSG00000171435.12 | KSR2 | -0.050 | 0.046 |
| ENSG00000149503.11 | INCENP | -0.050 | 0.041 |
| ENSG00000143799.11 | PARP1 | -0.051 | 0.032 |
| ENSG00000118432.12 | CNR1 | -0.054 | 3.24E-05 |
| ENSG00000142408.2 | CACNG8 | -0.054 | 0.021 |
| ENSG00000128923.9 | MINDY2 | -0.055 | 0.012 |
| ENSG00000186480.11 | INSIG1 | -0.057 | 0.00039 |
| ENSG00000177464.4 | GPR4 | -0.059 | 0.0050 |
| ENSG00000050165.16 | DKK3 | -0.061 | 3.31E-05 |
| ENSG00000168994.12 | PXDC1 | -0.063 | 0.029 |
| ENSG00000167522.13 | ANKRD11 | -0.063 | 0.047 |
| ENSG00000204387.11 | C6orf48 | -0.068 | 0.0015 |
| ENSG00000164530.12 | PI16 | -0.069 | 0.018 |
| ENSG00000203805.9 | PLPP4 | -0.070 | 0.024 |
| ENSG00000169213.6 | RAB3B | -0.072 | 2.09E-05 |
| ENSG00000135636.12 | DYSF | -0.073 | 2.57E-08 |
| ENSG00000134419.14 | RPS15A | -0.073 | 0.016 |
| ENSG00000113083.11 | LOX | -0.074 | 0.0080 |
| ENSG00000135919.11 | SERPINE2 | -0.074 | 3.31E-05 |
| ENSG00000072274.11 | TFRC | -0.075 | 0.00018 |
| ENSG00000067064.9 | IDI1 | -0.077 | 0.049 |
| ENSG00000185641.6 | AC034236.1 | -0.090 | 0.025 |
| ENSG00000146674.13 | IGFBP3 | -0.094 | 0.0068 |
| ENSG00000135047.13 | CTSL | -0.095 | 0.015 |
| ENSG00000259781.1 | HMGB1P6 | -0.095 | 0.016 |
| ENSG00000230257.1 | NFE4 | -0.10 | 0.041 |
| ENSG00000125817.7 | CENPB | -0.11 | 0.0084 |
| ENSG00000170017.11 | ALCAM | -0.11 | 0.00018 |
| ENSG00000163453.10 | IGFBP7 | -0.11 | 0.00013 |
| ENSG00000140092.13 | FBLN5 | -0.12 | 0.00055 |
| ENSG00000133574.8 | GIMAP4 | -0.13 | 0.019 |
| ENSG00000123200.15 | ZC3H13 | -0.13 | 0.019 |
| ENSG00000131069.18 | ACSS2 | -0.13 | 0.00064 |
| ENSG00000168497.4 | CAVIN2 | -0.13 | 0.012 |
| ENSG00000066629.15 | EML1 | -0.14 | 0.039 |
| ENSG00000247627.2 | MTND4P12 | -0.14 | 0.025 |
| ENSG00000240087.3 | RPSAP12 | -0.14 | 0.036 |
| ENSG00000119969.13 | HELLS | -0.15 | 0.0075 |
| ENSG00000049130.12 | KITLG | -0.16 | 0.0095 |
| ENSG00000170775.2 | GPR37 | -0.17 | 0.0045 |
| ENSG00000156011.15 | PSD3 | -0.17 | 0.041 |
| ENSG00000170365.8 | SMAD1 | -0.17 | 6.26E-05 |
| ENSG00000158859.9 | ADAMTS4 | -0.20 | 0.042 |
| ENSG00000139618.13 | BRCA2 | -0.21 | 0.023 |
| ENSG00000164236.10 | ANKRD33B | -0.27 | 0.038 |
| ENSG00000171223.5 | JUNB | -0.31 | 0.0078 |
| ENSG00000075223.12 | SEMA3C | -0.31 | 0.00042 |
| ENSG00000227063.5 | ENSG00000227063.5 | -0.33 | 0.014 |
| ENSG00000256393.1 | ENSG00000256393.1 | -0.33 | 0.014 |
| ENSG00000264281.3 | ENSG00000264281.3 | -0.33 | 0.014 |
| ENSG00000074211.12 | PPP2R2C | -0.44 | 0.016 |
| ENSG00000107984.8 | DKK1 | -0.56 | 0.015 |
| ENSG00000138944.7 | KIAA1644 | -0.82 | 0.00082 |
| ENSG00000197020.9 | ZNF100 | -0.86 | 0.047 |
