# Supplementary material for: Extracellular vesicles from Kaposi Sarcoma-associated herpesvirus lymphoma induce long-term endothelial cell reprogramming
Source: PLoS Pathog. 2019 Feb 4;15(2):e1007536. doi: 10.1371/journal.ppat.1007536 (PMC6361468; doi:10.1371/journal.ppat.1007536)
Supplement: S3 Table — (DOCX) [file ppat.1007536.s023.docx]

**Table S3**

| **Functional Assay** | **Direction of KSHV-EV Phenotype (Compared to Control)** |
| --- | --- |
| EV Adsorption | Same |
| Cell Migration | Enhanced |
| IL-6 Secretion | Enhanced |
| IRF3/NF-κB Translocation | Same |
| IRF3/NF-κB Dependent Gene Activation | Same |
| STING Activation | Same |
| MEK/ERK1/2 Activation | Enhanced |
| Interferon Stimulatory Gene Expression | Same |
| RNAseq Profile | Unique (both up and down) |
| *De Novo* EV Release | Repressed |
| Cytoskeletal Rearrangement | Same |
| Proliferation | Enhanced |
